# Disentangling the recognition complexity of a protein hub using a nanopore

**DOI:** 10.1038/s41467-022-28465-8

**Published:** 2022-02-21

**Authors:** Lauren Ashley Mayse, Ali Imran, Motahareh Ghahari Larimi, Michael S. Cosgrove, Aaron James Wolfe, Liviu Movileanu

**Affiliations:** 1 Department of Physics, Syracuse University, 201 Physics Building, Syracuse, NY 13244-1130 USA; 2 Department of Biomedical and Chemical Engineering, Syracuse University, 329 Link Hall, Syracuse, NY 13244 USA; 3 Section on Molecular Transport, Eunice Kennedy Shriver National Institute of Child Health and Human Development, National Institutes of Health, Bethesda, MD 20892 USA; 4 Department of Biochemistry and Molecular Biology, State University of New York - Upstate Medical University, 4249 Weiskotten Hall, 766 Irving Avenue, Syracuse, NY 13210 USA; 5 Ichor Life Sciences, Inc, 2651 US Route 11, LaFayette, NY 13084 USA; 6 Lewis School of Health Sciences, Clarkson University, 8 Clarkson Avenue, Potsdam, NY 13699 USA; 7 Department of Chemistry, State University of New York, College of Environmental Science and Forestry, 1 Forestry Dr, Syracuse, NY 13210 USA; 8 The BioInspired Institute, Syracuse University, Syracuse, NY 13244 USA

**Keywords:** Nanoscale biophysics, Single-molecule biophysics, Nanopores, Proteins

## Abstract

WD40 repeat proteins are frequently involved in processing cell signaling and scaffolding large multi-subunit machineries. Despite their significance in physiological and disease-like conditions, their reversible interactions with other proteins remain modestly examined. Here, we show the development and validation of a protein nanopore for the detection and quantification of WD40 repeat protein 5 (WDR5), a chromatin-associated hub involved in epigenetic regulation of histone methylation. Our nanopore sensor is equipped with a 14-residue Win motif of mixed lineage leukemia 4 methyltransferase (MLL4_Win_), a WDR5 ligand. Our approach reveals a broad dynamic range of MLL4_Win_-WDR5 interactions and three distant subpopulations of binding events, representing three modes of protein recognition. The three binding events are confirmed as specific interactions using a weakly binding WDR5 derivative and various environmental contexts. These outcomes demonstrate the substantial sensitivity of our nanopore sensor, which can be utilized in protein analytics.

## Abstract (web summary)

Nanopores are powerful tools for sampling protein-peptide interactions. Here, the authors convert a protein-based nanopore into a sensitive biosensor to characterize the complex binding of WDR5 protein to a 14-residue ligand.

## Introduction

Detailed knowledge of the human genome has stimulated the discovery of over 360 WD40 repeat proteins (WDRs)^1,2^. WDRs are versatile in mediating numerous protein-protein interactions (PPIs) across diverse cellular pathways and play a pivotal regulatory role in scaffolding enzymatic complexes^3,4^. Although they are among the most frequently encountered PPI domains in the human^5,6^ proteome, selective and dynamic interactions of WDRs with dozens of protein substrates are still ambiguous. Furthermore, major perturbations in their physical associations with other proteins can lead to pathological conditions through diverse disease-associated signaling mechanisms^7^. However, it is difficult to appraise the transient nature of these interactions using existing methods due to their restricted time resolution as well as their inability to identify and characterize the heterogeneity of binding specifics^8^. Biological and synthetic nanopores have served as a powerful tool for sampling reversible protein-peptide^9,10^ and protein-protein^11–14^ interactions in solution. Advantages of nanopores using the resistive-pulse technique^15^ include the ability to explore a wide spectrum of kinetic and affinity constants due to an expanded time bandwidth. In addition, nanopore sensors feature the potential for integration into high-throughput technologies^16–18^ and for quantitative determinations in challenging heterogenous solutions^12,13,19–21^. Moreover, these are real-time and label-free measurements that employ selective sensing elements, which are modifiable with atomic precision^22–27^.

The primary challenge in detecting WDRs using a nanopore is the complexity of the interaction. WDRs are too large to enter the nanopore, so these interactions must be probed outside the lumen. Previously, a monomeric β-barrel scaffold of ferric hydroxamate uptake component A (FhuA)^28^ from *Escherichia coli*, named tFhuA, has been used to overcome this challenge^12,13^. A protein ligand was fused to tFhuA via a flexible Gly/Ser-rich hexapeptide tether and a peptide adaptor was attached to the N terminus of ligand to detect protein-protein interactions without steric hindrance^12^. Furthermore, the binding interface between most WDRs and their protein partners are not mediated by a large and relatively flat surface. For example, the 334-residue WD40 repeat protein 5 (WDR5) has a seven-bladed, WD40 repeat-based β propeller circular structure, surrounding a central cavity^4,29^. A segment of the inner lining of this cavity serves as the binding site for the WDR5 interaction sequence (Win) motif of human mixed lineage leukemia (MLL/SET1) methyltrasferases^30–33^. This is also named the Win binding site. A Win motif must enter the WDR5 cavity to reach the Win binding site^32,33^.

**Fig. 1:**
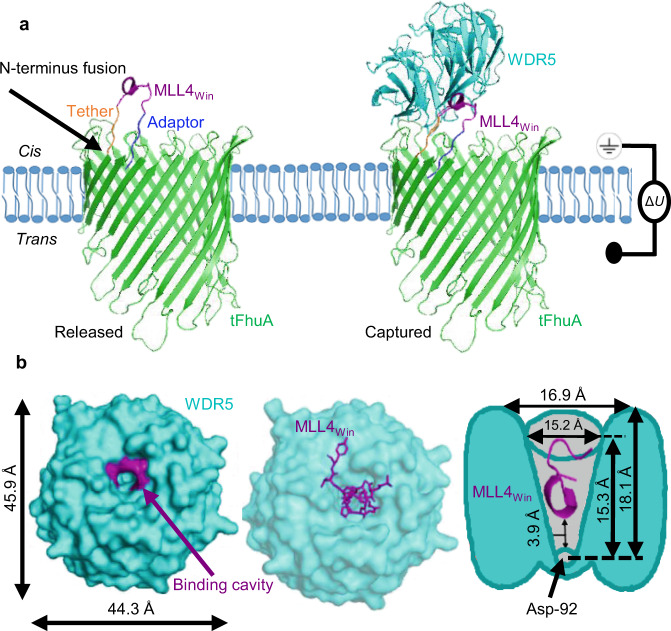
The architecture of MLL4_Win_tFhuA protein nanopore. **a** An MLL4_Win_tFhuA protein nanopore reconstituted into a planar lipid membrane (light blue). This protein comprises a tFhuA protein nanopore (green), a flexible Gly/Ser-rich hexapeptide tether (yellow), a 14-residue MLL4_Win_ peptide ligand (magenta), and a peptide adaptor (blue). This model was developed using Protein Data Bank files 1BY3 (FhuA)^28^ and 4ERZ (MLL4_Win_–WDR5)^32^. The left- and right-hand cartoons show the protein nanopore in the released and WDR5-captured substates, respectively. WDR5 is marked in cyan. The *cis* compartment of the measurement chamber is grounded. A transmembrane potential, Δ*U*, is applied. The black arrow indicates the N terminus of tFhuA that is fused to MLL4_Win_ via the flexible tether. **b** On the left side, a top-view cartoon of WDR5 (in cyan) shows the binding cavity. Phe-133, Cys-261, and Ser-91 are colored in magenta. The central cartoon is the WDR5-MLL4_Win_ complex. MLL4_Win_ (in magenta) occupies the cavity of WDR5. On the right side, there is a cross-section schematic of the conical WDR5 cavity (in gray). MLL4_Win_ (magenta) partitions into the WDR5 cavity. This schematic is not on the same scale with the other cartoons.

In this work, we are able to overcome these arduous challenges and disentangle a multimodal protein recognition process using an engineered protein nanopore. Our tFhuA nanopore is fused to a 14-residue Win motif of mixed lineage leukemia 4 (MLL4_Win_) methyltransferase (Fig. 1a), a WDR5 ligand. This nanopore fusion protein, named MLL4_Win_tFhuA, also includes a peptide adaptor at its N terminus. We demonstrate that MLL4_Win_tFhuA detects WDR5 with single-molecule fidelity in solution. MLL4_Win_ must enter the WDR5 cavity, which has a conical geometry with a maximum internal diameter of ~15 Å, as measured from side chain to side chain (Fig. 1b). Once MLL4_Win_ partitions ~11 Å into the WDR5 cavity, several interactions are coordinated by an evolutionarily conserved Arg residue at position P_0_ of MLL4_Win_ and adjacent side chains of the Win binding site (Supplementary Fig. 1 and Tables 1, 2). We show an unusually large range of kinetics with distinct distributions relating to subpopulations of the transiently formed MLL4_Win_-WDR5 complexes. Biolayer interferometry (BLI) confirms the lower limit of the association rate constant for these complex interactions, yet it is unable to detect multiple binding subpopulations. We also use two WDR5 derivatives and various experimental conditions to validate these multimodal protein recognition events.

## Results and discussion

### A nanopore sensor reveals three distant binding events

**Fig. 2:**
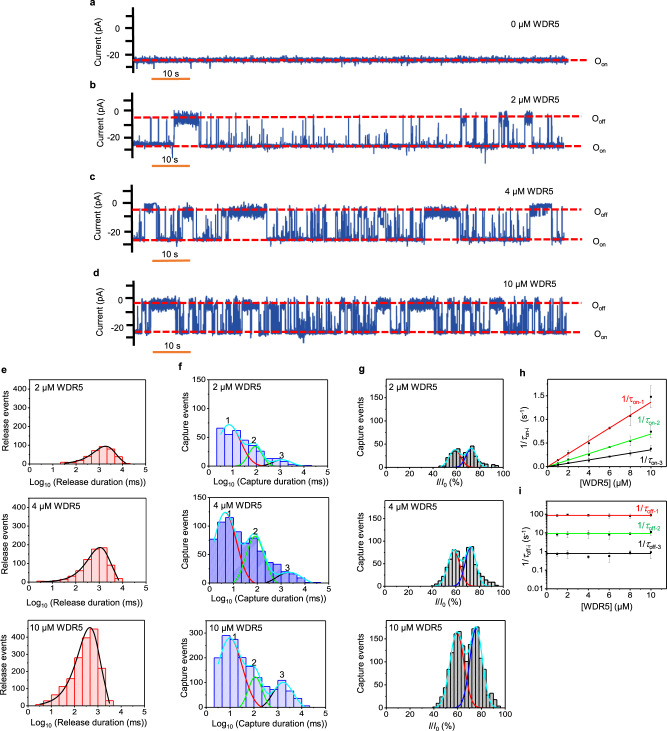
Real-time detection of WDR5. **a** MLL4_Win_tFhuA. **b** MLL4_Win_tFhuA exposed to 2 μM WDR5. **c** MLL4_Win_tFhuA exposed to 4 μM WDR5. **d** MLL4_Win_tFhuA exposed to 10 μM WDR5. O_on_ and O_off_ are release and capture substates, respectively. Data was replicated in three independent experiments. **e** Histograms of release durations, whose values (mean ± s.e.m.) were 2.0 ± 0.2 s (number of events: *N* = 363), 1.2 ± 0.4 s (*N* = 717), and 0.41 ± 0.09 s (*N* = 1791) at 2, 4, and 10 μM WDR5, respectively. **f** Histograms of capture durations. The cumulative fits are marked in cyan. The red, green, and black curves indicate fits for short-, medium-, and long-lived captures, respectively. For 2 μM WDR5, they (mean ± s.e.m.) were 0.009 ± 0.001 s, 0.096 ± 0.001 s, and 1.4 ± 0.1 s, respectively (number of events: *N* = 329). For 4 μM WDR5, they were 0.008 ± 0.001 s, 0.12 ± 0.01 s, and 2.0 ± 0.1 s, respectively (*N* = 717). For 10 μM WDR5, they were 0.010 ± 0.001 s, 0.10 ± 0.01 s, and 1.4 ± 0.1 s, respectively (*N* = 1624). **g** Histograms of normalized current blockades. The cumulative fits are marked in cyan. The red and blue curves indicate fits of smaller and larger blockades, respectively. For 2 μM WDR5, these values (mean ± s.e.m.) were 59 ± 0.9% and 73 ± 0.8%, respectively (number of events: *N* = 329). For 4 μM WDR5, they were 58 ± 0.8% and 73 ± 0.7%, respectively (*N* = 717). For 10 μM WDR5, they were 59 ± 0.6% and 75 ± 0.1%, respectively (*N* = 1607). **h** Dose response of 1*/τ*_on-i_. *τ*_on-1_, *τ*_on-2_, and *τ*_on-3_ are the mean durations between short-, medium-, and long-lived captures, respectively. **i** Dose response of 1*/τ*_off-i_. *τ*_off-1_, *τ*_off-2_, and *τ*_off-3_ are the mean durations of short-, medium-, and long-lived captures, respectively. In (**h**) and (**i**), data represent mean ± s.d. obtained from either *n* = 4 (1 and 2 μM WDR5) or *n* = 3 (4, 6, 8, and 10 μM WDR5) distinct experiments. Source data are provided as a Source Data file.

At a transmembrane potential of −20 mV, a relatively quiet single-channel current was recorded with a single MLL4_Win_tFhuA nanopore (Fig. 2a). The presence of WDR5 in the *cis* compartment at nanomolar concentrations produced infrequent current blockades (Supplementary Fig. 2 Table 3), likely because of the entropic penalty of MLL4_Win_ to partition into the WDR5 cavity. These current blockades occurred as WDR5 was held in the proximity of the pore opening during its reversible captures by MLL4_win_, obstructing a fraction of the ionic flux through the nanopore. They were not noted when an unmodified tFhuA nanopore^12,13^, without the MLL4_Win_ ligand, was exposed to WDR5 added to the *cis* compartment. However, very rare and brief current spikes were observed, likely due to collisions of WDR5 with the opening of the unmodified tFhuA nanopore (Supplementary Figs. 3, 4). Taken together, these findings indicate that WDR5 did not produce significant current blockades due to nonspecific interactions with the *cis* opening of the nanopore. The lack of current blockades without MLL4_win_ shows that the ligand must first bind to WDR5 to enable WDR5-produced current blockades.

WDR5-released and WDR5-captured events recorded with MLL4_Win_tFhuA corresponded to the open-substate, O_on_, and closed-substate, O_off_, respectively. WDR5-captured events were noted in a concentration-dependent manner when WDR5 was added to the *cis* compartment (Fig. 2b–d; Supplementary Fig. 5). These single-channel electrical traces were low-pass filtered at a frequency of 100 Hz using an 8-pole Bessel filter. Yet, WDR5-captured events were not detectable when WDR5 was added to the *trans* compartment (Supplementary Fig. 6). This result agrees with our previous studies^12,13,34^, which showed that tFhuA and its derivatives insert into the membrane with a single orientation. Moreover, WDR5-captured events were not detectable at a positive transmembrane potential of +20 mV (Supplementary Fig. 7). This outcome is in accord with the observation of current blockades produced by the positively charged WDR5 at a negative transmembrane potential (e.g., pI_WDR5_ = 8.27).

Surprisingly, we noted an extensive spectrum of WDR5-captured durations, between ~3 ms and ~40 s. This result stimulated detailed statistical analyses of both the WDR5-released and WDR5-captured durations, whose mean values were denoted by *τ*_on_ and *τ*_off_, respectively. To identify potentially distinct subpopulations of these durations, we employed the maximum likelihood method^35,36^ and logarithm likelihood ratio (LLR) tests^37–39^ to determine the most accurate model of these time constants. Durations of WDR5-released events showed a single-exponential distribution (Fig. 2e). Remarkably, the best model for WDR5-captured durations was a three-exponential distribution (Fig. 2f). Fits to a single-, a two-, or a four-exponential model were not better, as judged by the LLR value. The three WDR5-captured durations were almost one order of magnitude apart from each other. For example, the mean durations of WDR5-captured events were ~12 ms, ~120 ms, and ~1370 ms at 1 μM WDR5 (Supplementary Table 4). We called these short-, medium-, and long-lived events, respectively. Event probabilities of these WDR5-captured events, *P*_1_, *P*_2_, and *P*_3_, respectively, were independent of WDR5 concentration, [WDR5], and followed the inequality: *P*_1_ > *P*_2_ > *P*_3_ (Supplementary Table 5).

Moreover, the mean normalized amplitude of WDR5-produced current blockades, (*I*/*I*_0_), was independent of [WDR5] (*n* = 5 independently reconstituted nanopores; Fig. 2g; Supplementary Table 6). Here, *I*_0_ and *I* denote the single-channel currents of the WDR5-released substate of MLL4_Win_tFhuA and the amplitude of WDR5-produced current blockades, respectively. Histograms of the normalized amplitude of WDR5-produced current blockades showed two peaks, one at ~60% and the other at ~72%. Plots of the normalized amplitude of WDR5-produced current blockades as a function of WDR5-captured duration demonstrate that the short-lived events spanned the broadest range of *I*/*I*_0_ (Supplementary Fig. 8). Earlier studies indicated that a 3_10_-helix is the bound conformation of MLL4_Win_ to the Win binding site^32^. Here, we interpret that MLL4_Win_ may also exhibit conformations that deviate from a 3_10_-helix. Such distinctive and more frequent conformers of a relatively flexible MLL4_Win_ likely generate the heterogeneity of its interactions with WDR5. The wide range of *I*/*I*_0_ of the short-lived events likely correlates to many MLL4_Win_ conformers being able to bring about brief interactions. This interpretation also agrees with the medium- and long-lived events having closely similar *I*/*I*_0_ values with a narrower range of normalized current amplitudes. Therefore, subtle alterations in current fluctuations between event types suggest that various conformers are present and the medium- and long-lived events are more selective as to what conformation MLL4_Win_ must have to lead to a strong binding event.

Here, the association rate constants for short-, medium-, and long-lived events, *k*_on-1_, *k*_on-2_, and *k*_on-3_, respectively, were consistent for all [WDR5] values (Supplementary Table 7). Moreover, the frequency of short, medium-, and long-lived events was proportional to [WDR5] in a ratio 1:1 (Fig. 2h; Supplementary Fig. 9). This outcome indicates a bimolecular association process of the MLL4_Win_-WDR5 complex. The slopes of linear fits of the event frequency, *f* (*f* = 1/*τ*_on_), versus [WDR5] were the corresponding *k*_on_ values. Here, *k*_on-1_, *k*_on-2_, and *k*_on-3_ (mean ± s.e.m.) were (1.4 ± 0.1) × 10^5^ M^−^^1^s^−^^1^, (6.9 ± 1.8) × 10^4^ M^−^^1^s^−^^1^, and (3.6 ± 1.0) × 10^4^ M^−^^1^s^−^^1^, respectively. Dissociation rate constants, *k*_off-i_ (*i* = 1, 2, and 3), which were determined as reciprocal of the mean WDR5-captured durations (1/*τ*_off-i_), were independent of [WRD5] (Fig. 2i; Supplementary Table 8). This result suggests a unimolecular dissociation mechanism of the complex. Subscripts 1, 2, and 3 correspond to short-, medium-, and long-lived events, respectively. Fits of the dissociation rate constants versus [WDR5] resulted in their mean ± s.e.m. values of 86 ± 2 s^−^^1^, 9.2 ± 0.5 s^−^^1^, and 0.78 ± 0.06 s^−^^1^, for short-, medium-, and long-lived WDR5 captures, respectively. The *K*_D_ for short-, medium-, and long-lived interactions were 631 ± 49 μM, 138 ± 18 μM, and 20 ± 4 μM, respectively (Supplementary Table 9).

### Biolayer interferometry validates the slow association process of the MLL4_Win_-WDR5 complex

**Fig. 3:**
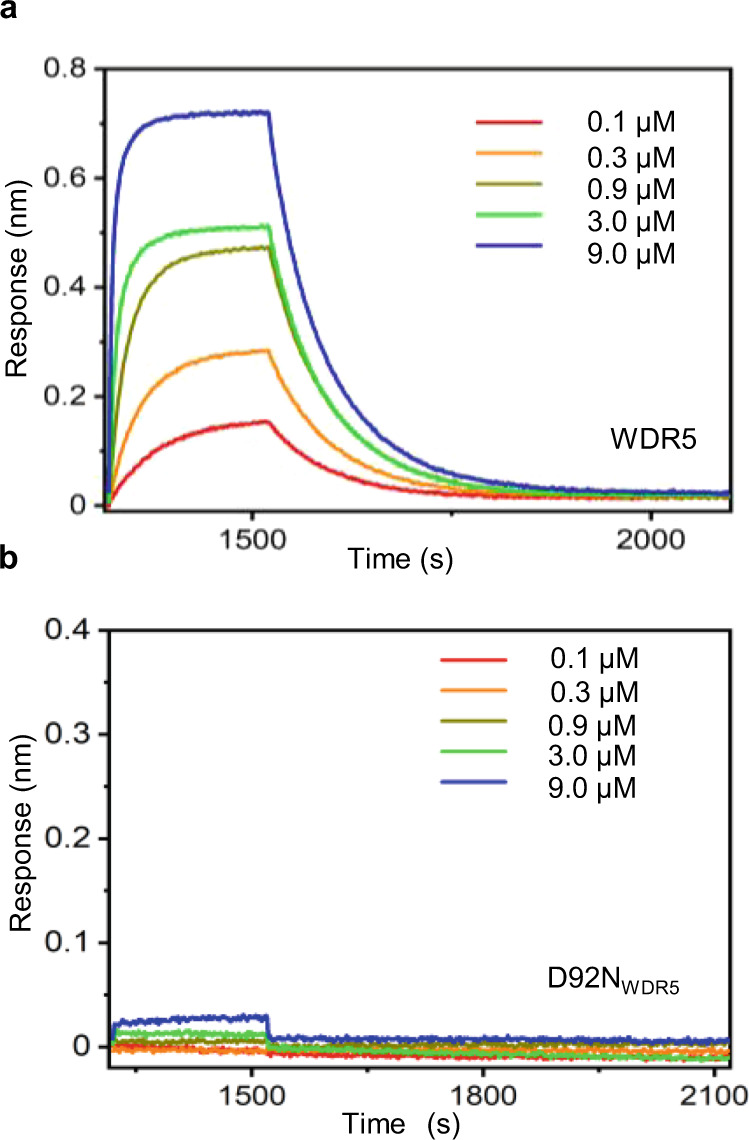
Biolayer interferometry (BLI) sensorgrams of MLL4_Win_-WDR5 interactions. **a** 5 nM biotin-tagged MLL4_Win_ peptide was loaded onto streptavidin sensors for 5 min. Individual binding curves are indicated for [WDR5] from 0.1 µM to 9 µM. **b** The same BLI measurements as described in (**a**), but conducted with D92N_WDR5_. Source data are provided as a Source Data file.

Then, we employed BLI, a real-time approach for determining the kinetics of MLL4_Win_-WDR5 interactions in bulk phase (Methods). Here, we covalently attached MLL4_Win_ onto the BLI sensor and recorded its binding interactions with WDR5. The association and dissociation phases were optically measured using changes in the interference pattern between reflected light waves at the sensor surface (Fig. 3a). The BLI-measured *k*_on_ was (2.6 ± 0.1) × 10^4^ M^−^^1^s^−^^1^ (Supplementary Table 10). The BLI-measured *k*_off_ was (1.2 ± 0.1) × 10^−^^2^ s^−^^1^, to generate a *K*_D_ of (0.46 ± 0.04) × 10^−^^6^ M. BLI was unable to resolve the three binding subpopulations observed when using MLL4_Win_tFhuA and only yielded one *k*_on_ and one *k*_off_. We also employed BLI to conduct a positive-control experiment, testing whether our peptide adaptor exhibits any interaction with WDR5 and found none (Supplementary Fig. 10).

To better understand the mechanistic distinctions of the nanopore and BLI measurements, we simulated the BLI response by using results from single-channel electrical recordings. The simulation shows that the majority of the contribution to the observed BLI response is generated by the long-lived MLL4_Win_-WDR5 interactions (Supplementary Fig. 11). This confirms our tentative interpretation that the BLI sensorgram is extensively biased by the impact of the long-lived binding interactions, despite their lowest event frequency. This simulated curve highlights the superiority of single-molecule measurements using our protein nanopore due to their broad time bandwidth.

### The three binding events involve Win binding site interactions

**Fig. 4:**
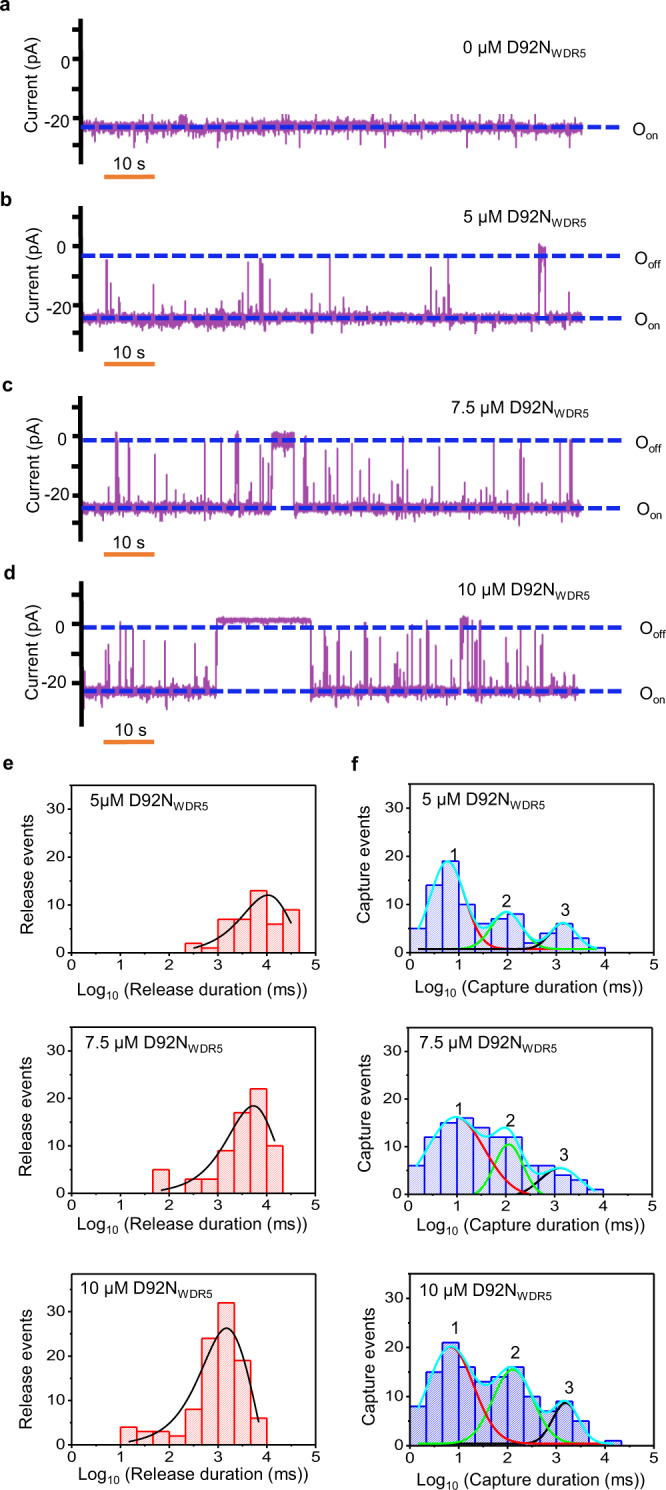
Real-time detection of weakly binding D92N_WDR5_. **a** A single-channel electrical trace of MLL4_Win_tFhuA. **b** MLL4_Win_tFhuA with 5 μM D92N_WDR5_. **c** MLL4_Win_tFhuA with 7.5 μM D92N_WDR5_. **d** MLL4_Win_tFhuA with 10 μM D92N_WDR5_. O_on_ and O_off_ are release and capture substates, respectively. This single-channel electrical signature was replicated in three independent experiments. Single-channel electrical traces were low-pass filtered at a frequency of 100 Hz using an 8-pole Bessel filter. **e** Histograms of D92N_WDR5_-released durations (*τ*_on_) at 5 μM, 7.5 μM, and 10 μM D92N_WDR5_. *τ*_on_ durations (mean ± s.e.m.) were 9.1 ± 3.4 s (number of events: *N* = 45), 4.9 ± 1.0 s (*N* = 69), and 2.2 ± 0.8 s (*N* = 101), respectively. **f** Histograms of D92N_WDR5_-captured durations (*τ*_off_). The cumulative fits are marked in cyan. The red, green, and black curves indicate fits of *τ*_off-1_, *τ*_off-2_, and *τ*_off-3_ for short-, medium-, and long-lived D92N_WDR5_-captured events, respectively. For 5 μM D92N_WDR5_, they (mean ± s.e.m.) were 0.008 ± 0.001 s, 0.13 ± 0.01 s, and 1.6 ± 0.1 s, respectively (number of events: *N* = 78). For 7.5 μM D92N_WDR5_, they were 0.010 ± 0.001 s, 0.12 ± 0.01 s, and 1.9 ± 0.1 s, respectively (*N* = 106). For 10 μM D92N_WDR5_, they were 0.009 ± 0.001 s, 0.19 ± 0.01 s, and 1.8 ± 0.1 s, respectively (*N* = 120). Data was extracted from 20 min recordings. Source data are provided as a Source Data file.

Next, we employed D92N_WDR5_, a WDR5 variant, to find out which event types are related to the partitioning of MLL4_Win_ into the cavity. Also, the detection of D92N_WDR5_ provided proof that our MLL4_win_tFhuA can recognize a somatic cancer mutant of WDR5^40^. Asp-92 is a residue deeply located at the inner tip of the WDR5 cavity (Fig. 1b; Supplementary Fig. 12). First, we used BLI to evaluate MLL4_Win_-D92N_WDR5_ interactions. Interestingly, MLL4_Win_ binds very weakly to D92N_WDR5_, and we were unable to quantify these interactions using BLI (Fig. 3b). D92N_WDR5_ produced current blockades with an amplitude comparable to that of WDR5-captured events (Fig. 4a, b). Moreover, D92N_WDR5_-captured events appeared in a concentration-dependent manner (Fig. 4b–d). As in the WDR5 case, we noted a broad span of D92N_WDR5_-captured event durations. Remarkably, D92N_WDR5_ also showed three distinct binding events, as judged by the LLR test (Fig. 4e, f; Supplementary Table 11). However, the frequency of D92N_WDR5_-captured events was drastically reduced with respect to that value of the WDR5-captured events. This result indicates the influential role of Asp-92 in MLL4_Win_-WDR5 interactions. In addition, the three events had D92N_WDR5_-captured durations similar to those recorded with WDR5. Therefore, the conservation of the three-component binding events illuminates that these subpopulations resulted from interactions deeply located within the WDR5 cavity. Event probabilities of short-, medium-, and long-lived D92N_WDR5_-captured events also followed the inequality: *P*_1_ > *P*_2_ > *P*_3_ (Supplementary Table 12). Again, it is likely that these three interactions correspond to three different modes of D92N_WDR5_ recognition by the flexible MLL4_Win_ ligand^41^. The similarity between D92N_WDR5_ and WDR5 shows that these different MLL4_Win_ conformers were present in the interactions of MLL4_Win_ with each protein. This outcome confirms closely related multimodal binding mechanisms of the two proteins. The D92N mutation influences the *k*_on_, but not the *k*_off_ of short-, medium-, and long-lived D92N_WDR5_-captured events (Supplementary Table 13). Furthermore, the frequency of each of the three event types depended on D92N_WDR5_ concentration, [D92N_WDR5_], in a 1:1 ratio (Supplementary Figs. 13, 14 Table 14). Hence, our findings suggest that the three events were specific binding events. *K*_D_ values corresponded to about one order of magnitude weaker interactions than those obtained with WDR5, regardless of event type. For short-, medium-, and long-lived D92N_WDR5_-produced interactions, *K*_D_ values were 5.4 ± 1.3 mM, 721 ± 18 μM, and 218 ± 22 μM, respectively (Supplementary Table 15). This result reveals that our protein nanopore is able to probe extremely weak interactions in the low millimolar range. Our approach also provides quantitative distinctions between the binding interactions of D92N_WDR5_ and WDR5 (Supplementary Tables 16,17).

Fits of individual rate constants, which are presented above, were conducted with the assumption that no transitions occur among the three capture substates. However, it is not clear whether a kinetic model encompassing interconversion transitions among these capture substates would be better suited for experimentally determined rate constants. An interconversion-dependent kinetic model was created, including six additional rate constants among the capture substates (Supplementary Tables 18, 19 Figs. 15–16). At a confidence level *C* > 0.95, we found that fits to an interconversion-dependent kinetic model were not statistically better than those corresponding to an interconversion-independent kinetic model, as judged by the LLR test.

### A negative charge removal on the WDR5 local surface does not impact the WDR5-MLL4_Win_ interaction

The weak interaction between D92N_WDR5_ and MLL4_Win_ prompted the examination of D172A_WDR5_ (Supplementary Fig. 17), a second WDR5 mutant, which served for an additional positive-control experiment. Here, the goal was to observe how a small alteration in the local surface charge of WDR5 influences the observed kinetic fingerprint of its interactions with MLL4_Win_. The removal of an Asp residue on the protein surface was critical to have a reliable comparison with D92N_WDR5_. D172A_WDR5_ was selected, because it maintains the integrity of the WDR5 cavity while altering the local surface charge of the protein in the proximity of the Win binding site. We found that D172A_WDR5_ produced current blockades with release and capture durations similar to those noted with WDR5 **(**Supplementary Figs. 18, 19; Supplementary Tables 20–25). These results show that the decrease in the *k*_on_ observed with D92N_WDR5_ occurs only because this mutation changes the electrostatic environment in the binding cavity.

### The three protein recognition modes are conserved at a higher transmembrane potential

We then explored whether this kinetic fingerprint of MLL4_Win_^_^WDR5 interactions exists under different experimental conditions. Therefore, we first conducted single-molecule experiments at a transmembrane potential of −40 mV. The addition of WDR5 to the *cis* compartment produced closely similar binding events to those described above (Supplementary Figs. 20, 21). The frequencies of all three WDR5-captured events depended on [WDR5], in a 1:1 ratio, whereas their durations were independent of [WDR5] (Supplementary Fig. 22 and Tables 26–30). This finding suggests that the three observed events were specific binding modes between one tethered MLL4_win_ ligand and one WDR5 protein. The unaltered *k*_off-i_ under this condition with respect to values obtained at a transmembrane potential of −20 mV shows that the integrity of the deep binding interface of WDR5 is not influenced by the transmembrane potential.

### Voltage dependence of MLL4_Win_-WDR5 interactions

**Fig. 5:**
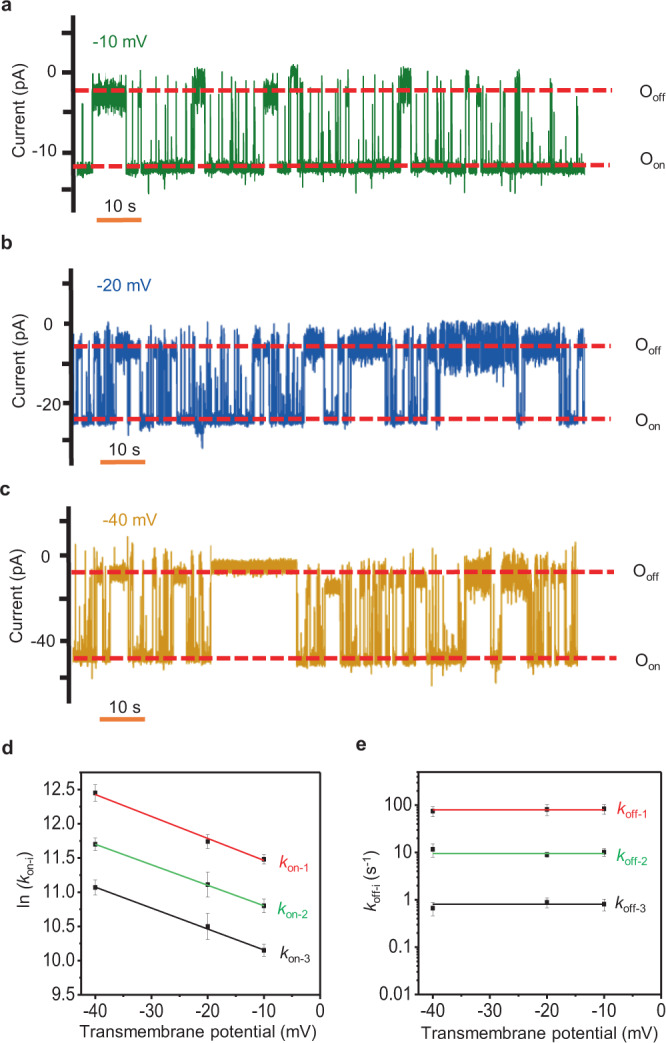
Voltage dependence of MLL4_Win_-WDR5 interactions. **a** Reversible WDR5 captures were observed through current transitions between the O_on_ and O_off_ substates of MLL4_Win_tFhuA at an applied transmembrane potential of −10 mV. **b** The same as in (**a**), but at an applied transmembrane potential of −20 mV. **c** The same as in (**a**), but at an applied transmembrane potential of −40 mV. O_on_ and O_off_ are release and capture substates, respectively. This single-channel electrical signature was replicated in three independent experiments. Single-channel electrical traces in (**a**)–(**c**) were low-pass filtered at 100 Hz using an 8-pole Bessel filter. **d** Linear plot in a semilogarithmic representation, which shows the dependence of ln (*k*_on-i_) on the transmembrane potential, where *i* = 1, 2, and 3. Here, *k*_on-i_, with *i* = 1, 2, and 3, are the association rate constants of short-, medium, and long-lived binding events, respectively. **e** The dependence of *k*_off-i_ on the transmembrane potential. Here, *k*_off-i_, with *i* = 1, 2, and 3, are the reciprocal of mean durations of short-, medium-, and long-lived WDR5 captures, respectively. In (**d**) and (**e**), data points represent mean ± s.d. obtained from *n* = 3 distinct experiments. 8 μM [WDR5] was added to the *cis* compartment. Source data are provided as a Source Data file.

WDR5 has a slightly positive charge under our experimental conditions and binding interactions occur near the nanopore opening. Therefore, we hypothesized that the *k*_on-i_ is voltage dependent, so we compared single-channel electrical traces at various transmembrane potentials when 8 μM [WDR5] was kept unchanged in the *cis* compartment. In accord with our expectation, the three WDR5 capture rate constants, *k*_on-i_ (*i* = 1, 2, and 3), increased at elevated negative transmembrane potentials (Fig. 5a–d and Supplementary Figs. 23, 24, Table 31). Our detailed event analyses also confirmed that the *k*_off-i_ (*i* = 1, 2, and 3) and distribution probabilities of the three protein recognition events were independent of the transmembrane potential (Fig. 5e and Supplementary Tables 32, 33). In addition, a semilogarithmic representation of the voltage dependence of all *k*_on-i_ values revealed an identical slope for the three binding events, suggesting a similar mechanism of these WDR5 recognition modes. Hence, this enabled the calculation of the association rate constants at a zero transmembrane potential for the three binding events, *k*_on-i_ (0) (Supplementary Table 34). For example, the lower-limit association constant of the long-lived events at a zero transmembrane potential, *k*_on-3_ (0), was (1.9 ± 0.2) × 10^4^ M^−^^1^s^−^^1^, which compares well with the BLI-determined *k*_on_ under similar buffer conditions (Supplementary Table 10). The reduction in the activation free energy of all WDR5-released events, ΔΔ*G*_on_, was ~0.7 kcal/mol at a transmembrane potential of −40 mV with respect to that value determined at a zero transmembrane potential (Supplementary Table 35). This data was used to determine a small relative net charge, *z*, of ~0.8 for WDR5 (Supplementary Table 36).

### Quantitative and conceptual comparisons of nanopore sensing with BLI

In a recent study^42^, we have shown that the *k*_on_ values of Win motif peptides with WDR5 are influenced by their tethering on a sensor surface. In addition, the partitioning of MLL4_Win_ into the WDR5 cavity significantly reduces the *k*_on_ due to the entropic penalty of this process^32^. The nanopore-determined *k*_on_ ranged between 10^4^ and low 10^5^ M^−^^1^s^−^^1^. These values and the BLI-determined *k*_on_ value are at least two orders of magnitude lower than one would expect for the *k*_on_ of peptide-protein complexes (10^7^–10^8^ M^−^^1^s^−^^1^)^43,44^. Interestingly, the BLI-determined *k*_on_ was similar to *k*_on-3_ probed by nanopore recordings. This finding suggests that the similar tethering and entropic penalty in both techniques influence the *k*_on_. On the other hand, the BLI-determined *k*_off_ was much lower than all nanopore-inferred *k*_off-i_ values. Yet, it must be noted that the inability of BLI to distinguish the three binding events and its relatively limited time resolution make it challenging to compare dissociation rate constants. Instead, our approach can be employed to detect a significantly weaker interaction than that probed by BLI. This protein nanopore is the only tool sensitive enough to resolve the three modes of protein recognition in real time.

### Distinctive outcomes with WDR5 and D92N_WDR5_

**Fig. 6:**
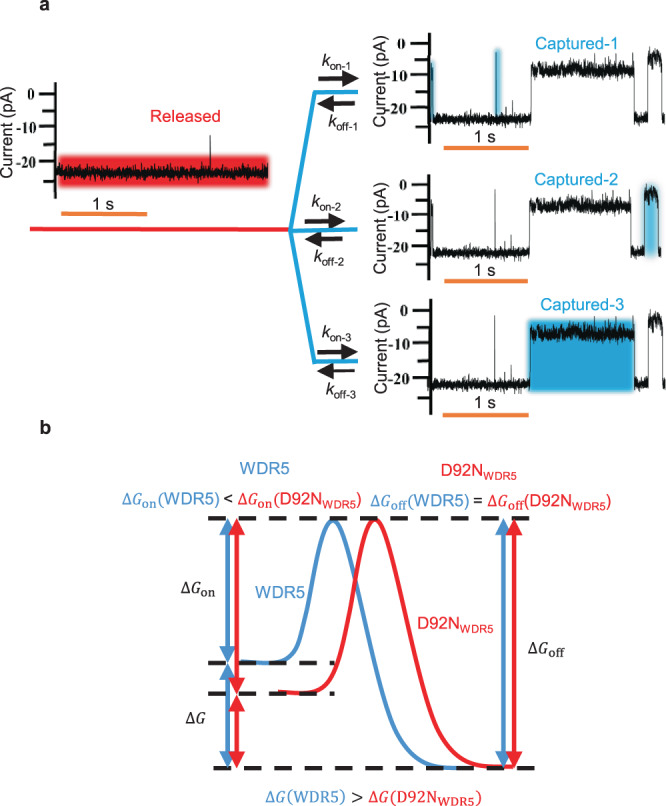
Kinetic model of MLL4_Win_-WDR5 interactions. **a** This model includes the open current substate, O_on_ (highlighted in red), and the three protein recognition events, O_off_ (highlighted in blue). **b** Model of the free energy landscape of MLL4_Win_-WDR5 interactions for both WDR5 and D92N_WDR5_.

In this work, we developed a modular protein nanostructure capable of discriminating between three individual kinetic components existing in a complex binding distribution (Fig. 6a). Furthermore, a weakly binding mutant, D92N_WDR5_, confirmed the three-binding event distribution. D92N_WDR5_ does not show an altered *k*_off_, because Asp-92 does not form any stabilizing bonds with MLL4_Win_^32,33^. Only Cys-261, Phe-133, Ser-91, and Asp-107 are responsible for the stability of this interaction (Supplementary Fig. 1 Table 2). Therefore, both proteins show a similar activation free energy of dissociation, Δ*G*_off_ (Fig. 6b). However, Asp-92 of WDR5 does provide a negative charge to assist with the electrostatic pulling of the critical Arg residue of MLL4_Win_ into the WDR5 cavity (Supplementary Table 1 Fig. 12)^45^. In contrast, Asn-92 of D92N_WDR5_ does not exert this role, so the activation free energy barrier of association, Δ*G*_on_, is significantly amplified, reducing the frequency of reversible binding events.

### Advantages of this protein nanopore

Our nanopore experiments with WDR5, D92N_WDR5_, and D172A_WDR5_, along with prior crystallographic studies^32,33^, suggest that all three binding events are specific interactions and occur deeply within the WDR5 cavity. In addition, both D92N_WDR5_ and D172A_WDR5_ showed normalized current blockades and capture durations similar to those noted with WDR5 (Fig. 2g; Supplementary Figs. 8 and 25, Tables 4, 6, 11, 20, 22, and 37). Through this cascade of binding scenarios, we show that our protein nanopore is capable of sampling complex binding interfaces. It could also be used to study other WDRs and groove-containing binding systems. These include unfolded protein chains entering chaperone tunnels, such as those of the GroEL^46^ and Clp^47^ families. Furthermore, we show that our protein nanopore can also probe very weak interactions with affinities up to low micromolar. Again, this substantially extends the application spectrum of our nanopore and highlights its significant sensitivity. For example, the interaction details observed in our study could only be observed at single-molecule precision. Other bulk-phase techniques, such as isothermal titration calorimetry (ITC)^48^ and surface plasmon resonance (SPR)^49^, can be used to study these peptide-protein interactions, but they have a narrower time bandwidth. These limitations of ensemble studies prevent us from identifying binding details like those present in a realistic biological system.

### Implications and prospects in biotechnology

Recently, it has been discovered that the high-affinity Win binding site of WDR5 is a key player in transient interactions with dozens of proteins^50^. These include 3-phosphoinositide-dependent protein kinase 1 (PDPK1) and proteins involved in phosphatidylinositol 3-kinase (PI3K) signaling. Here, we speculate that WDR5 selectively utilizes the Win binding site. For example, it is likely that the long-lived interactions of WDR5 have physiological relevance for the functional integrity of large multi-subunit complexes of methyltransferases^51^, which is required for optimal enzymatic activity^52–54^. On the other hand, weaker interactions of WDR5 with other proteins may assist in cell signaling pathways^50^. Hence, the wide range of binding affinities of these specific interactions is in accord with the multitasking feature of the Win binding site in subnuclear PPIs^55,56^. Furthermore, there is significant interest in exploring the Win binding site, because WDR5 is implicated in numerous cancers^57,58^. Therefore, our protein nanopore can serve as a tool to acquire real-time and comprehensive pharmacokinetics for these clinically important interactions.

In summary, we probed the complexity of MLL4_Win_-WDR5 interactions with no steric hindrance of nanopore confinement. The heterogeneous event distribution unambiguously revealed three distant modes of protein recognition with diverse affinities. The interactions of MLL4_Win_ with a weakly binding WDR5 derivative were not quantifiable using BLI, but our nanopore sensor provided a complete characterization of the binding kinetics. This unusual kinetic fingerprint of MLL4_Win_-WDR5 interactions was further confirmed and fully characterized at varying voltage biases. Therefore, this study demonstrates that our approach can illuminate a wide span of multimodal protein recognition events and strengthen quantitative protein interaction studies.

## Methods

### Modular genetic engineering of the protein nanopore

A plasmid with *omll4tfhua* as the gene of interest was purchased from GenScript (Piscataway, NJ). This gene included a DNA sequence encoding, from the N to C terminus, a 13-residue peptide adaptor (MGDRGPEFELGTM), a 14-residue MLL4 Win motif peptide (MLL4_Win_, LNPHGAARAEVYLR**)**, a 6-residue Gly/Ser-rich peptide tether, and a 455-residue large truncation of *Ferric hydroxamate uptake component* A of *Escherichia coli* (tFhuA)^12^. The pPR-IBA1 vector was used as the template. The MLL4_Win_ represented a recognition element for target analytes WDR5, D92N_WDR5_, and D172A_WDR5_. The peptide adaptor was slightly negatively charged and unstructured in solution^59^. This 488-residue modular nanopore was named MLL4_Win_tFhuA.

### Protein expression and purification

The plasmid mentioned above was transformed into *E. coli* BL21(DE3) cells^13,34^. All transformed cells were grown in a Luria-Bertani medium at 37 °C until the OD_600_ reached a value of ~0.4. Then the cells were induced using 1 mM isopropyl β-D-1-thiogalactopyranoside (IPTG) and left for further culturing for ~5 h at 37 °C. Next, the cells were centrifuged at 3700 × *g* for 30 min at 4 °C, then resuspended in 300 mM KCl, 20 mM Tris-HCl, 5 mM ethylenediaminetetraacetic acid (EDTA), pH 8. Cell lysis was achieved using a microfluidizer (Model 110 L; Microfluidics, Newton, MA). The cell lysate was centrifuged at 108,500 × *g* for 30 min at 4 °C to separate the insoluble pellet and supernatant. The supernatant was discarded, and the water-insoluble modular protein remained in the pellet. The pellet encapsulated the protein inclusion bodies and went through a series of 1.5% Triton X-100, 1 mM EDTA washes in order to remove cellular debris. The supernatant was centrifuged at 108,500 × *g* for 30 min at 4 °C to separate target insoluble protein from the water-soluble cellular debris. The precipitate was solubilized in 8 M urea. Then, the solubilized protein was further purified on an anion-exchange column (Q12-Sepharose; Bio-Rad, Hercules, CA) using a linear salt gradient of 0–1 M KCl, 20 mM Tris-HCl, pH 8. The peak fractions were collected, combined, and centrifuged at 3700 × *g* for 10 min at 4 °C to separate precipitated proteins. These fractions were passed through a size-exclusion column (HiLoad16/600 Superdex-75; GE Healthcare Life Sciences, Pittsburg, PA) for a final purification step. The fractions correlating to the target protein size were combined and centrifuged 3700 × *g* for 10 min at 4 °C to remove aggregated protein. The supernatant was removed and prepped for lyophilization. The protein purity was assessed using SDS-PAGE analyses.

WDR5, D92N_WDR5_, and D172A_WDR5_ were expressed using Rosetta II pLysS (Novagen via Millipore Sigma, Burlington, MA) competent cells^30,60^. All transformed cells were grown in Luria-Bertani medium at 37 °C until the OD_600_ reached 0.75 and cells were then chilled at 4 °C until they reached an OD_600_ value of ~1.0. The cells were induced with 1 mM IPTG and left for further growth for 18–20 h at 16 °C. Cells were centrifuged at 4000 × *g* for 30 min at 4 °C to harvest the pellet. The pellet was resuspended in 300 mM KCl, 50 mM Tris, pH 7.4, 3 mM dithiothreitol (DTT), 30 mM imidazole, 0.1 mM phenylmethylsulfonyl fluoride (PMSF), and one EDTA-free protease inhibitor cocktail tablet (cOmplete; Sigma Aldrich, St. Louis, MO). Cell lysis was achieved using a microfluidizer (Model M110L; Microfluidics, Newton, MA). The lysate was cleared by centrifuging at 108,500 × *g* at 4 °C for 35 minutes. The supernatant containing WDR5, D92N_WDR5_, or D172A_WDR5_ was passed through an immobilized metal-affinity column (5 mL, Bio-Scale Mini Profinity IMAC cartridge; Bio-Rad, Hercules, CA). The protein was then eluted with a 20-column volume linear gradient of imidazole until the final buffer was 300 mM KCl, 50 mM Tris-HCl, pH 7.4, 3 mM DTT, and 500 mM imidazole. Then an SDS-PAGE gel was run to determine which fractions to collect for further purification. Tobacco Etch Virus (TEV) protease (New England Biolabs, Ipswich, MA) was used to remove the hexahistidine tag on the protein. After TEV digestion, the protein sample was dialyzed against 300 mM KCl, 50 mM Tris, pH 7.4, 3 mM DTT and 30 mM imidazole, so it could be passed over the immobilized metal-affinity column (5 mL, Bio-Scale Mini Profinity IMAC cartridge; Bio-Rad) for a second run. Finally, protein samples were concentrated using a 10 kDa-molecular weight cut-off spin concentrator (Millipore Sigma, St. Louis, MO). The concentrated samples went through a final gel-filtration purification process using a size-exclusion column (HiLoad16/600 Superdex-75;GE Healthcare Life Sciences, Pittsburg, PA). The final sample buffer was 300 mM KCl, 50 mM Tris, 1 mM tris(2-carboxyethyl)phosphine (TCEP), pH 7.4. The concentrated protein sample was run on an SDS-PAGE gel to confirm size and purity.

### Protein refolding

MLL4_Win_tFhuA was solubilized from a lyophilized form in 200 mM KCl, 8 M urea, 20 mM Tris-HCl, pH 8 to a concentration of ~25 µM and incubated at room temperature for at least one hour. After protein quantification via molar absorptivity, *n*-dodecyl-β-D-maltopyranoside (DDM; Anatrace, Maumee, OH) was added to the denatured samples to a final concentration of 1% (w/v). The solubilized protein was placed in a dialysis bag with a 14 kDa molecular weight cut off, then dialyzed against the optimized refolding buffer, which was 200 mM KCl, 20 mM Tris-HCl, pH 8, at 4 °C for at least 72 h. The dialysis solution was replaced once every 24 h. This refolded protein sample was then centrifuged to eliminate any precipitation of unfolded proteins. The supernatant was separated as the working sample for single-channel electrical recordings. Protein quantification was determined using molar absorptivity at a wavelength of 280 nm.

### Biolayer interferometry

These experiments were executed using an Octet Red384 instrument (FortéBio, Fremont, CA)^42^. MLL4_Win_ was biotinylated and amidated at the N and C terminus, respectively. A buffer solution containing 300 mM KCl, 20 mM Tris-HCl, 1 mM TCEP, 1 mg/ml bovine serum albumin (BSA), pH 7.5 was used to soak streptavidin (SA) sensors for 30 min. A flexible (GGS)_3_ linker was inserted between the MLL4_Win_ sequence and the biotinylated site. In this way, there was a satisfactory distance between MLL4_Win_ and the sensor surface for either WDR5 or D92N_WDR5_ to interact without steric restriction from the BLI sensor. 5 nM Biotinyl-(GGS)_3_MLL4_Win-NH2_ was loaded onto the sensors for 15 min. Washing off the unbound peptides was achieved by dipping the sensors into a peptide-free buffer for 5 min. A 3-fold serial dilution of either WDR5 or D92N_WDR5_, which ranged between 0.1 µM and 9 µM, was executed for inspecting the association process. Then, the BLI sensors were placed in a protein analyte-free buffer solution for inspecting the dissociation process. For all WDR5 and D92N_WDR5_ concentrations, the peptide-free BLI sensors were run in parallel as controls. These controls were used to subtract the baseline and drift in the sensorgrams. This process was required to extract the binding curves. All BLI experiments were conducted at 24 °C. For the fitting of binding curves, the FortéBio Octet Data Analysis software (FortéBio) was used. The curves generated by the association process were fitted using the following equation^61^:

1 $$Y={Y}_{{{{{{\rm{\infty }}}}}}}-\left({Y}_{{{{{{\rm{\infty }}}}}}}-{Y}_{0}\right){e}^{-{k}_{{{{{{\rm{obs}}}}}}}t}$$

Here, *Y*_0_ and *Y*_∞_ are the response signals during the association process at zero and infinity times, respectively. *t* is the cumulative time of the association reaction. *k*_obs_ denotes the apparent first-order reaction rate constant of the association process. The curves generated by the dissociation process were fitted using the following equation:

2 $$Y={Y}_{{{{{{\rm{\infty }}}}}}}+\left({Y}_{0}-{Y}_{{{{{{\rm{\infty }}}}}}}\right){e}^{-{k}_{{{{{{\rm{off}}}}}}}t}$$

where, *Y*_0_ and *Y*_∞_ are the response signals during the dissociation process at zero and infinity times, respectively. *k*_off_ shows the dissociation rate constant. The association rate constant, *k*_on_, was included into the following function:

3 $${k}_{{{{{{\rm{obs}}}}}}}=\,{k}_{{{{{{\rm{on}}}}}}}\left[C\right]+{k}_{{{{{{\rm{off}}}}}}}\,$$

where [*C*] is the protein analyte concentration. Global fitting, which was conducted using several protein analyte concentrations, provided the corresponding *k*_on_ and *k*_off_ values. These kinetic parameters were employed to calculate the equilibrium dissociation constant, *K*_D_. Three independent BLI measurements were performed for quantitative kinetic determinations.

### Simulation of the BLI response using results from single-channel electrical recordings

A model for multimodal protein recognition events was created using a competing-reaction method^62^. Each event was modelled as resulting from a different analyte competing to bind to the sensor. Consequently, the analytes were assumed to have the same masses and concentrations. The following differential equations were used to simulate the results:

4 $$\frac{{{{{{\rm{d}}}}}}{R}_{1}}{{{{{{\rm{d}}}}}}t}=\,{k}_{{{{{{\rm{on}}}}}}-1}[C]\left({R}_{{{\max }}}-\,{R}_{1}-\,{R}_{2}-\,{R}_{3}\right)-\,{k}_{{{{{{\rm{off}}}}}}-1}{R}_{1}$$

5 $$\frac{{{{{{\rm{d}}}}}}{R}_{2}}{{{{{{\rm{d}}}}}}t}=\,{k}_{{{{{{\rm{on}}}}}}-2}[C]\left({R}_{{{\max }}}-\,{R}_{1}-\,{R}_{2}-\,{R}_{3}\right)-\,{k}_{{{{{{\rm{off}}}}}}-2}{R}_{2}$$

6 $$\frac{{{{{{\rm{d}}}}}}{R}_{3}}{{{{{{\rm{d}}}}}}t}=\,{k}_{{{{{{\rm{on}}}}}}-3}[C]\left({R}_{{{\max }}}-\,{R}_{1}-\,{R}_{2}-\,{R}_{3}\right)-\,{k}_{{{{{{\rm{off}}}}}}-3}{R}_{3}$$

Here *R*_1_, *R*_2_ and *R*_3_ correspond to the BLI responses from short-, medium- and long-lived events, respectively. [*C*] denotes the concentration of the protein analyte. *R*_max_ indicates the maximum BLI response determined by the total concentration of the immobilized partner. The total simulated BLI response at any given time, *R*, was given by:

7 $$R=\,{R}_{1}+\,{R}_{2}+\,{R}_{3}$$

Kinetic rate constants obtained from single-channel electrical recordings were plugged into these equations to obtain the expected BLI sensorgram. *R*_max_ was adjusted to match the simulated and experimental time-dependent BLI curves at equilibrium. A custom code and a mathematical algorithm were developed using MATLAB R2021a (MathWorks, Natick, MA).

### Single-channel electrical recordings

Single-molecule electrophysiology was conducted using planar lipid bilayers^39,63^. A 25 µm-thick Teflon septum (Goodfellow Corporation, Malvern, PA) separated the two half sides of the chamber. A 90 μm-diameter aperture was created in this Teflon partition, which was then pretreated with hexadecane (Sigma-Aldrich, St. Louis, MO) dissolved in highly purified pentane (Fisher HPLC grade, Fair Lawn, NJ). A planar lipid bilayer was made of 1,2-diphytanoyl-*sn*-glycero-phosphatidylcholine (Avanti Polar Lipids, Alabaster, AL) across the small aperture. For all experiments, the buffer solution contained 300 mM KCl, 20 mM Tris-HCl, 1 mM TCEP, pH 7.5. Samples of protein nanopores and analytes were added to the *cis* compartment, which was grounded. The nanopore samples were added at a final concentration between 0.3 and 1 ng/µl. An Axopatch 200B patch-clamp amplifier (Axon Instruments, Foster City, CA) was used to acquire single-channel electrical currents. The applied transmembrane potential was −20 mV, unless otherwise stated. The electrical signal was low-pass filtered using an 8-pole Bessel filter (Model 900; Frequency Devices, Ottawa, IL) at a frequency of 10 kHz, then digitized using a low-noise acquisition system (Model Digidata 1440 A; Axon Instruments) and sampled at a frequency of 50 kHz. For the analysis of binding events, single-channel electrical traces were filtered at a frequency of 1 kHz. All single-channel electrical recordings were acquired at room temperature (23 ± 1 °C).

### Statistical analysis of single-channel substate events

pClamp 10.7 (Axon Instruments) was used for data acquisition and analysis. ClampFit 10.7 (Axon) and Origin 8.6 (OriginLab, Northampton, MA) were used to produce figures. Different models were tested for both capture and release event durations. For each model, a kinetic rate matrix was employed to generate the probability distribution function (PDF), so the kinetic rates were obtained by fitting the data using the maximum likelihood method^35,36^. A logarithm likelihood ratio (LLR) test was used to compare the results from different models and determine the number of statistically significant peaks that are best suited to the data^37–39^. At a confidence number of 0.95, the best model for the release durations was a single-exponential fit. On the contrary, the best model for the capture durations was a three-exponential fit.

### Molecular graphics

All cartoons that illustrate molecular graphics were prepared using the PyMOL Molecular Graphics System (Version 2.4.0 Schrödinger, LLC).

### Reporting summary

Further information on research design is available in the Nature Research Reporting Summary linked to this article.

## Supplementary information

Supplementary Information

Reporting Summary

## Data Availability

In addition to Supplementary Information file, data supporting the findings of this article have been deposited in Zenodo at 10.5281/zenodo.5813135. The source data underlying Figs. 2e–i, 3a, b, 4e, f, 5d, e, and Supplementary Figs. 4, 8–10, 13, 14, 19, 21–25, and Supplementary Tables 4–7, 9–16, 19–23, & 25–37 are provided in the Source data file. Entries 1BY3 and 4ERZ from the Protein Data Bank were used in this article for molecular visualizations. Source data are provided with this paper.

## Source data

Source Data

## Code availability

All custom codes and mathematical algorithms used in this study are available at 10.5281/zenodo.5813135.
