# Supplementary material for: Disentangling the recognition complexity of a protein hub using a nanopore
Source: Nat Commun. 2022 Feb 21;13:978. doi: 10.1038/s41467-022-28465-8 (PMC8861093; doi:10.1038/s41467-022-28465-8)
Supplement: Supplementary file 1 — Supplementary Information [file 41467_2022_28465_MOESM1_ESM.pdf]

# SUPPLEMENTARY INFORMATION FILE

# Disentangling the Recognition Complexity of a Protein Hub Using a Nanopore

Lauren Ashley Mayse[1,2], Ali Imran[1], Motahareh Ghahari Larimi[1,3], Michael S. Cosgrove[4], Aaron James Wolfe[1,5,6,7] & Liviu Movileanu[1,2,8,*]

[1]*Department of Physics, Syracuse University, 201 Physics Building, Syracuse, New York 13244-1130, USA*

[2]*Department of Biomedical and Chemical Engineering, Syracuse University, 329 Link Hall, Syracuse, New York 13244, USA*

[3]*Section on Molecular Transport, Eunice Kennedy Shriver National Institute of Child Health and Human Development, National Institutes of Health, Bethesda, MD 20892, USA*

[4]*Department of Biochemistry and Molecular Biology, State University of New York - Upstate Medical University, 4249 Weiskotten Hall, 766 Irving Avenue, Syracuse, New York 13210, USA*

[5]*Ichor Life Sciences, Inc., 2651 US Route 11, LaFayette, New York 13084, USA*

[6]*Lewis School of Health Sciences, Clarkson University, 8 Clarkson Avenue, Potsdam, New York 13699, USA*

[7]*Department of Chemistry, State University of New York, College of Environmental Science andForestry, 1 Forestry Dr., Syracuse, New York 13210, USA*

[8]*The BioInspired Institute, Syracuse University, Syracuse, New York 13244, USA*

***Keywords:*** FhuA; Ion channel; Protein dynamics; Single-molecule electrophysiology; Membrane protein engineering; Protein detection.

[*]The corresponding author's contact information:
Liviu Movileanu, PhD, Department of Physics, Syracuse University, 201 Physics Building, Syracuse, New York 13244-1130, USA. Phone: 315-443-8078;
E-mail: lmovilea@syr.edu

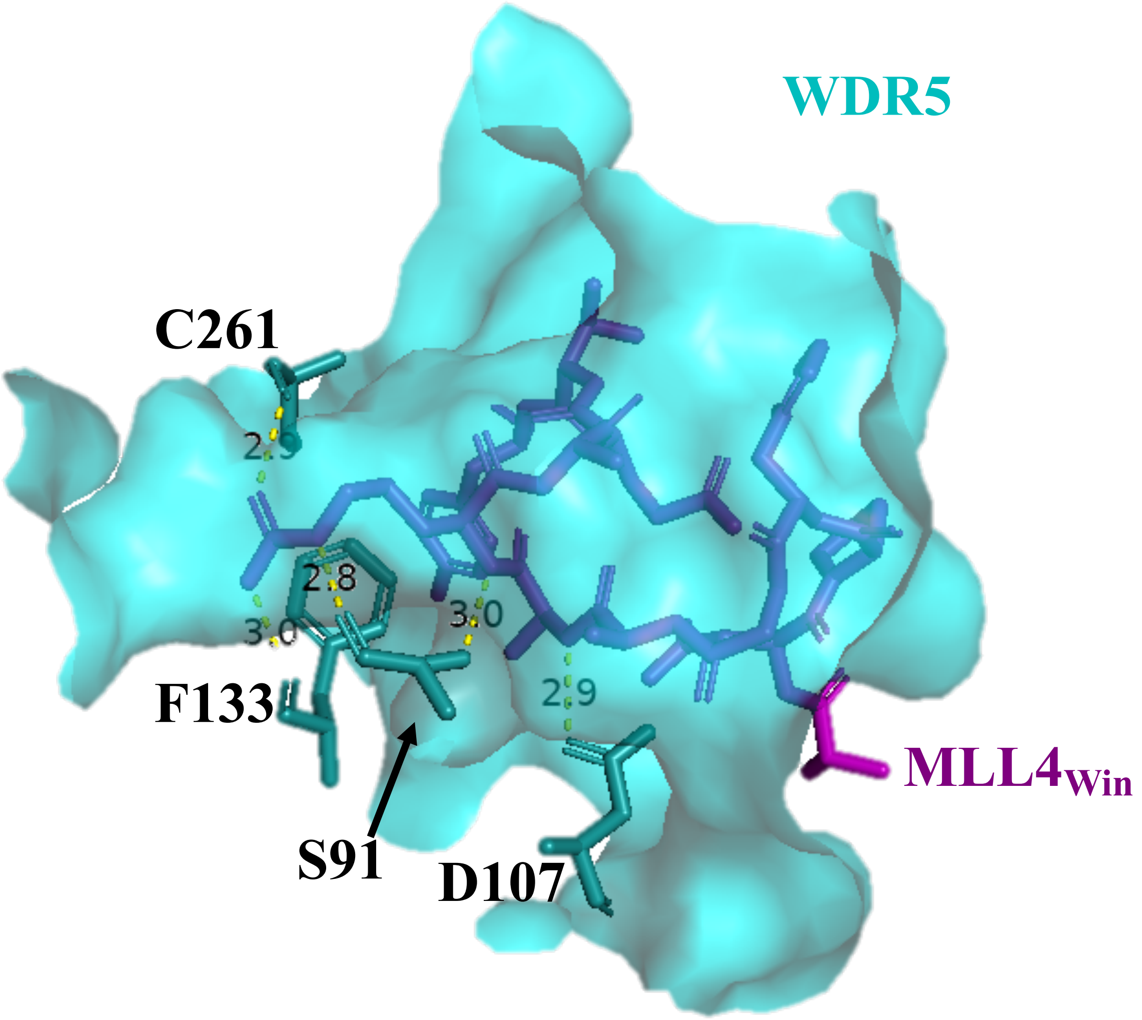


**Supplementary Figure 1. A side-view cartoon of the WDR5 cavity (in cyan) and MLL4$_{Win}$ (in magenta/blue) binding to the WDR5 cavity.** Cys-261, Phe-133, and Ser-91 create hydrogen bonds (dashed lines) with an Arg residue in position $P_0$ of MLL4$_{Win}$.[1] Asp-107 also forms a hydrogen bond with Ala at position $P_{-1}$ of MLL4$_{Win}$ (Supplementary Tables 1-2).

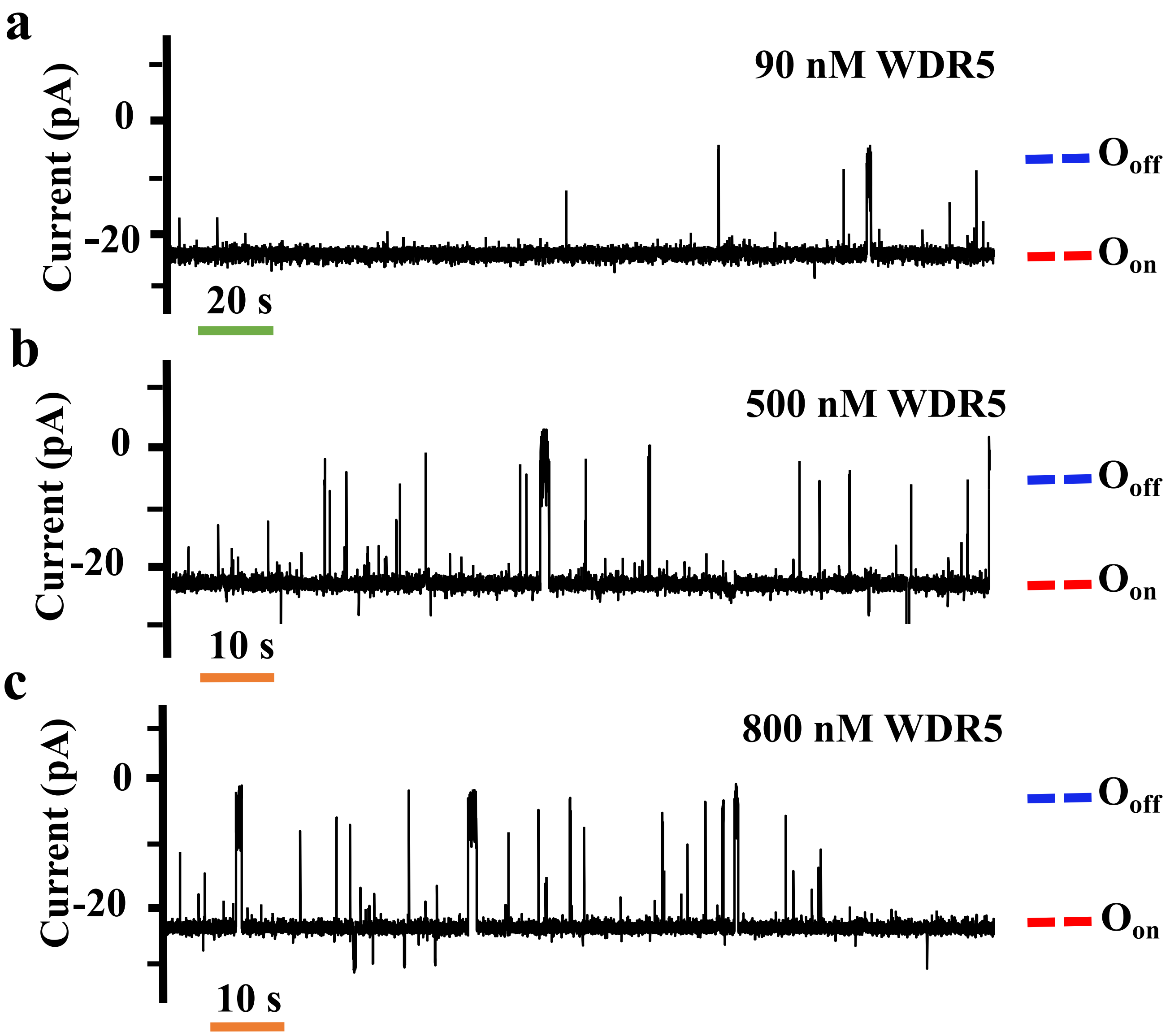


**Supplementary Figure 2. Measuring reversible MLL4$_{Win}$-WDR5 interactions at nanomolar WDR5 concentrations. (a)** Reversible WDR5 captures were observed through current transitions between the $O_{on}$ and $O_{off}$ substates when 90 nM WDR5 was added to the *cis* compartment. **(b)** MLL4$_{Win}$tFhuA with 500 nM WDR5. **(c)** MLL4$_{Win}$tFhuA with 800 nM WDR5. In traces (a) - (c), the applied transmembrane potential was −20 mV. Single-channel electrical traces were low-pass filtered at 100 Hz using an 8-pole Bessel filter. $O_{on}$ and $O_{off}$ represent the WDR5-released and WDR5-captured substates, respectively. The numbers of independently reconstituted nanopores were 3, 4, and 4, for single-channel electrical traces acquired at 90 nM WDR5, 500 nM WDR5, and 800 nM WDR5, respectively.

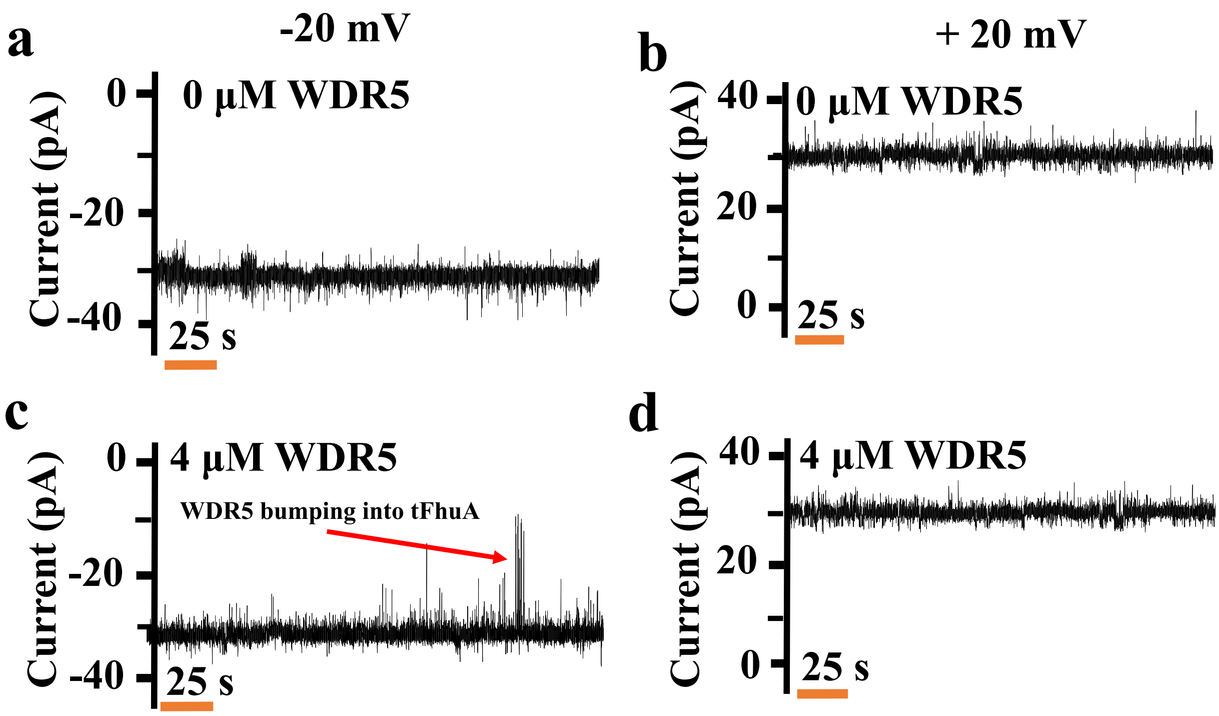


**Supplementary Figure 3. Extended-time single-channel electrical traces acquired with the unmodified nanopore, tFhuA without the MLL4$_{Win}$ ligand.** These are negative-control single-channel electrical recordings with the unmodified tFhuA nanopore, without the MLL4$_{win}$ ligand. **(a)** A representative single-channel electrical trace acquired with a tFhuA nanopore[2, 3] in the absence of WDR5. The applied transmembrane potential was −20 mV. **(b)** A representative single-channel electrical trace as in (a), but recorded at an applied transmembrane potential of +20 mV. **(c)** A representative single-channel electrical trace of tFhuA in the presence of 4 µM WDR5 added to the *cis* compartment. The applied transmembrane potential was −20 mV. Very rare and brief current blockades were noted. These current blockades were likely brought about by collisions of WDR5 with the *cis* opening of the tFhuA nanopore. (**d**) A representative single-channel electrical trace as in (c), but recorded at an applied transmembrane potential of +20 mV. All single-channel electrical traces spanned 225 s. They were low-pass filtered using an 8-pole Bessel filter at a frequency of 100 Hz. These negative-control recordings were replicated in n = 4 distinct experiments.

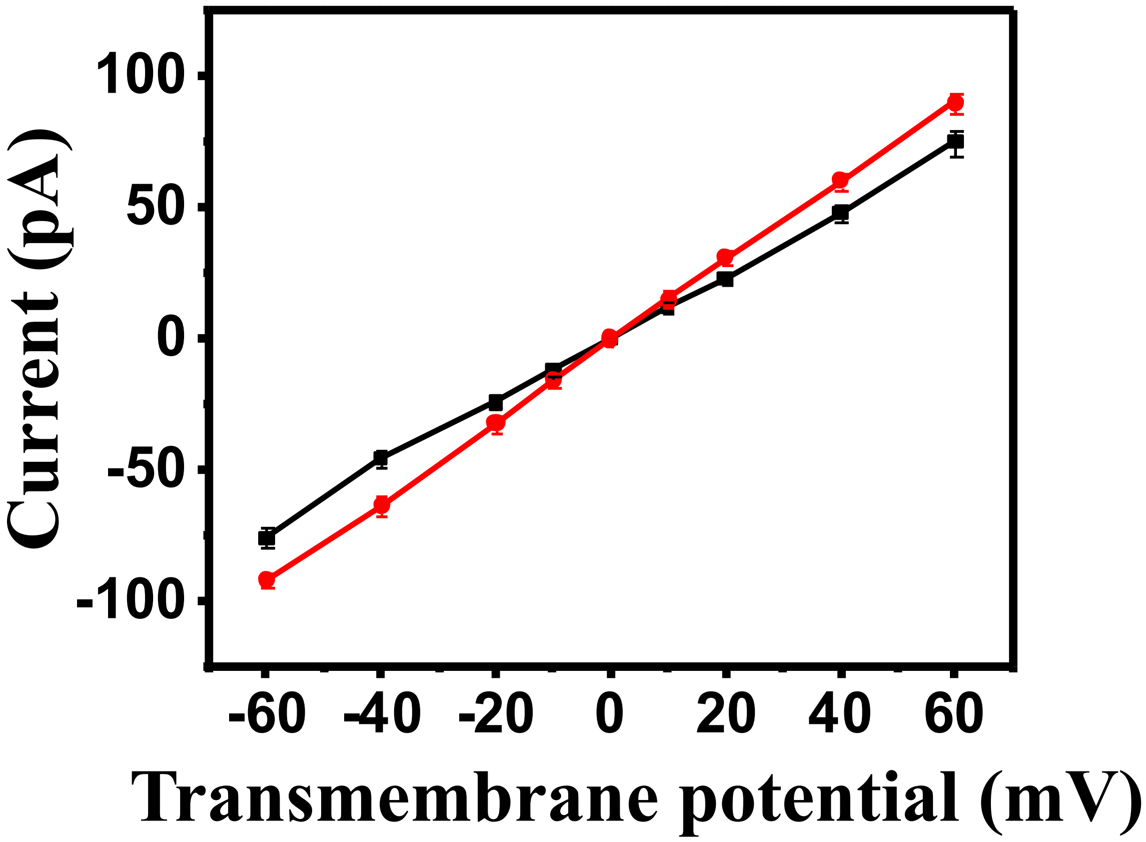


**Supplementary Figure 4. The *I*/*V* curves of tFhuA and MLL4$_{Win}$tFhuA nanopores.** These plots indicate the open-state current as a function of the transmembrane potential for the unmodified tFhuA nanopore (*red circles*) and MLL4$_{win}$tFhuA nanopore (*black squares*). Data points represent mean ± s.d. obtained from n = 3 independent nanopore experiments for each case. The other experimental conditions were the same as those stated in Methods. Source data are provided as a Source Data file.

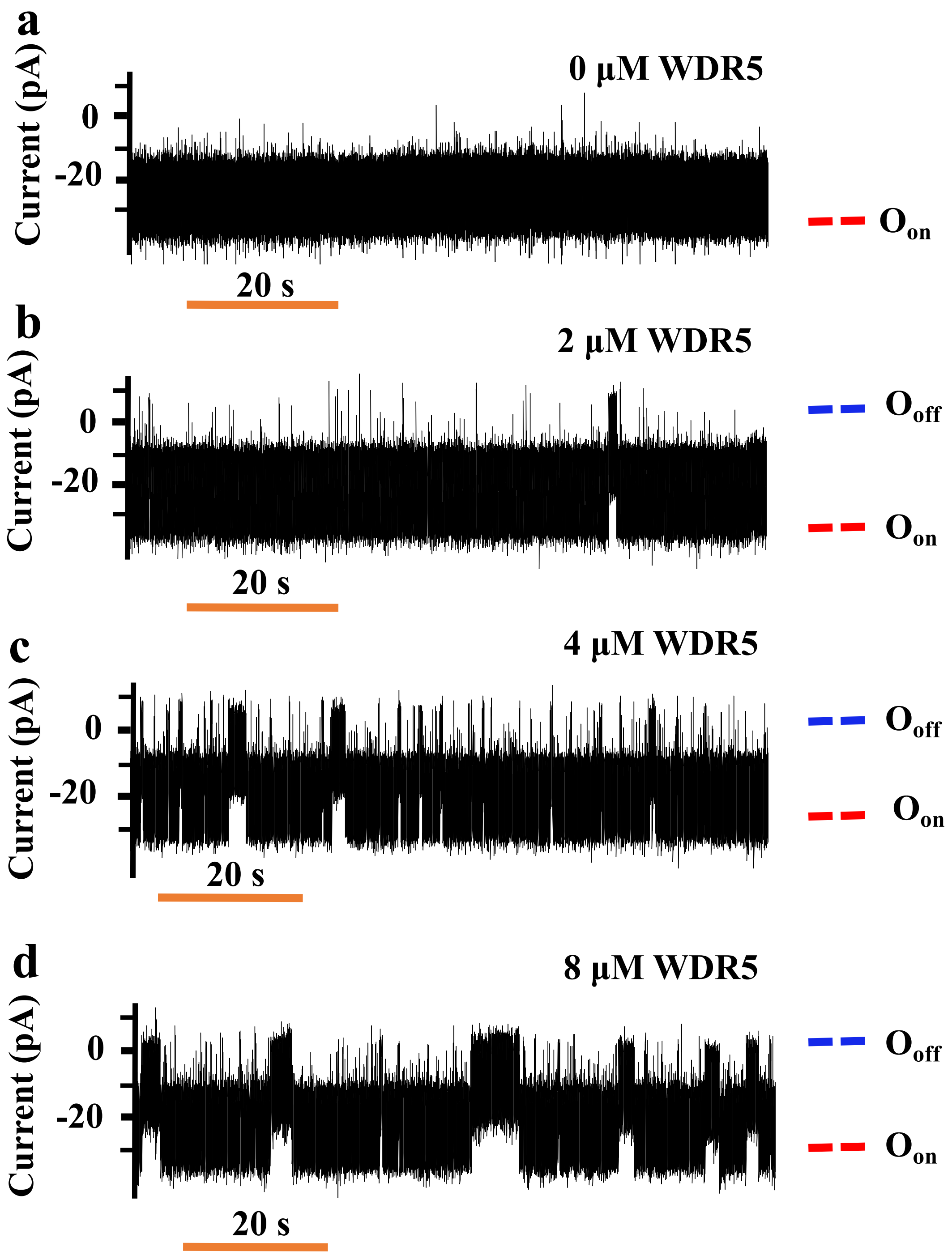


**Supplementary Figure 5. Unprocessed representative single-channel electrical traces of MLL4$_{Win}$tFhuA when WDR5 was added to the *cis* compartment.** These are raw single-channel electrical traces of MLL4$_{Win}$tFhuA measured at various [WDR5] values. These traces were low-pass Bessel filtered at a frequency of 10 kHz. **(a)** A representative single-channel electrical trace was recorded with a single MLL4$_{Win}$tFhuA at 0 µM WDR5. **(b)** The same trace as in (a), but in the presence of 2 µM WDR5. **(c)** The same trace as in (a), but in the presence of 4 µM WDR5. **(d)** The same trace as in (a), but in the presence of 8 µM WDR5. $O_{on}$ and $O_{off}$ represent the WDR5-released and WDR5-captured substates, respectively.

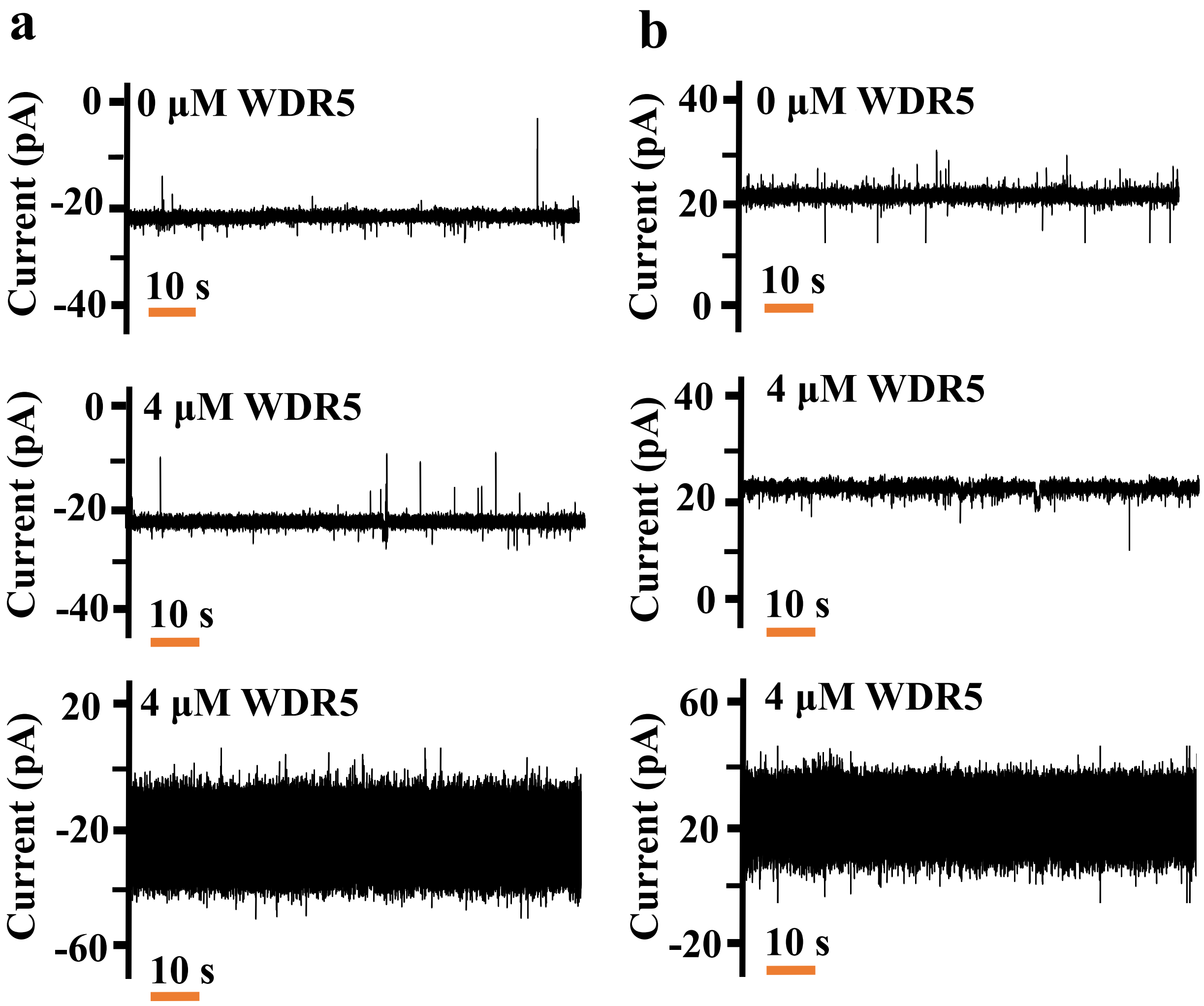


**Supplementary Figure 6. The MLL4$_{Win}$tFhuA nanopore with no WDR5-capture events when WDR5 was added to the *trans* compartment**. These are negative-control single-channel electrical recordings with MLL4$_{Win}$tFhuA when WDR5 was added to the *trans* compartment. In this case, transient interactions of the attached MLL4$_{Win}$ peptide with WDR5 were not observed. The single-channel electrical traces were low-pass filtered at 100 Hz using an eight-pole Bessel filter unless stated otherwise. **(a)** Demonstrative single-channel electrical traces of the MLL4$_{Win}$tFhuA at -20 mV with 0 µM WDR5 and 4 µM WDR5. The bottom panel is the same as the middle panel, but filtered using a low-pass 8-pole Bessel filter at a frequency of 10 kHz. **(b)** Demonstrative single-channel electrical traces of the MLL4$_{Win}$tFhuA at +20 mV with 0 µM WDR5 and 4 µM WDR5. The bottom panel is the same as the middle panel, but filtered using a low-pass 8-pole Bessel filter at a frequency of 10 kHz. These single-channel electrical signatures were replicated in n = 3 independent experiments.

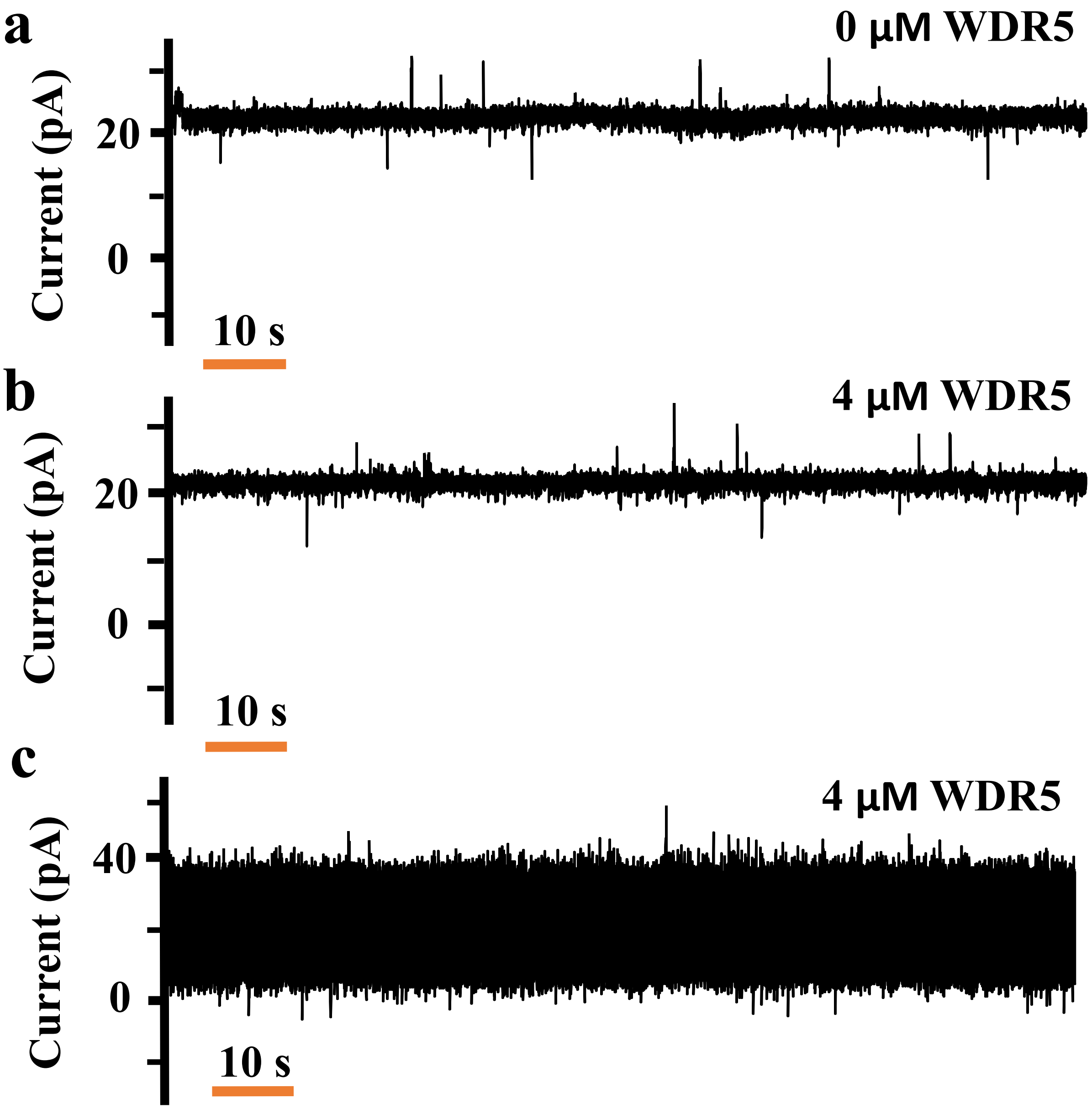


**Supplementary Figure 7. MLL4$_{Win}$tFhuA nanopore shows no WDR5-MLL4$_{Win}$ interactions at a positive transmembrane potential of + 20 mV**. These are positive-control single-channel electrical recordings with MLL4$_{Win}$tFhuA when WDR5 was added to the *cis* compartment and acquired at a positive transmembrane potential. **(a)** A demonstrative single-channel electrical trace of MLL4$_{Win}$tFhuA without WDR5. **(b)** MLL4$_{Win}$tFhuA with 4 µM WDR5 added to the *cis* compartment. **(c)** The same single-channel electrical trace as in panel (b), but filtered at a frequency of 10 kHz using a low-pass 8-pole Bessel filter. In traces (a) and (b), the single-channel electrical traces were low-pass filtered at 100 Hz using an 8-pole Bessel filter. These single-channel electrical signatures were replicated in n = 3 independent experiments.

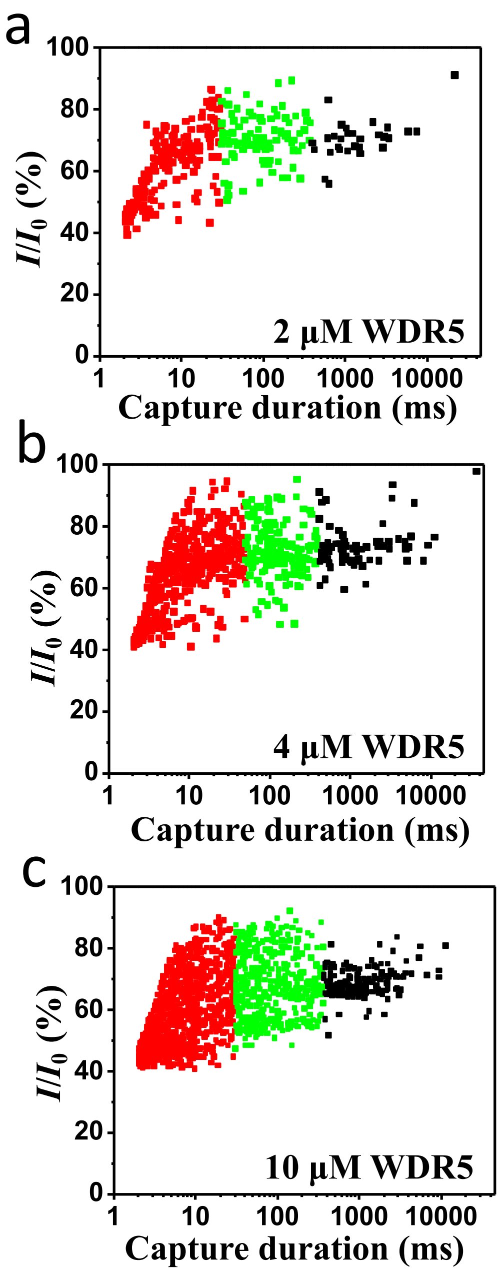


**Supplementary Figure 8. Distribution of WDR5-produced current blockades as a function of WDR5 capture duration.** Here, $I_0$ and $I$ denote the single-channel current of the WDR5-released substate of MLL4$_{\mathrm{Win}}$tFhuA and the amplitude of WDR5-produced current blockade, respectively. Semilogarithmic scatter plot of $I/I_0$ as a function of WDR5 capture duration probed by MLL4$_{\mathrm{Win}}$tFhuA in the presence of 2 µM WDR5 **(a)**, 4 µM WDR5 **(b)**, and 10 µM WDR5 **(c).** The short-, medium-, and long-lived events are represented in red, green, and black, respectively. The applied transmembrane potential was −20 mV. For the analysis of binding events, single-channel electrical traces were low-pass filtered using an eight-pole Bessel filter at a frequency of 1 kHz. Data was extracted from 20 minute-long single-channel electrical recordings. Source data are provided as a Source Data file.

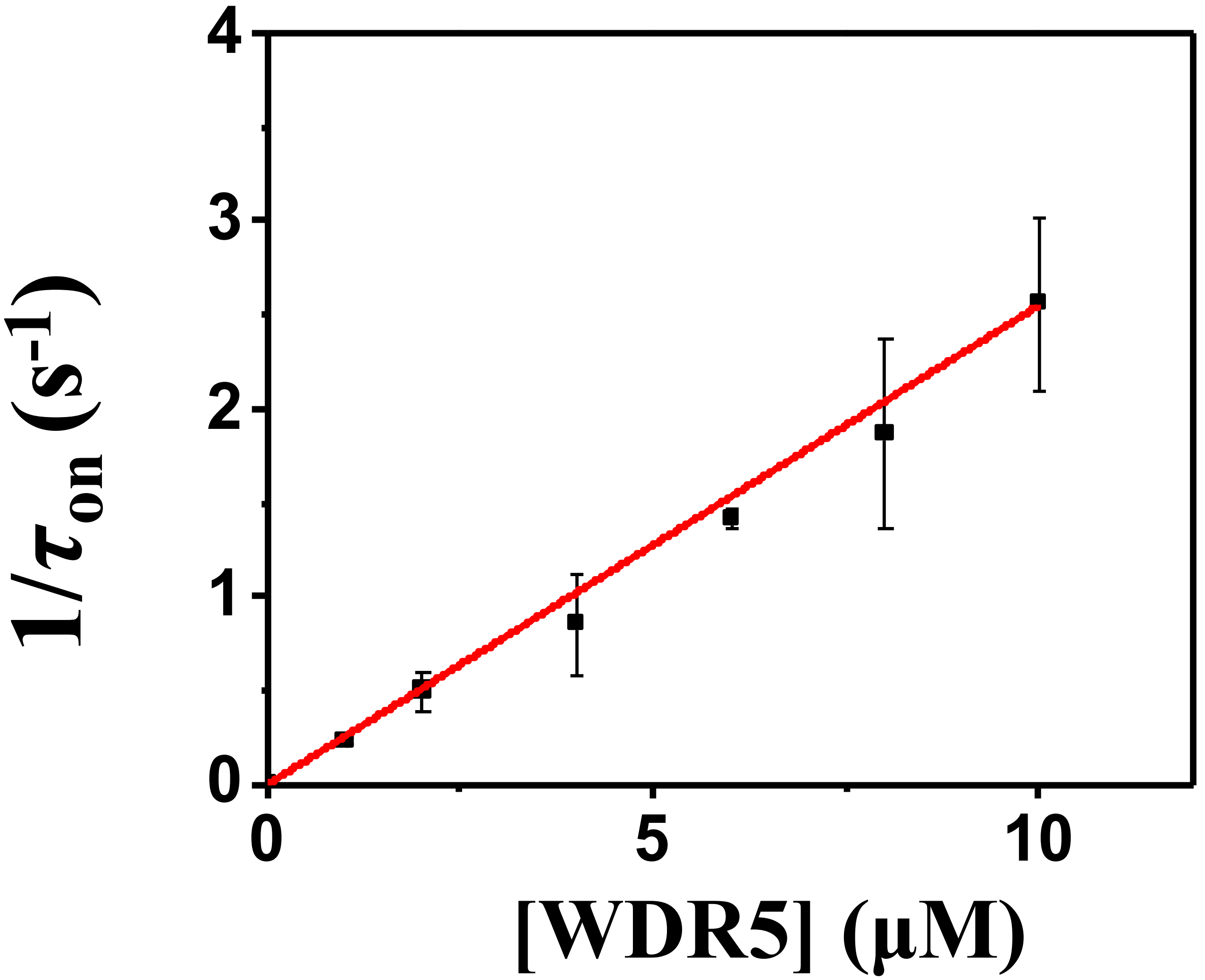


**Supplementary Figure 9. The overall kinetic rate constant of association of WDR5 captures.** Linear graph presenting the dependence of average $1/\tau_{on}$ on the WDR5 concentration, [WDR5]. The average $1/\tau_{on}$ accounts for all event types equally. The slope of the linear fit of $1/\tau_{on}$ versus [WDR5] is the association rate constant, $k_{on}$, because $k_{on} = 1/(\tau_{on}[\mathrm{WDR5}])$. Here, $k_{on} = (2.54 \pm 0.95) \times 10^5\ \mathrm{M}^{-1}\mathrm{s}^{-1}$. Data points represent mean ± s.d. obtained from n = 3 independent nanopore experiments. Source data are provided as a Source Data file.

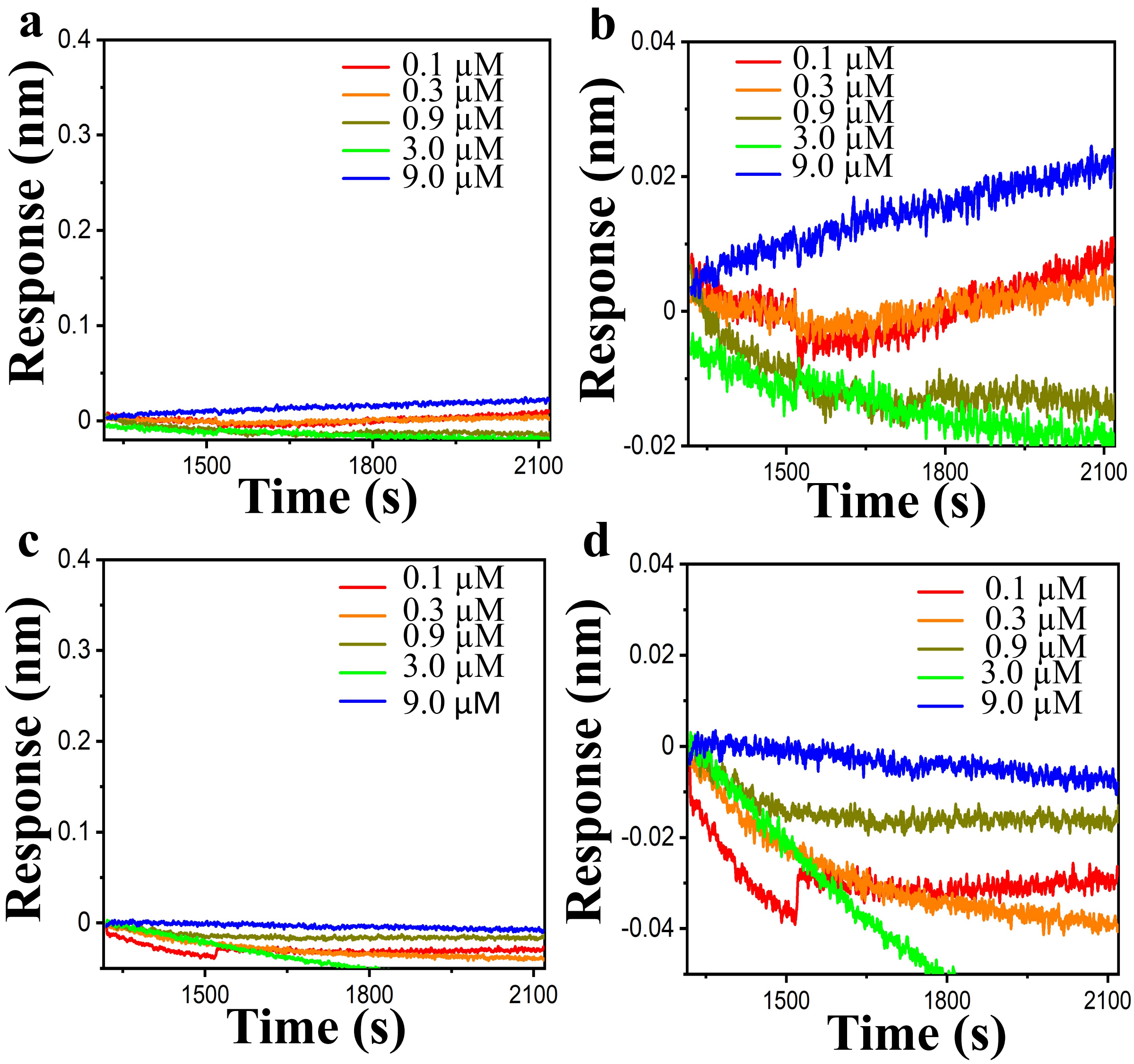


**Supplementary Figure 10. BLI sensorgrams of the peptide adaptor interacting with WDR5.** These are positive-control experiments with the peptide adaptor using biolayer interferometry (BLI) sensorgrams. A 3-fold serial dilution of WDR5 ranging from 0.1 µM to 9 µM was used to obtain individual binding curves. **(a)** BLI sensorgrams that show no response following the interaction between the peptide adaptor and WDR5. 5 nM biotin-tagged peptide adaptor was loaded onto streptavidin (SA) sensors for 5 minutes. This peptide was $_{Ac}$MGDRGPEFELGT(GGS)$_2$K-$_{Biotinyl}$. Lys at the C terminus was used for covalent attachment of biotin to the C terminus. The peptide adaptor showed no interaction with WDR5. **(b)** The y-axis on panel (a) was reduced to show that only a signal drift occurs and there is no specific interaction with WDR5. **(c)** BLI sensorgrams that show no response following the interaction between the peptide adaptor and WDR5. Here, we loaded 15 nM biotinylated peptide adaptor onto the BLI sensor instead of our usual concentration of 5 nM. No peptide adaptor-WDR5 interaction was detectable. **(d)** The y-axis on panel (c) was reduced to show that only a signal drift occurs and there is no specific interaction with WDR5. Source data are provided as a Source Data file.

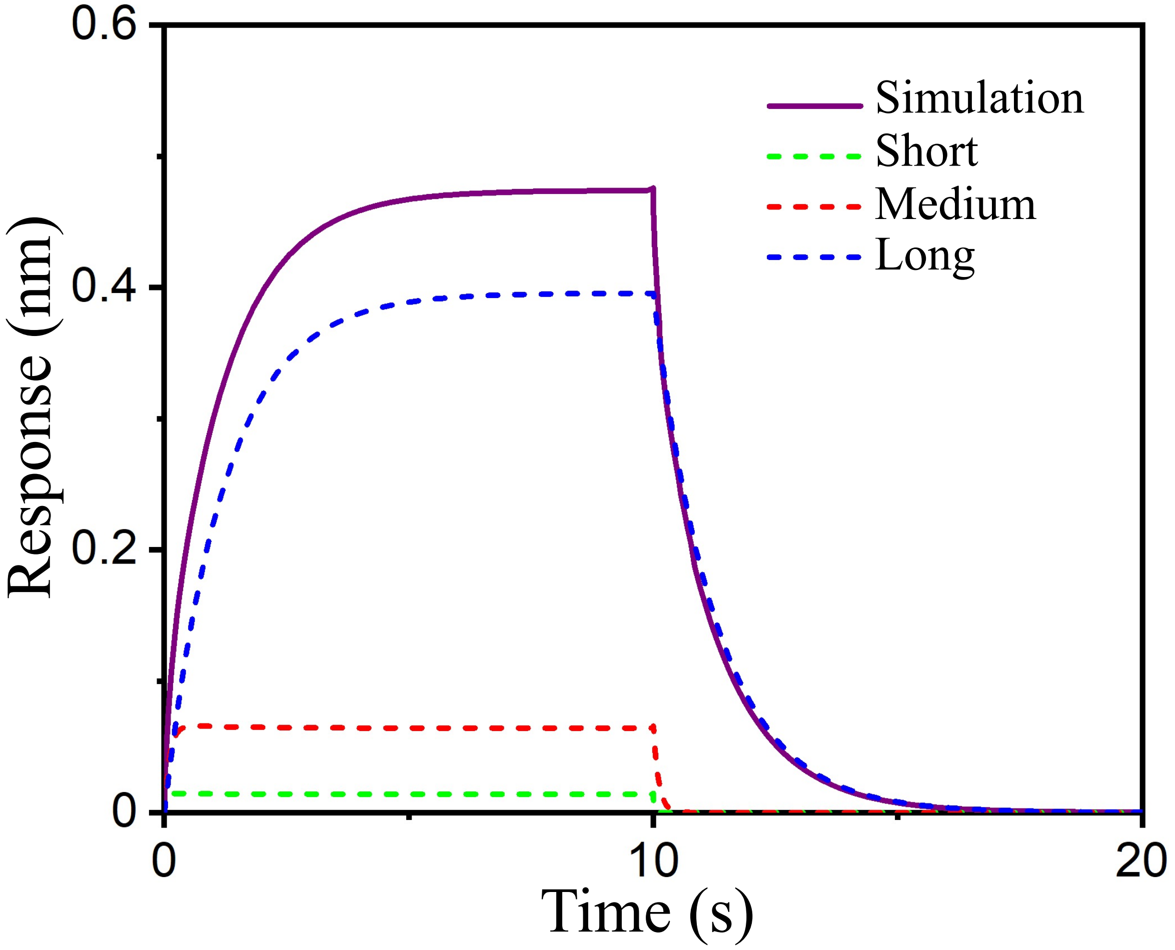


**Supplementary Figure 11. A simulation of the BLI response using results from single-channel electrical recordings.** This simulated BLI sensorgram (the continuous curve) corresponds to 0.9 μM WDR5. Dashed lines in green, red, and blue, indicate the contributions of short-, medium-, and long-lived MLL4$_{Win}$-WDR5 interactions recorded with our proteomic MLL4$_{Win}$tFhuA nanopore, respectively. This BLI sensorgram shows the association and dissociation curves for the three binding events, as well as those for the cumulative response.

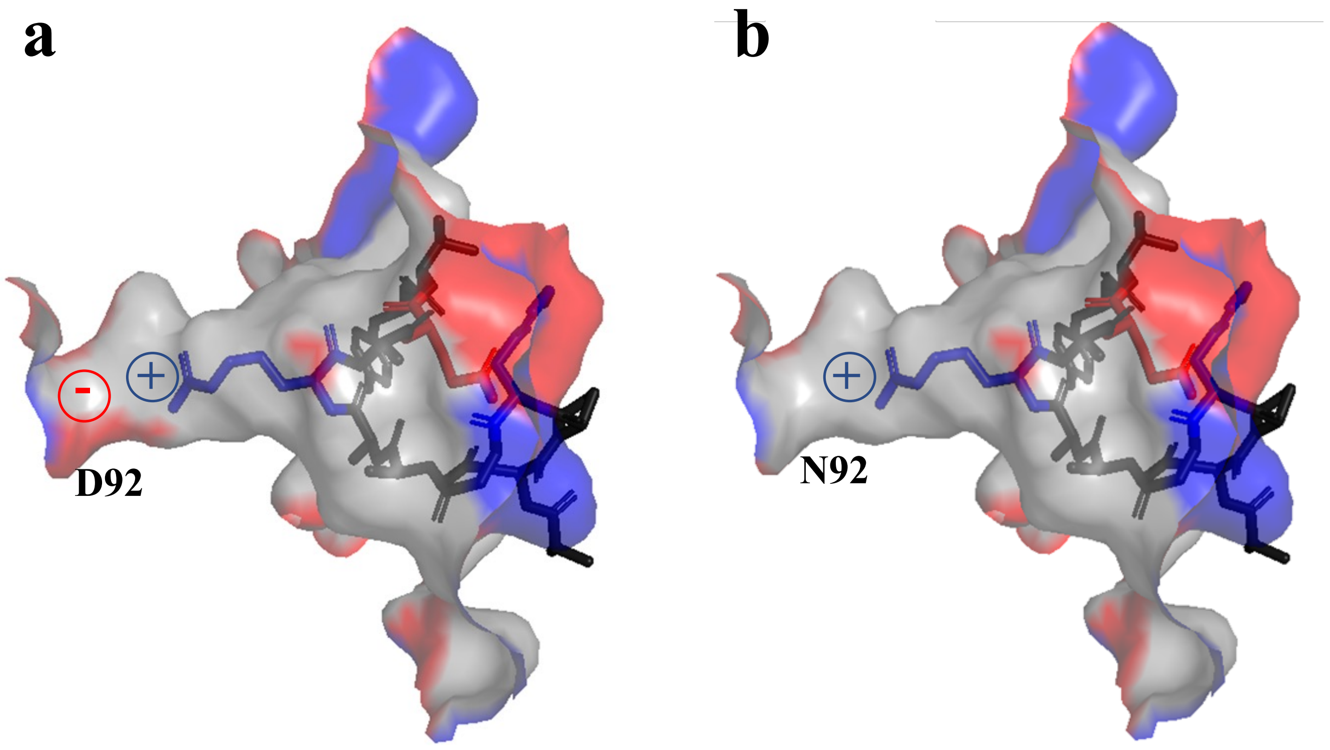


**Supplementary Figure 12. Cross-sectional view of the Win binding site of WDR5, which reveals the location of Asp-92 residue with respect to the highly conservative Arg-residue of MLL4$_{\mathbf{Win}}$.** The positively charged residues in WDR5 (light gray) and MLL4$_{win}$ (black) are labeled in blue. The negatively charged residues are labeled in red. WDR5 **(a)** and D92N$_{WDR5}$ **(b)** have a surface representation, whereas MLL4$_{win}$ has a stick representation. WDR5 has the negatively charged Asp-92 deep inside the binding cavity. Asp-92 assists in the electrostatic pulling of the positively charged Arg residue in position $P_0$ of MLL4$_{win}$ into the WDR5 cavity (Supplementary Tables 1-2). In D92N$_{WDR5}$, the negatively charged Asp-92 is replaced by a polar Asn-92, so the electrostatic attraction is removed. This mutation brings about an increase in the activation free energy barrier for the association process.

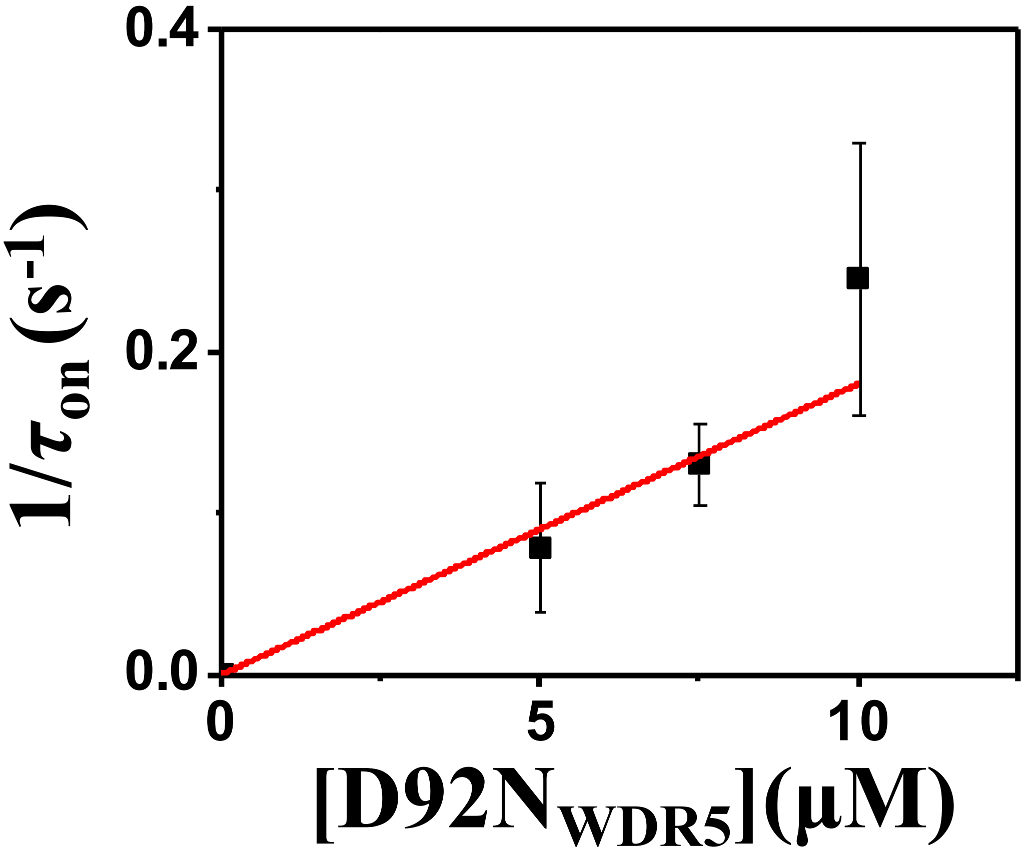


**Supplementary Figure 13. The overall rate constant of association of D92N$_{WDR5}$–MLL4$_{Win}$ interactions.** Linear graph presenting the dependence of the average $1/\tau_{on}$ on [D92N$_{WDR5}$]. The average $1/\tau_{on}$ accounts for all event types equally. The slope of the linear fit of $1/\tau_{on}$ versus [D92N$_{WDR5}$] is the association rate constant, $k_{on}$, because $k_{on} = 1/(\tau_{on}[D92N_{WDR5}])$. Here, $k_{on} = (3.1 \pm 0.2) \times 10^4$ $M^{-1}s^{-1}$. Data points represent mean ± s.d. obtained from n = 3 independent nanopore experiments. Source data are provided as a Source Data file.

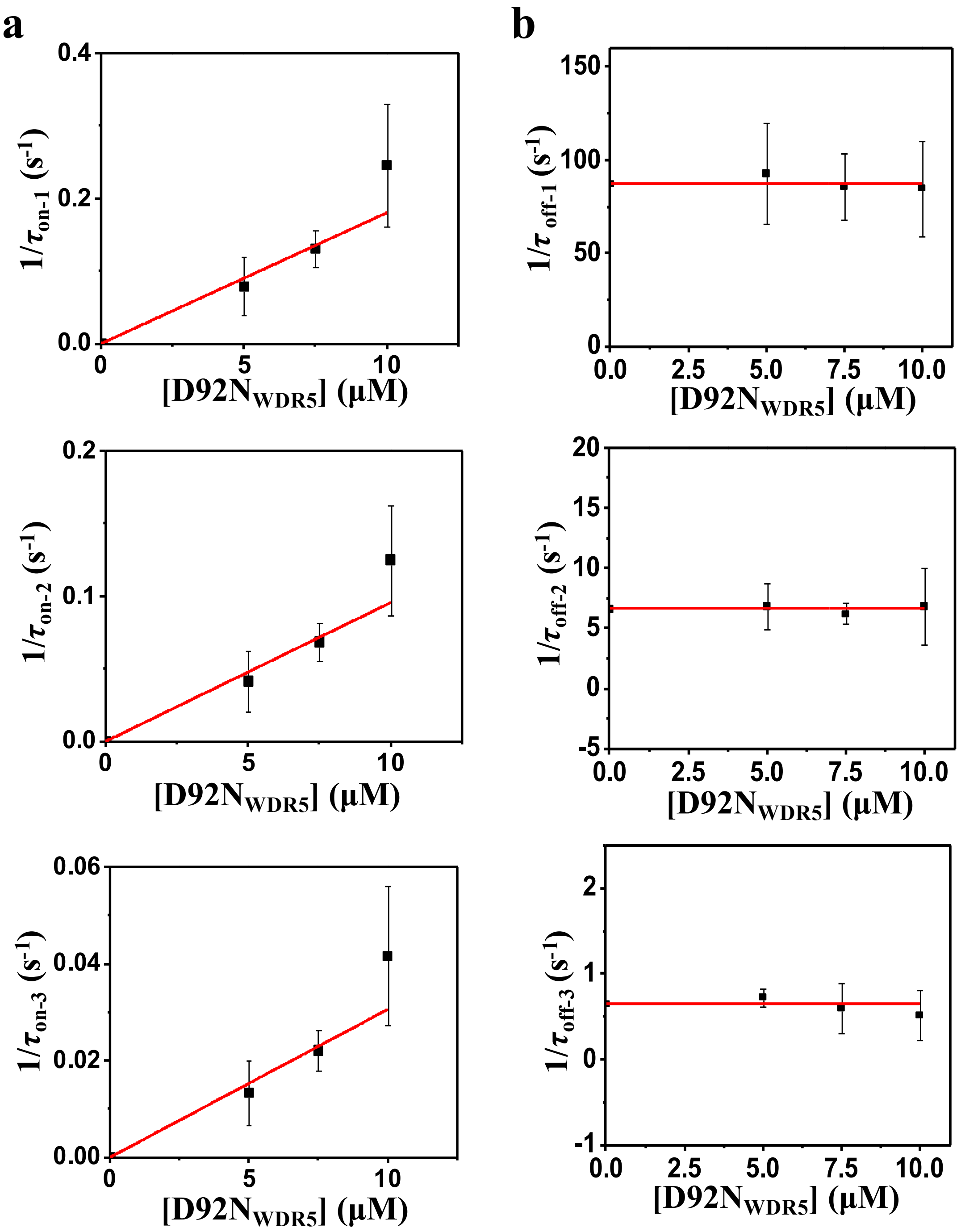


**Supplementary Figure 14. Dependences of $1/\tau_{on}$ and $1/\tau_{off}$ on [$D92N_{WDR5}$] for the three binding events. (a)** Dependences of $1/\tau_{on-i}$ on [$D92N_{WDR5}$]. $\tau_{on-1}$, $\tau_{on-2}$, and $\tau_{on-3}$ are the mean durations of time intervals between short-, medium-, and long-lived lived $D92N_{WDR5}$ captures, respectively. **(b)** Dependences of $1/\tau_{off-1}$ on [$D92N_{WDR5}$]. $\tau_{off-1}$, $\tau_{off-2}$, and $\tau_{off-3}$ are the mean durations of the short-, medium-, and long-lived lived $D92N_{WDR5}$ captures, respectively. Diagrams show that the mean $\tau_{off}$ duration of all three event types was independent of [$D92N_{WDR5}$]. Data points in all panels represent mean ± s.d. obtained from n = 3 distinct experiments. Source data are provided as a Source Data file.

**a**

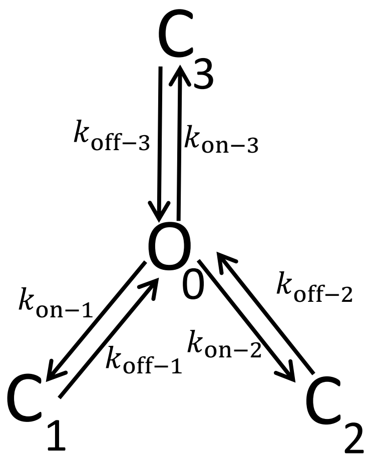


**b**

$$\begin{pmatrix} -k_{\mathbf{on-1}} - k_{\mathbf{on-2}} - k_{\mathbf{on-3}} & k_{\mathbf{on-1}} & k_{\mathbf{on-2}} & k_{\mathbf{on-3}} \\ k_{\text{off}-1} & -k_{\text{off}-1} & 0 & 0 \\ k_{\text{off}-2} & 0 & -k_{\text{off}-2} & 0 \\ k_{\text{off}-3} & 0 & 0 & -k_{\text{off}-3} \end{pmatrix}$$

**Supplementary Figure 15. The interconversion-independent kinetic model.** This model assumes no transitions between different capture substates, $C_1$, $C_2$, and $C_3$. **(a)** The schematic of the model. 'O' represents the release substate, while 'C's indicate the three different capture substates. Each substate is numbered 0, 1, 2, or 3. **(b)** Q-matrix[4-7] of the interconversion-independent four-substate kinetic model.

a

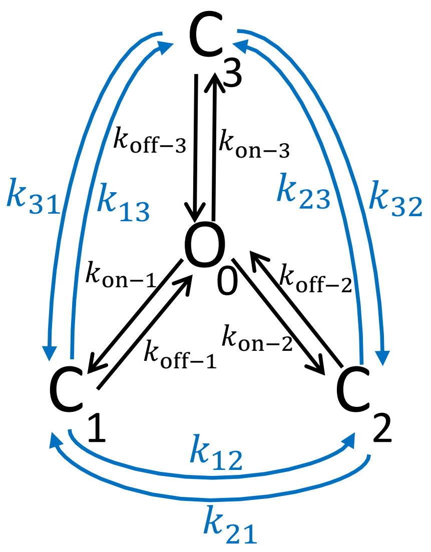


b

$$\begin{pmatrix} -k_{\mathbf{on-1}} - k_{\mathbf{on-2}} - k_{\mathbf{on-3}} & k_{\mathbf{on-1}} & k_{\mathbf{on-2}} & k_{\mathbf{on-3}} \\ k_{\mathrm{off-1}} & -k_{\mathrm{off-1}} - k_{12} - k_{13} & k_{12} & k_{13} \\ k_{\mathrm{off-2}} & k_{21} & -k_{\mathrm{off-2}} - k_{21} - k_{23} & k_{23} \\ k_{\mathrm{off-3}} & k_{31} & k_{32} & -k_{\mathrm{off-3}} - k_{31} - k_{32} \end{pmatrix}$$

**Supplementary Figure 16. The interconversion-dependent kinetic model.** This model assumes transitions between different capture substates, $C_1$, $C_2$, and $C_3$. **(a)** The schematic of the model. 'O' represents the release substate, while 'C's indicate the three different capture substates. Each substate is numbered 0, 1, 2, or 3. The interconversion rate constants show transitions between different substates. Here, the first digit denotes the initial state and the second digit indicates the final state. Transitions from capture substates to release substates and vice-versa are marked in black. Transitions between capture substates are marked in blue. **(b)** Q-matrix[4-7] of the interconversion-dependent four-substate kinetic model.

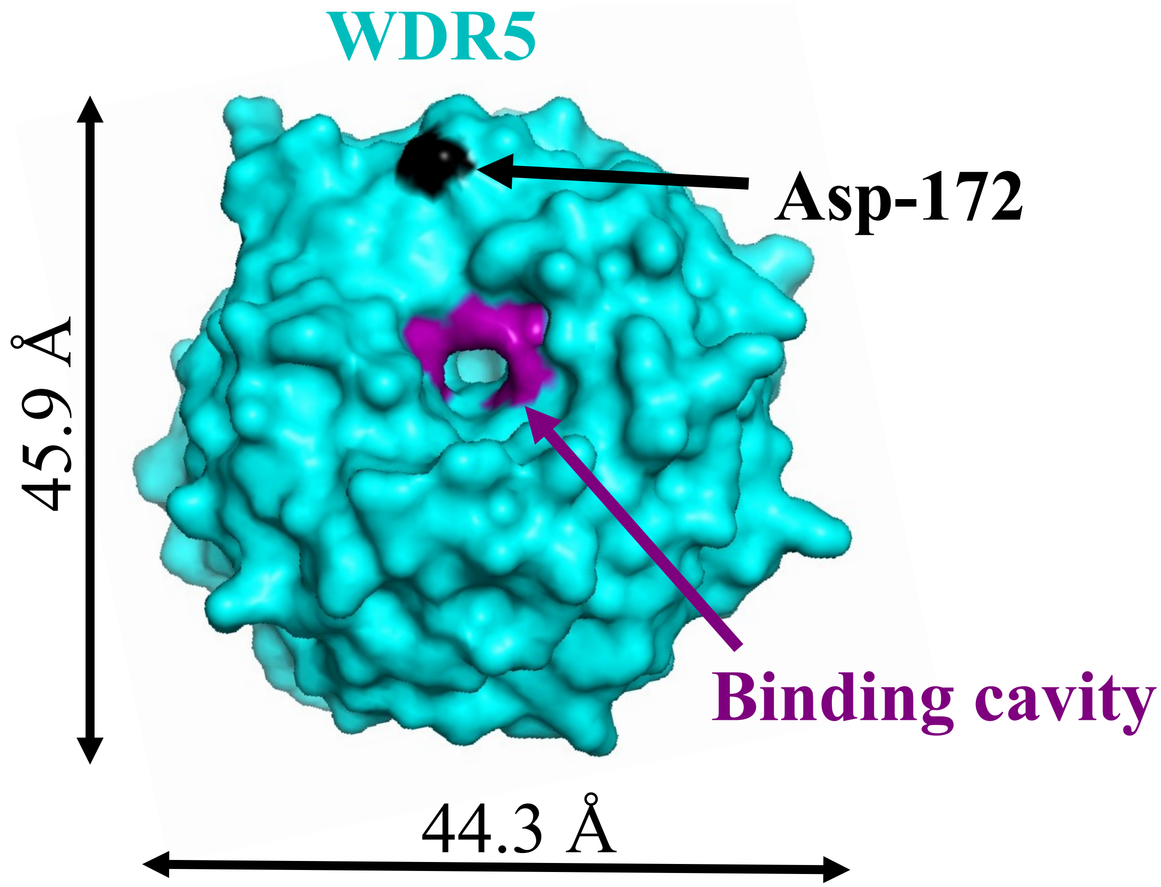


**Supplementary Figure 17. Top view of WDR5 showing the surface location of Asp-172 in the proximity of the Win binding site.** This model was developed using Protein Data Bank file 4ERZ.pdb (MLL4$_{Win}$–WDR5).[1] A top-view cartoon of WDR5 (in cyan) shows the binding cavity. Phe-133, Cys-261, and Ser-91 are colored in magenta. Asp-172 is labeled in black.

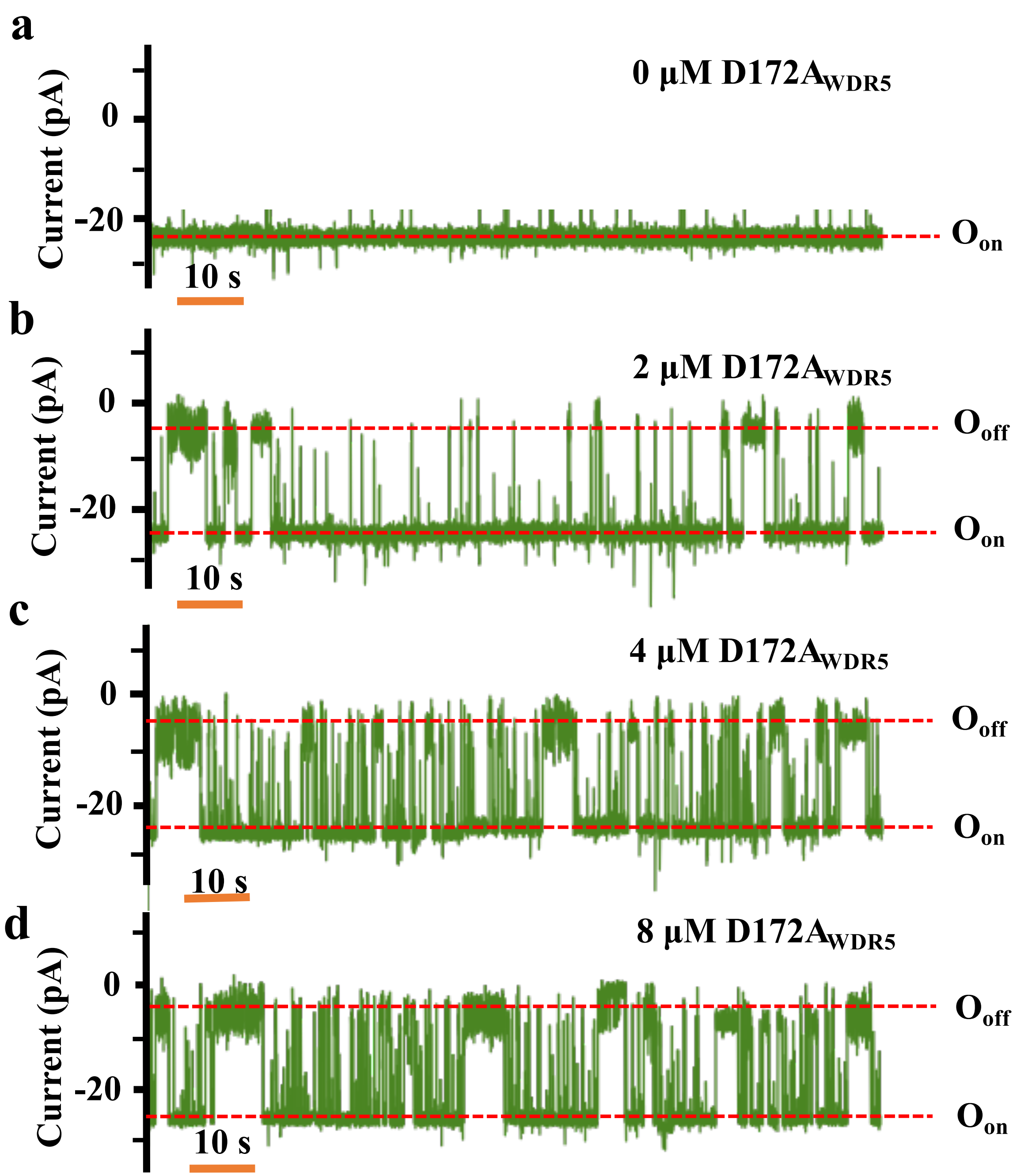


**Supplementary Figure 18. Positive-control single-channel electrical signatures of the D172A$_{WDR5}$-capture and D172A$_{WDR5}$-release events. (a)** A single-channel electrical trace of MLL4$_{Win}$tFhuA. **(b)** Reversible D172A$_{WDR5}$ captures were observed through current transitions between the $O_{on}$ and $O_{off}$ substates when 2 µM D172A$_{WDR5}$ was added to the *cis* compartment. **(c)** MLL4$_{Win}$tFhuA with 4 µM D172A$_{WDR5}$. **(d)** MLL4$_{Win}$tFhuA with 8 µM D172A$_{WDR5}$. In traces (a) - (d), the transmembrane potential was −20 mV. Single-channel electrical traces were low-pass filtered at a frequency of 100 Hz using an 8-pole Bessel filter. These single-channel electrical signatures were replicated in n = 3 independent experiments.

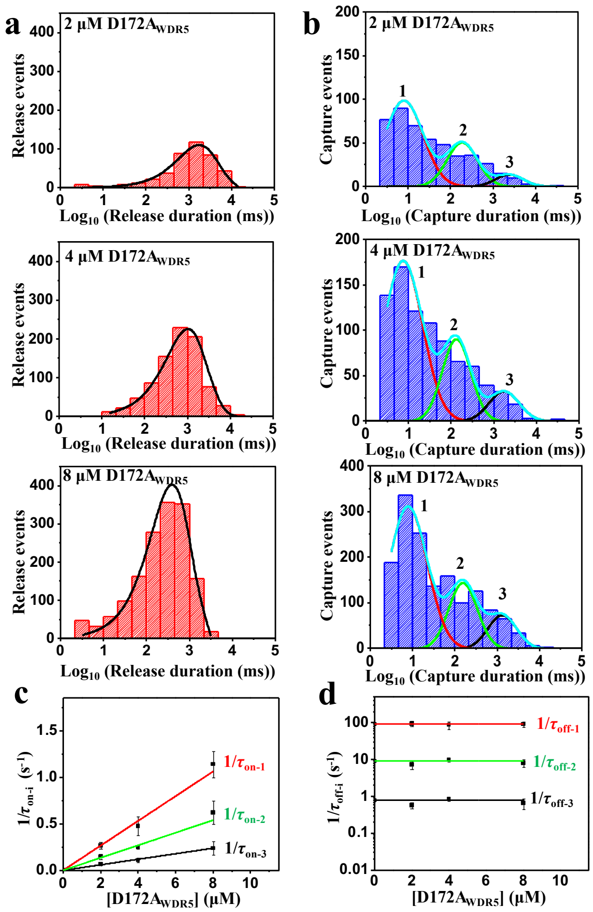


**Supplementary Figure 19. D172A$_{WDR5}$-captured and D172A$_{WDR5}$-released event histograms and kinetic graphs. (a)** Event histograms of the D172A$_{WDR5}$-released durations ($\tau_{on}$). The $\tau_{on}$ values (mean ± s.e.m.) were 1.8 ± 0.2 s (number of events: $N = 426$), 1.2 ± 0.3 s ($N = 866$), and 0.47 ± 0.07 s ($N = 1556$) at 2, 4, and 8 μM D172A$_{WDR5}$, respectively. **(b)** Event histograms of the D172A$_{WDR5}$-captured durations ($\tau_{off}$). The cumulative fit of each histogram is represented by a cyan curve. The red, green, and black curves indicate fit components $\tau_{off-1}$, $\tau_{off-2}$, and $\tau_{off-3}$ for short-, medium-, and long-lived D172A$_{WDR5}$ captures, respectively. For 2 μM D172A$_{WDR5}$, these durations (mean ± s.e.m.) were 0.008 ± 0.001 s, 0.17 ± 0.002 s, and 2.2 ± 0.2 s, respectively (number of events: $N = 465$). For 4 μM D172A$_{WDR5}$, these durations (mean ± s.e.m.) were 0.008 ± 0.001 s, 0.11 ± 0.02 s, and 1.7 ± 0.1 s, respectively ($N = 844$). For 8 μM D172A$_{WDR5}$, these durations (mean ± s.e.m.) were 0.010 ± 0.002 s, 0.12 ± 0.01 s, and 1.2 ± 0.2 s, respectively ($N = 1{,}487$). Data was extracted from 20-minute recordings. **(c)** Dependence of $1/\tau_{on-i}$ on [D172A$_{WDR5}$], where i = 1, 2, and 3 for short-, medium-, and long-lived events, respectively. Here, $\tau_{on-i}$ with i = 1, 2, and 3, are the mean durations of intervals between short-, medium-, and long-lived D172A$_{WDR5}$ captures, respectively. The slopes of the linear fits of $1/\tau_{on-i}$ versus [D172A$_{WDR5}$] are association rate constants, $k_{on-i}$, because $k_{on-i} = 1/(\tau_{on-i}[\text{D172A}_{WDR5}])$. **(d)** Dependence of $1/\tau_{off-i}$ on [D172A$_{WDR5}$], where i = 1, 2, and 3, respectively. Here, $\tau_{off-i}$ with i = 1, 2, and 3, are the mean durations of short-, medium-, and long-lived D172A$_{WDR5}$ captures, respectively. The horizontal lines are average fits of the ($1/\tau_{off-i}$) data points, representing dissociation rate constants, $k_{off-i}$, because $k_{off-i} = 1/\tau_{off-i}$. In all panels, data points represent mean ± s.d. obtained from n = 3 distinct experiments. Source data are provided as a Source Data file.

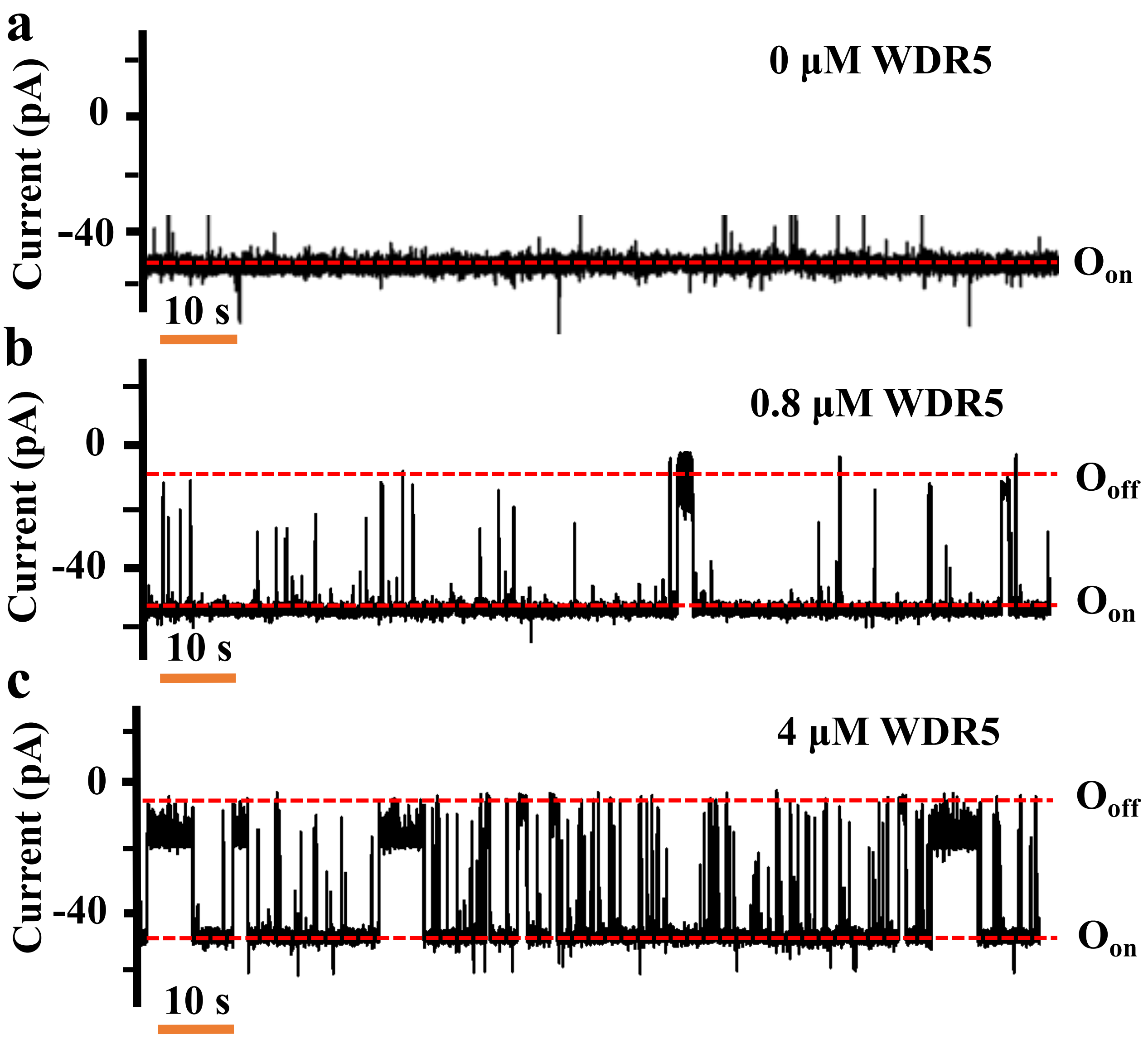


**Supplementary Figure 20. Real-time observation of the three binding events of MLL4$_{Win}$-WDR5 interactions at a transmembrane potential of -40 mV.** $O_{on}$ represents the WDR5-released open substate, whereas $O_{off}$ indicates the WDR5-captured closed substate. **(a)** A single-channel electrical trace of MLL4$_{Win}$tFhuA. **(b)** Reversible WDR5 captures were observed through current transitions between the $O_{on}$ and $O_{off}$ substates when 0.8 µM WDR5 was added to the *cis* compartment. **(c)** MLL4$_{Win}$tFhuA with 4 µM WDR5. Single-channel electrical traces were low-pass filtered at 100 Hz using an 8-pole Bessel filter. These single-channel electrical signatures were replicated in three independent experiments.

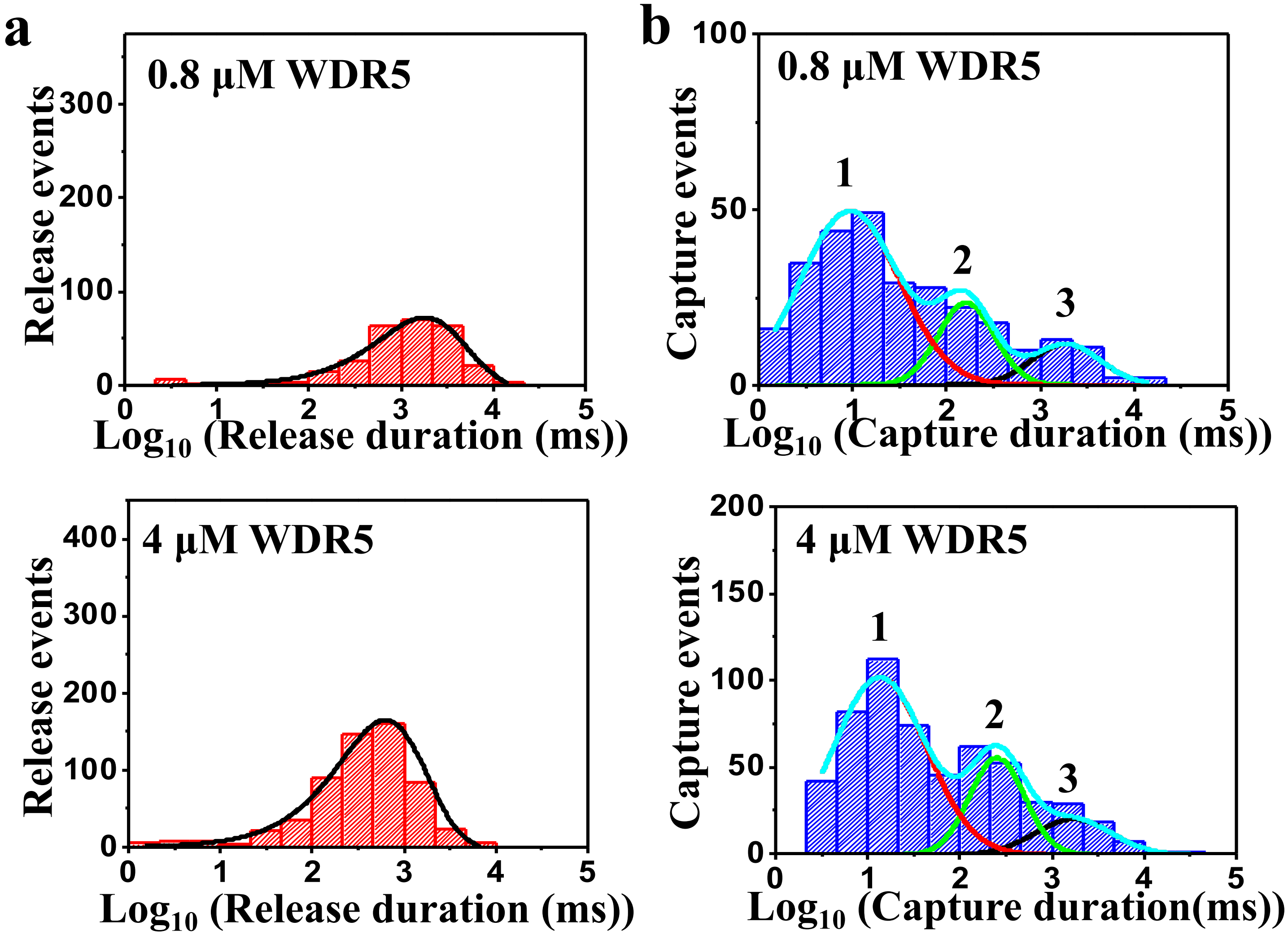


**Supplementary Figure 21. Event histograms acquired at a transmembrane potential of -40 mV. (a)** Semilogarithmic event histograms of the WDR5-released durations ($\tau_{on}$) at various [WDR5] values. The $\tau_{on}$ durations (mean ± s.e.m.) were 1.96 ± 0.26 s (number of events: $N$ = 280) and 0.718 ± 0.21 s ($N$ = 697) at 0.8 and 4 μM WDR5, respectively. **(b)** Semilogarithmic event histograms of the WDR5-captured durations ($\tau_{off}$) at various [WDR5] values. The cumulative fit of each histogram is represented by a cyan curve. The red, green, and black curves indicate fit components $\tau_{off\text{-}1}$, $\tau_{off\text{-}2}$, and $\tau_{off\text{-}3}$ for the short-, medium-, and long-lived WDR5 captures, respectively. For 0.8 μM WDR5, these durations (mean ± s.e.m.) were 0.009 ± 0.001 s, 0.117 ± 0.002 s, and 1.99 ± 0.004 s, respectively (number of events: $N$ = 279). For 4 μM WDR5, the three $\tau_{off}$ times (mean ± s.e.m.) were 0.014 ± 0.002 s, 0.219 ± 0.005 s, and 1.98 ± 0.008 s, respectively ($N$ = 588). Source data are provided as a Source Data file.

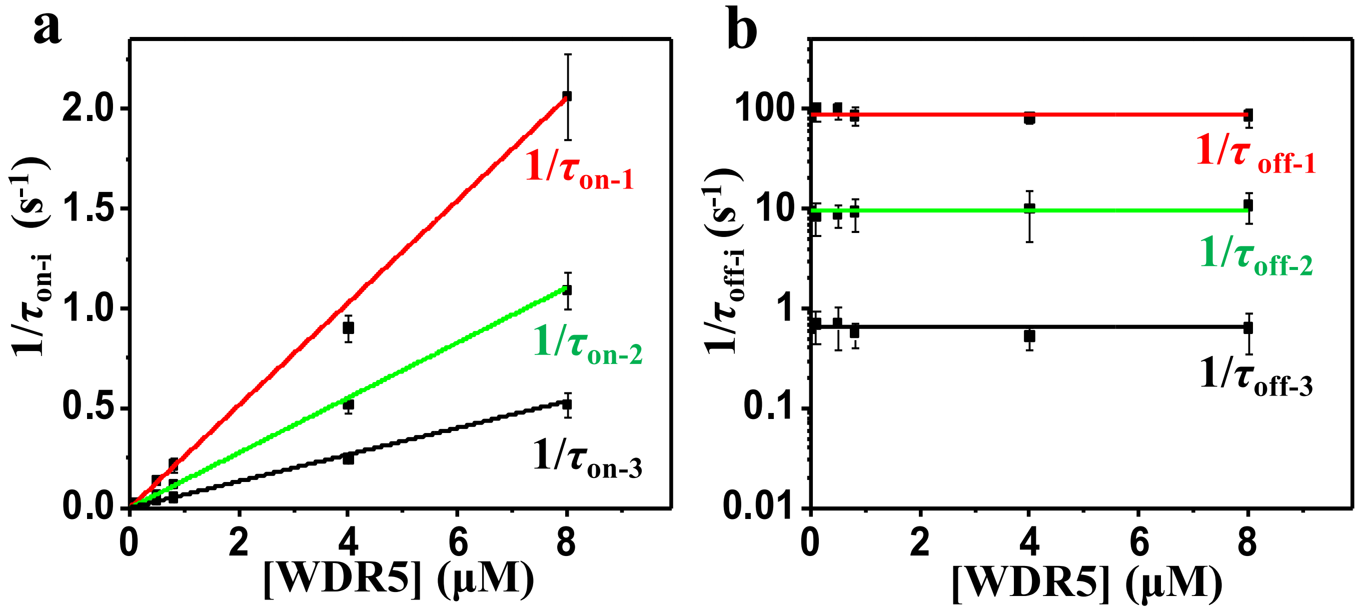


**Supplementary Figure 22. Dependences of $1/\tau_{on}$ and $1/\tau_{off}$ on [WDR5] for the three binding events at a transmembrane potential of -40 mV. (a)** The dependence of $1/\tau_{on\text{-}i}$ on [WDR5], where i = 1, 2, and 3. Here, $\tau_{on\text{-}i}$, with i = 1, 2, and 3, are the mean durations of intervals between short-, medium-, and long-lived WDR5-captured events, respectively. The slope of the linear fit of $1/\tau_{on\text{-}i}$ versus [WDR5] is the association rate constant, $k_{on\text{-}i}$, because $k_{on\text{-}i}= 1/(\tau_{on\text{-}i}[\text{WDR5}])$. **(b)** The dependence of $1/\tau_{off\text{-}i}$ on [WDR5], where i = 1, 2, and 3. Here, $\tau_{off\text{-}i}$, with i = 1, 2, and 3, are the mean durations of short-, medium-, and long-lived WDR5 captures, respectively. The horizontal line is an average fit of the ($1/\tau_{off\text{-}i}$) data points, representing the dissociation rate constant, $k_{off\text{-}i}$, because $k_{off\text{-}i} = 1/\tau_{off\text{-}i}$. In all panels, data points represent mean ± s.d. obtained from n = 3 distinct experiments. Source data are provided as a Source Data file.

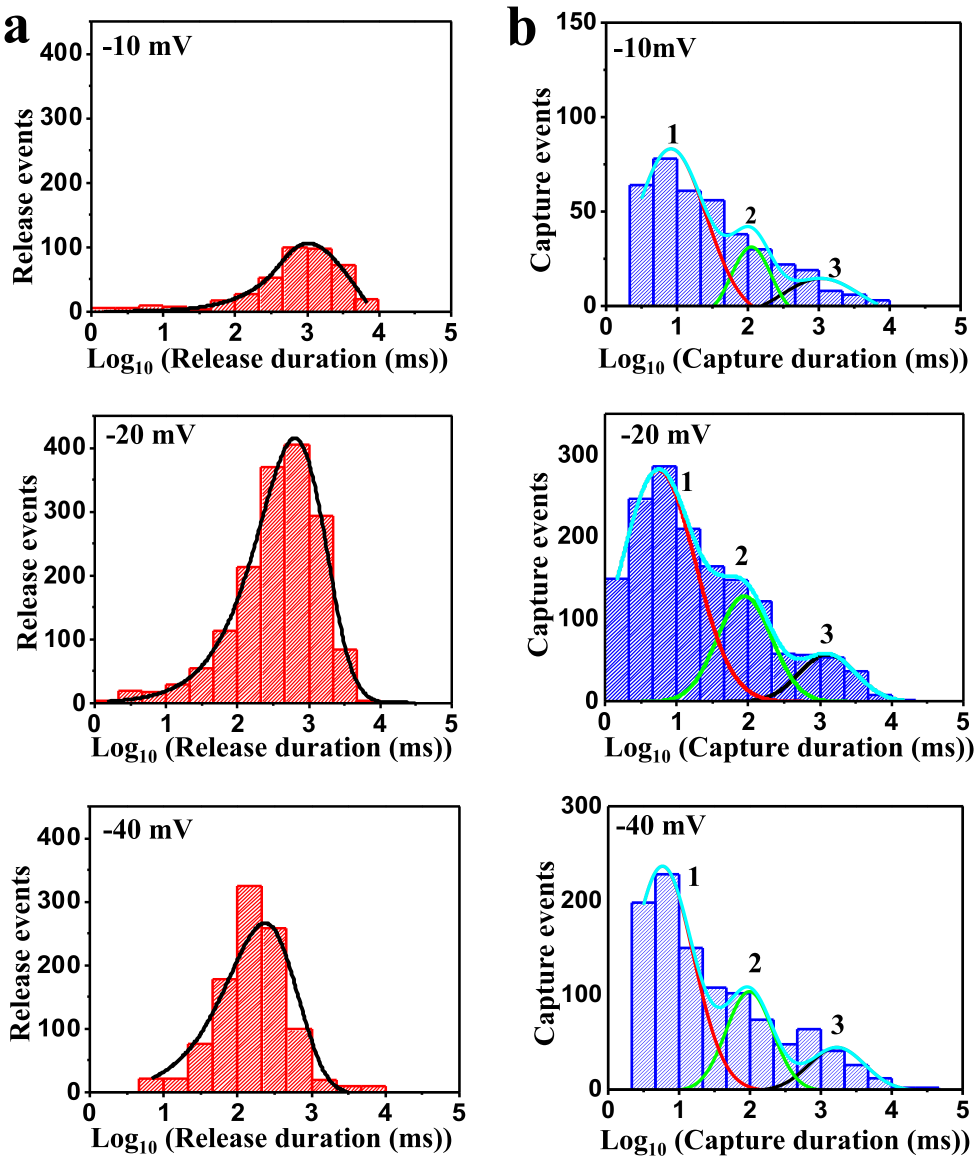


**Supplementary Figure 23. Event histograms acquired at various transmembrane potentials, Δ*U*. (a)** Semilogarithmic event histograms of the WDR5-released durations ($\tau_{on}$) at various voltages. The $\tau_{on}$ durations (mean ± s.e.m.) were 0.85 ± 0.01 s (number of events: *N* = 397), 0.55 ± 0.09 s (*N* = 1608), and 0.25 ± 0.09 s (*N* = 1023) at -10 mV, -20 mV, and -40 mV, respectively. **(b)** Semilogarithmic event histograms of the WDR5-captured durations ($\tau_{off}$) at various voltages. The cumulative fit of each histogram is represented by a cyan curve. The red, green, and black curves indicate fit components $\tau_{off-1}$, $\tau_{off-2}$, and $\tau_{off-3}$ for the short-, medium-, and long-lived WDR5 captures, respectively. For −10 mV, these durations (mean ± s.e.m.) were 0.011 ± 0.001 s, 0.096 ± 0.010 s, and 1.2 ± 0.1 s, respectively (number of events: *N* = 385). For −20 mV, the three $\tau_{off}$ times (mean ± s.e.m.) were 0.008 ± 0.001 s, 0.083 ± 0.010 s, and 1.3 ± 0.1 s, respectively (*N* = 1,406). For −40 mV, these durations were (mean ± s.e.m.) 0.008 ± 0.002 s, 0.10 ± 0.01 s, and 1.9 ± 0.1 s, respectively (*N* = 1,030). Data was extracted from 20-minute recordings for −20 mV and 8-minute recordings for −10 mV and −40 mV. 8 μM [WDR5] was added to the *cis* compartment. Source data are provided as a Source Data file.

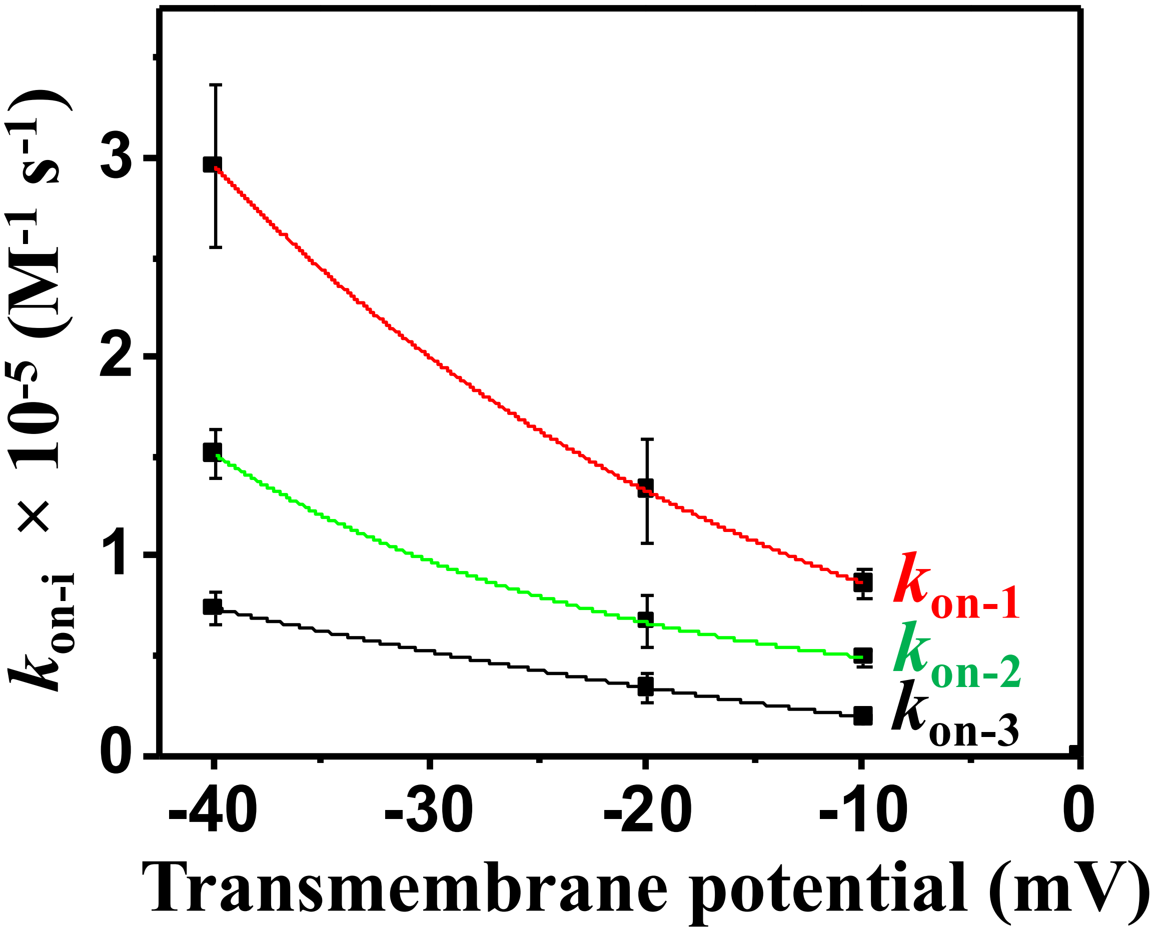


**Supplementary Figure 24. Voltage dependence of the association rate constants for the three binding events.** This plot shows the dependence of $k_{on-i}$ on the transmembrane potential, $\Delta U$. Here, i = 1, 2, and 3, denote short-, medium-, and long-lived binding events, respectively. $k_{on-i}$ ($\Delta U$) values can also be found in Supplementary Table 31. All values were acquired at 8 μM WDR5. All data points represent mean ± s.d. obtained from n = 3 distinct experiments. Source data are provided as a Source Data file.

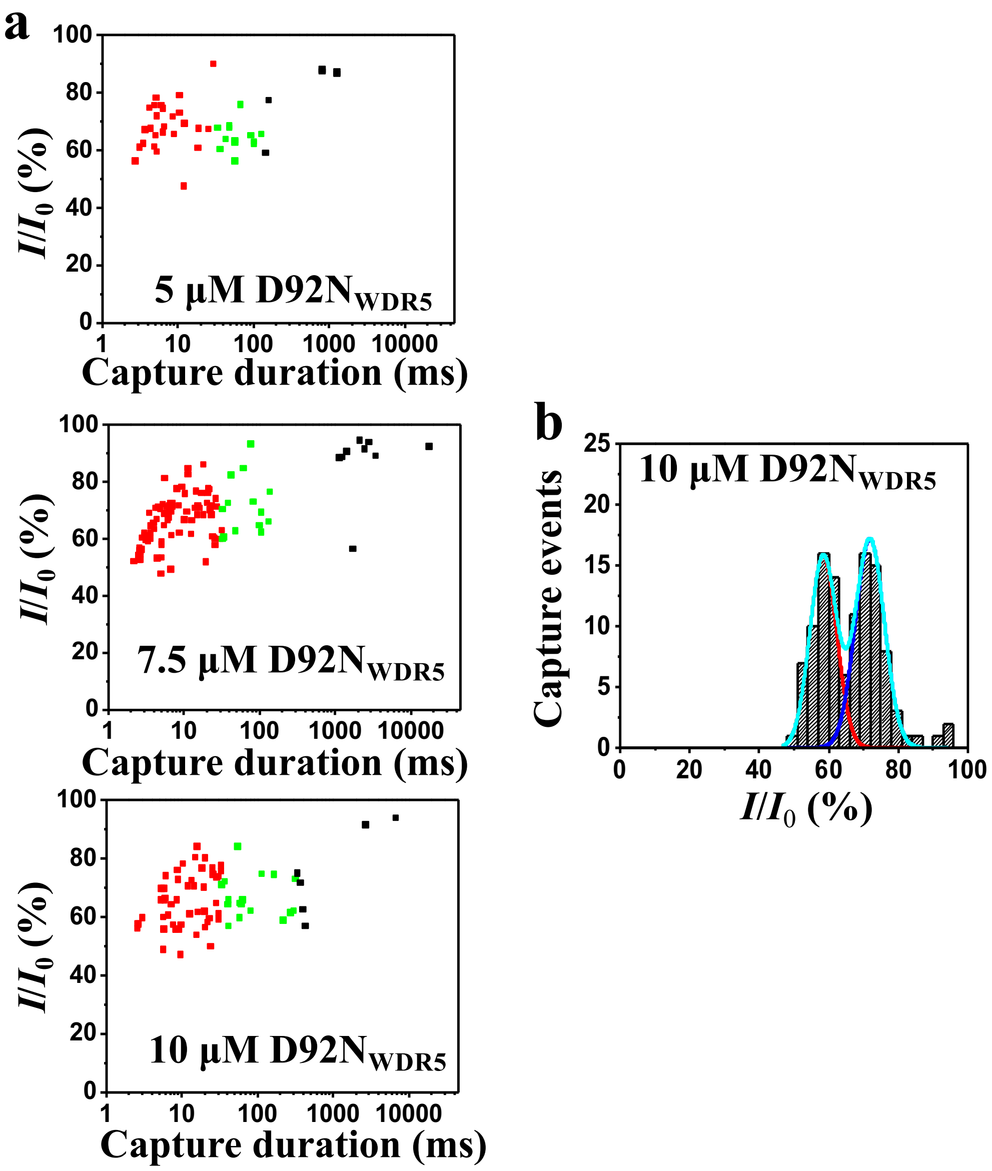


**Supplementary Figure 25. Scatter plots of normalized current blockades versus D92N$_{WDR5}$ capture durations.** Here, $I_0$ and $I$ denote the single-channel current of the D92N$_{WDR5}$-released substate of MLL4$_{Win}$tFhuA and the amplitude of D92N$_{WDR5}$-produced current blockades, respectively. **(a)** Semilogarithmic scatter plot of $I/I_0$ as a function of capture duration of MLL4$_{Win}$tFhuA in the presence of 2 μM D92N$_{WDR5}$, 4 μM D92N$_{WDR5}$ and 10 μM D92N$_{WDR5}$, respectively. The short-, medium-, and long-lived events are represented in red, green, and black, respectively. **(b)** Event histograms of the D92N$_{WDR5}$-capture current blockades ($I/I_0$). The cumulative fit of each histogram is represented by a cyan curve. The red and blue curves indicate fit components of a smaller and a larger blockade, respectively. For 10 μM D92N$_{WDR5}$, these current blockades (mean ± s.e.m.) were 59 ± 0.6 s and 73 ± 0.9 s, respectively (number of events: $N = 112$). The applied transmembrane potential was −20 mV. For the analysis of binding events, single-channel electrical traces were low-pass filtered using an 8-pole Bessel filter at a frequency of 1 kHz. Data was extracted from 20 minute-long single-channel electrical recordings. Source data are provided as a Source Data file.

**Supplementary Table 1. Alignment of the amino acid sequence of the 14-residue $MLL4_{Win}$ ligand.** The sequence of $MLL4_{Win}$ includes a highly conserved 6-residue Win motif peptide (marked in red), along with 4 flanking residues on each side (e.g., $P_{-7}$ through $P_{-4}$ and $P_3$ through $P_6$). The minimal 6-residue Win motif peptide of $MLL4_{Win}$ has to partition into the WDR5 cavity, forming the highly specific $MLL4_{Win}$–WDR5 interactions.[1, 8]

| Peptide | $P_{-7}$ | $P_{-6}$ | $P_{-5}$ | $P_{-4}$ | $P_{-3}$ | $P_{-2}$ | $P_{-1}$ | $P_0$ | $P_1$ | $P_2$ | $P_3$ | $P_4$ | $P_5$ | $P_6$ |
|---|---|---|---|---|---|---|---|---|---|---|---|---|---|---|
| $MLL4_{Win}$ ($MLL4^{2504\text{-}2517}$) | L | N | P | H | G | A | A | R | A | E | V | Y | L | R |

**Supplementary Table 2. List of hydrogen bonds at the MLL4$_{Win}$–WDR5 interface.** For listing these hydrogen bonds, we used co-crystallization data reported previously by Dharmarajan and co-workers.[1] We employed a cut-off distance of 3.2 Å. The first and second residue in each hydrogen bond belong to MLL4$_{Win}$ and WDR5, respectively. Only a sequence of 12 residues of MLL4$_{Win}$ was able to model these interactions. bb and sc denote the backbone and side chain of the residue, respectively.

| Peptide | Hydrogen Bonds | Interaction Site | Distance (Å) |
|---|---|---|---|
| **MLL4$_{Win}$** | A2510 – D107 | bb-sc | 3.0 |
| **LNPHGAARAEVY** | R2511 – S91 | bb-sc, sc-bb | 3.0, 2.8 |
| | R2511 – C261 | sc-bb | 2.9 |
| | R2511 – F133 | sc-bb | 3.0 |

**Supplementary Table 3**. **WDR5-released durations at lower [WDR5] values.** $\tau_{on}$ is the WDR5-released duration for all four experiments at listed [WDR5] values.

| **n** | **[WDR5] (µM)** | $\boldsymbol{\tau_{on}}$ **(s)** |
|---|---|---|
| 4 | 0.5 | 8.46 ± 1.30 |
| 4 | 0.8 | 4.86 ± 0.56 |

Values in the table are mean ± s.d. *n* is number of independently reconstituted nanopores. The other experimental conditions were the same as those stated in Methods.

**Supplementary Table 4. Values of the WDR5-released durations ($\tau_{on\text{-}i}$) and WDR5-captured durations ($\tau_{off\text{-}i}$) for three binding events and for all [WDR5] values.** Subscript "i" is 1, 2, and 3 for short-, medium-, and long-lived WDR5 capture events, respectively. $\tau_{on}$ are mean values of the single-exponential distributions of WDR5-released duration histograms. $\tau_{on\text{-}1} = \tau_{on}/P_1$, where $P_1$ is the event probability of the short-lived events for each experiment. $\tau_{on\text{-}2}$ and $\tau_{on\text{-}3}$ were calculated using the same equation and respective probabilities. The mean values of those probabilities are listed in Supplementary Table 5. $\tau_{off}$ values are the mean values of the three-exponential distributions of WDR5-captured duration histograms. All histogram fittings were conducted using a semilogarithmic representation. The maximum likelihood method[5, 9] and logarithm likelihood ratio (LLR)[10-12] tests were used for all fittings to determine the best multi-exponential distribution model (Methods).

| n | [WDR5] (µM) | $\tau_{on}$ (s) | $\tau_{on\text{-}1}$ (s) | $\tau_{on\text{-}2}$ (s) | $\tau_{on\text{-}3}$ (s) | $\tau_{off\text{-}1}$ (s) | $\tau_{off\text{-}2}$ (s) | $\tau_{off\text{-}3}$ (s) |
|---|---|---|---|---|---|---|---|---|
| 4 | 1 | 4.2 ± 0.3 | 7.3 ± 0.6 | 15 ± 1 | 28 ± 2 | 0.012 ± 0.002 | 0.12 ± 0.05 | 1.4 ± 0.4 |
| 4 | 2 | 2.0 ± 0.4 | 3.6 ± 0.7 | 7.1 ± 1.5 | 14 ± 3 | 0.010 ± 0.002 | 0.06 ± 0.02 | 1.4 ± 0.5 |
| 3 | 4 | 1.3 ± 0.5 | 2.2 ± 0.8 | 4.3 ± 1.5 | 8.4 ± 2.9 | 0.011 ± 0.002 | 0.12 ± 0.03 | 2.0 ± 0.5 |
| 3 | 6 | 0.70 ± 0.02 | 1.2 ± 0.1 | 2.4 ± 0.1 | 4.7 ± 0.2 | 0.011 ± 0.003 | 0.11 ± 0.07 | 2.1 ± 0.9 |
| 3 | 8 | 0.55 ± 0.10 | 0.96 ± 0.17 | 1.9 ± 0.3 | 3.7 ± 0.7 | 0.013 ± 0.004 | 0.07 ± 0.04 | 1.1 ± 0.2 |
| 3 | 10 | 0.40 ± 0.08 | 0.82 ± 0.49 | 1.4 ± 0.3 | 2.7 ± 0.5 | 0.011 ± 0.003 | 0.09 ± 0.06 | 1.4 ± 0.8 |

Values were provided as mean ± s.d. *n* is the number of independently reconstituted nanopores. The other experimental conditions were the same as those stated in Methods. Source data are provided as a Source Data file.

**Supplementary Table 5. The probability distribution of the three binding events noted with WDR5-MLL4$_{Win}$ interactions.** These events were differentiated by the WDR5-captured duration. Individual experimental values were derived using event-list histograms in ClampFit (Axon) and fittings of event histograms in a semi-logarithmic representation. The maximum likelihood method[5, 9] and logarithm likelihood ratio (LLR)[10-12] tests were used for all fittings to determine the best multi-exponential distribution model (Methods).

| ***n*** | **[WDR5] (µM)** | $P_1$ | $P_2$ | $P_3$ |
|---|---|---|---|---|
| 4 | 1 | 0.56 ± 0.11 | 0.29 ± 0.12 | 0.15 ± 0.09 |
| 4 | 2 | 0.59 ± 0.05 | 0.30 ± 0.09 | 0.12 ± 0.04 |
| 3 | 4 | 0.58 ± 0.04 | 0.27 ± 0.04 | 0.16 ± 0.06 |
| 3 | 6 | 0.58 ± 0.09 | 0.28 ± 0.07 | 0.15 ± 0.02 |
| 3 | 8 | 0.57 ± 0.04 | 0.31 ± 0.03 | 0.18 ± 0.07 |
| 3 | 10 | 0.56 ± 0.06 | 0.29 ± 0.07 | 0.15 ± 0.06 |

Values were provided as mean ± s.d. *n* is the number of independently reconstituted nanopores. $P_1$, $P_2$, and $P_3$ are the probabilities of the short-, medium-, and long-lived events, respectively. The other experimental conditions were the same as those stated in Methods. Source data are provided as a Source Data file.

**Supplementary Table 6. Values of the mean normalized amplitude of WDR5-produced current blockades, $I/I_0$, for three binding events and at various [WDR5] values.** Here, $I_0$ and $I$ denote the single-channel current of the WDR5-released substate of $MLL4_{Win}$tFhuA and the amplitude of WDR5-produced current blockades, respectively. The $I/I_0$ ratio was then converted into percentage. Event histograms of normalized current amplitudes are shown in Fig. 2g**.**

| **n** | **[WDR5] (μM)** | **Probability of smaller $I/I_0$** | **Smaller $I/I_0$ (%)** | **Probability of larger $I/I_0$** | **Larger $I/I_0$ (%)** |
|---|---|---|---|---|---|
| 4 | 2 | 0.56 ± 0.04 | 61 ± 3 | 0.44 ± 0.04 | 72 ± 2 |
| 3 | 4 | 0.53 ± 0.02 | 60 ± 1 | 0.47 ± 0.02 | 69 ± 2 |
| 3 | 10 | 0.54 ± 0.03 | 59 ± 3 | 0.46 ± 0.02 | 71 ± 3 |

Values were provided as mean ± s.d. *n* is the number of independently reconstituted nanopores. The other experimental conditions were the same as those stated in Methods. Source data are provided as a Source Data file.

**Supplementary Table 7. The association and dissociation rate constants for three binding events and for all WDR5 concentrations.** The association rate constants of the short-lived events, $k_{on-1}$, were determined using the equation: $k_{on-1} = 1/(\tau_{on-1}[WDR5])$. The association rate constants of the medium-lived events, $k_{on-2}$, and long-lived events, $k_{on-3}$, were determined in the same way. $\tau_{on-i}$ values are provided in Supplementary Table 4. The dissociation rate constants of the short-lived events were determined using the equation: $k_{off-1} = 1/\tau_{off-1}$. We used the same approach for determining the dissociation rate constants of the medium-lived events, $k_{off-2}$, and long-lived events, $k_{off-3}$. $\tau_{off-i}$ values are listed in Supplementary Table 4.

| *n* | [WDR5] (μM) | $k_{on-1} \times 10^{-5}$ ($M^{-1}s^{-1}$) | $k_{on-2} \times 10^{-4}$ ($M^{-1}s^{-1}$) | $k_{on-3} \times 10^{-4}$ ($M^{-1}s^{-1}$) | $k_{off-1}$ ($s^{-1}$) | $k_{off-2}$ ($s^{-1}$) | $k_{off-3}$ ($s^{-1}$) |
|---|---|---|---|---|---|---|---|
| 4 | 1 | 1.4 ± 0.1 | 6.9 ± 0.5 | 3.5 ± 0.3 | 85 ± 11 | 8.2 ± 1.5 | 0.73 ± 0.21 |
| 4 | 2 | 1.4 ± 0.3 | 7.3 ± 1.5 | 3.8 ± 0.8 | 93 ± 12 | 8.9 ± 3.5 | 0.80 ± 0.37 |
| 3 | 4 | 1.2 ± 0.4 | 6.2 ± 1.9 | 3.2 ± 1.0 | 88 ± 12 | 9.3 ± 4.2 | 0.51 ± 0.11 |
| 3 | 6 | 1.4 ± 0.5 | 6.9 ± 0.3 | 3.5 ± 0.1 | 84 ± 23 | 11 ± 4 | 0.57 ± 0.31 |
| 3 | 8 | 1.3 ± 0.3 | 6.8 ± 1.3 | 3.5 ± 0.7 | 82 ± 21 | 9.0 ± 1.1 | 0.89 ± 0.21 |
| 3 | 10 | 1.5 ± 0.2 | 7.4 ± 1.3 | 3.8 ± 0.1 | 90 ± 18 | 11 ± 6 | 0.92 ± 0.51 |

Values were provided as mean ± s.d. *n* is number of independently reconstituted nanopores. The other experimental conditions were the same as those stated in Methods. Source data are provided as a Source Data file.

**Supplementary Table 8. The association and dissociation rate constants of the three binding events noted with WDR5.** $k_{on}$ values are the slopes of the linear line fits in Fig. 2h. $k_{off}$ values are the axis intercepts of the horizontal line fits in Fig. 2i.

| $k_{on-1} \times 10^{-5}$ ($M^{-1}s^{-1}$) | $k_{on-2} \times 10^{-4}$ ($M^{-1}s^{-1}$) | $k_{on-3} \times 10^{-4}$ ($M^{-1}s^{-1}$) | $k_{off-1}$ ($s^{-1}$) | $k_{off-2}$ ($s^{-1}$) | $k_{off-3}$ ($s^{-1}$) |
|---|---|---|---|---|---|
| 1.4 ± 0.1 | 6.9 ± 1.8 | 3.6 ± 1.0 | 86 ± 2 | 9.2 ± 0.5 | 0.78 ± 0.06 |

Values were provided as mean ± s.e.m. Details about *n*, the number of independently reconstituted nanopores at each [WDR5] value, are provided in Supplementary Table 7. The other experimental conditions were the same as those stated in Methods.

**Supplementary Table 9. The equilibrium dissociation constants of the three binding events of $MLL4_{Win}$ –WDR5 interactions.** $K_D$ values were determined using the equation: $K_D = k_{off}/k_{on}$. $K_{D-1}$, $K_{D-2}$, and $K_{D-3}$ are the equilibrium dissociation constants for the short-, medium-, and long-lived WDR5-captured events, respectively. The respective $k_{on}$ and $k_{off}$ values can be found in Table 7.

| ***n*** | ***K*$_{D-1}$ (µM)** | ***K*$_{D-2}$ (µM)** | ***K*$_{D-3}$ (µM)** |
|---|---|---|---|
| 20 | 631 ± 49 | 138 ± 18 | 20 ± 4 |

Values were provided as mean ± s.d. *n* is number of independently reconstituted nanopores. The other experimental conditions were the same as those stated in Methods. Source data are provided as a Source Data file.

**Supplementary Table 10. Quantitative comparisons of kinetic and equilibrium constants between single-molecule electrical recordings and bulk-phase BLI measurements.** All nanopore data were extracted from Supplementary Tables 8-9. BLI data were derived from fittings of sensorgrams (Methods).

| **Method** | **Event type** | **$k_{on}$ ($M^{-1}s^{-1}$)** | **$k_{off}$ ($s^{-1}$)** | **$K_D$ (M)** |
|---|---|---|---|---|
| Nanopore | 1 | $(1.4 \pm 0.1) \times 10^5$ | $86 \pm 2$ | $(631 \pm 49) \times 10^{-6}$ |
| | 2 | $(6.9 \pm 1.8) \times 10^4$ | $9.2 \pm 0.5$ | $(138 \pm 18) \times 10^{-6}$ |
| | 3 | $(3.6 \pm 1.0) \times 10^4$ | $0.78 \pm 0.06$ | $(20 \pm 4) \times 10^{-6}$ |
| BLI | | $(2.6 \pm 0.1) \times 10^4$ | $(1.2 \pm 0.1) \times 10^{-2}$ | $(0.46 \pm 0.04) \times 10^{-6}$ |

BLI values were provided mean ± s.d. using three independent experiments. The other experimental conditions were the same as those stated in Methods. Source data are provided as a Source Data file.

**Supplementary Table 11. Values of the $D92N_{WDR5}$-released durations ($\tau_{on}$) and $D92N_{WDR5}$-captured durations ($\tau_{off}$) for three binding events and for all [$D92N_{WDR5}$] values.** Subscript "i" is 1, 2, and 3 for short-, medium-, and long-lived $D92N_{WDR5}$ capture events, respectively. $\tau_{on}$ are mean values of the single-exponential distributions of $D92N_{WDR5}$-released duration histograms. $\tau_{on-1} = \tau_{on} / P_1$, where $P_1$ is the event probability of the short-lived events for each experiment. $\tau_{on-2}$ and $\tau_{on-3}$ were calculated using the same equation and respective probabilities. The mean values of those probabilities are listed in Supplementary Table 12. $\tau_{off}$ values are the mean values of the three-exponential distributions of $D92N_{WDR5}$-captured duration histograms. All histogram fittings were conducted using a semilogarithmic representation. The maximum likelihood method[5, 9] and logarithm likelihood ratio (LLR)[10-12] tests were used for all fittings to determine the best multi-exponential distribution model (Methods).

| ***n*** | **[$D92N_{WDR5}$] (μM)** | **$\tau_{on}$ (s)** | **$\tau_{on-1}$ (s)** | **$\tau_{on-2}$ (s)** | **$\tau_{on-3}$ (s)** | **$\tau_{off-1}$ (s)** | **$\tau_{off-2}$ (s)** | **$\tau_{off-3}$ (s)** |
|---|---|---|---|---|---|---|---|---|
| 3 | 5 | 8.6 ± 3.5 | 15 ± 6 | 28 ± 11 | 86 ± 35 | 0.010 ± 0.004 | 0.15 ± 0.04 | 1.1 ± 0.4 |
| 3 | 7.5 | 4.7 ± 0.9 | 7.9 ± 1.7 | 15 ± 3 | 47 ± 10 | 0.010 ± 0.001 | 0.12 ± 0.07 | 1.9 ± 0.9 |
| 3 | 10 | 2.6 ± 0.8 | 4.4 ± 1.4 | 8.5 ± 2.5 | 26 ± 8 | 0.010 ± 0.006 | 0.21 ± 0.09 | 3.0 ± 0.9 |

Values were provided as mean ± s.d. *n* is the number of independently reconstituted nanopores. The other experimental conditions were the same as those stated in Methods. Source data are provided as a Source Data file.

**Supplementary Table 12. The probability distribution of the three binding events noted with MLL4$_{Win}$-D92N$_{WDR5}$ interactions.** These events were differentiated by the D92N$_{WDR5}$-captured duration. Individual experimental values were derived using event-list histograms in ClampFit (Axon) and fittings of event histograms in a semi-logarithmic representation. The maximum likelihood method[5, 9] and logarithm likelihood ratio (LLR)[10-12] tests were used for all fittings to determine the best multi-exponential distribution model (Methods).

| ***n*** | **[D92N$_{WDR5}$] (μM)** | ***P*$_1$** | ***P*$_2$** | ***P*$_3$** |
|---|---|---|---|---|
| 3 | 5 | 0.52 ± 0.15 | 0.37 ± 0.06 | 0.12 ± 0.06 |
| 3 | 7.5 | 0.61 ± 0.06 | 0.27 ± 0.03 | 0.11 ± 0.04 |
| 3 | 10 | 0.52 ± 0.05 | 0.30 ± 0.09 | 0.17 ± 0.09 |

Values were provided as mean ± s.d. *n* is the number of independently reconstituted nanopores. $P_1$, $P_2$, and $P_3$ are the event probabilities of the short-, medium-, and long-lived events, respectively. The other experimental conditions were the same as those stated in Methods. Source data are provided as a Source Data file.

**Supplementary Table 13. The association and dissociation rate constants for three binding events and for all $D92N_{WDR5}$ concentrations.** The association rate constants of the short-lived events, $k_{on-1}$, were determined using the equation: $k_{on-1} = 1/(\tau_{on-1}[D92N_{WDR5}])$. The association rate constants of the medium-lived events, $k_{on-2}$, and long-lived events, $k_{on-3}$, were determined in the same way. $\tau_{on-i}$ values are provided in Supplementary Table 11. The dissociation rate constants of the short-lived events were determined using the equation: $k_{off-1} = 1/\tau_{off-1}$. We used the same approach for determining the dissociation rate constants of the medium-lived events, $k_{off-2}$, and long-lived events, $k_{off-3.}$ $\tau_{on-i}$ values are given in Supplementary Table 11.

| n | $[D92N_{WDR5}]$ (µM) | $k_{on-1} \times 10^{-4}$ $(M^{-1}s^{-1})$ | $k_{on-2} \times 10^{-3}$ $(M^{-1}s^{-1})$ | $k_{on-3} \times 10^{-3}$ $(M^{-1}s^{-1})$ | $k_{off-1}$ $(s^{-1})$ | $k_{off-2}$ $(s^{-1})$ | $k_{off-3}$ $(s^{-1})$ |
|---|---|---|---|---|---|---|---|
| 3 | 5 | 1.6 ± 0.8 | 9.8 ± 4.7 | 3.2 ± 1.5 | 93 ± 27 | 6.8 ± 1.9 | 0.73 ± 0.11 |
| 3 | 7.5 | 1.7 ± 0.3 | 9.1 ± 1.8 | 3.0 ± 0.6 | 86 ± 18 | 6.2 ± 0.9 | 0.61 ± 0.30 |
| 3 | 10 | 2.0 ± 0.4 | 11 ± 2 | 3.4 ± 0.6 | 85 ± 26 | 6.8 ± 3.2 | 0.64 ± 0.27 |

Values were provided as mean ± s.d. *n* is the number of independently reconstituted nanopores. The other experimental conditions were the same as those stated in Methods. Source data are provided as a Source Data file.

**Supplementary Table 14. The association and dissociation rate constants for the three binding events of MLL4$_{Win}$-D92N$_{WDR5}$ interactions.** $k_{on}$ values were derived as slopes of the linear fits in Supplementary Fig. 14a. $k_{off}$ values are the axis intercepts of the horizontal line fits in Supplementary Fig. 14b.

| $k_{on-1} \times 10^{-4}$ ($M^{-1}s^{-1}$) | $k_{on-2} \times 10^{-3}$ ($M^{-1}s^{-1}$) | $k_{on-3} \times 10^{-3}$ ($M^{-1}s^{-1}$) | $k_{off-1}$ ($s^{-1}$) | $k_{off-2}$ ($s^{-1}$) | $k_{off-3}$ ($s^{-1}$) |
|---|---|---|---|---|---|
| 1.8 ± 0.1 | 9.6 ± 0.7 | 3.1 ± 0.2 | 87 ± 2 | 6.6 ± 0.5 | 0.65 ± 0.13 |

Values were provided as mean ± s.e.m. Details about the number, *n*, of independently reconstituted nanopores at each D92N$_{WDR5}$ concentration are provided in Supplementary Table 13. The other experimental conditions were the same as those stated in Methods. Source data are provided as a Source Data file.

**Supplementary Table 15**. **The equilibrium dissociation constants for the three binding events of $MLL4_{Win}$–$D92N_{WDR5}$ interactions.** $K_D$ values were determined using the equation: $K_D = k_{off}/k_{on}$. $K_{D-1}$, $K_{D-2}$, and $K_{D-3}$ are the equilibrium dissociation constants for the short-, medium-, and long-lived $D92N_{WDR5}$-captured events, respectively. The $k_{on}$ and $k_{off}$ values can be found in Supplementary Table 13.

| n | $K_{D-1}$ (mM) | $K_{d-2}$ (µM) | $K_{d-3}$ (µM) |
|---|---|---|---|
| 9 | 5.4 ± 1.3 | 721 ± 18 | 218 ± 22 |

Values were provided as mean ± s.d. *n* is the number of independently reconstituted nanopores. The other experimental conditions were the same as those stated in Methods. Source data are provided as a Source Data file.

**Supplementary Table 16. Comparisons of rate constants, equilibrium dissociation constants, and Gibbs free energy values between WDR5 and D92N$_{WDR5}$.** The $k_{on}$ and $k_{off}$ values are extracted from Supplementary Table 8 and Table 14. $K_D$ values can be found in Supplementary Table 9 and Table 15. Gibbs free energy, $\Delta G$, was calculated using the equation $\Delta G = RT\ln K_D$. Here, R and $T$ indicate the general gas constant and the absolute room temperature, respectively. $k_B$ is the Boltzmann constant.

| | Event type | WDR5 | D92N$_{WDR5}$ |
|---|---|---|---|
| **$k_{on}$ ($M^{-1}s^{-1}$)** | 1 | $(1.4 \pm 0.1) \times 10^5$ | $(1.8 \pm 0.1) \times 10^4$ |
| | 2 | $(6.9 \pm 1.8) \times 10^4$ | $(9.6 \pm 0.7) \times 10^3$ |
| | 3 | $(3.6 \pm 1.0) \times 10^4$ | $(3.1 \pm 0.2) \times 10^3$ |
| **$k_{off}$ ($s^{-1}$)** | 1 | 86 ± 2 | 87 ± 2 |
| | 2 | 9.2 ± 0.5 | 6.6 ± 0.5 |
| | 3 | 0.78 ± 0.06 | 0.65 ± 0.13 |
| **$K_D$ (M)** | 1 | $(631 \pm 49) \times 10^{-6}$ | $(5.4 \pm 1.3) \times 10^{-3}$ |
| | 2 | $(138 \pm 18) \times 10^{-6}$ | $(721 \pm 18) \times 10^{-6}$ |
| | 3 | $(20 \pm 4) \times 10^{-6}$ | $(218 \pm 22) \times 10^{-6}$ |
| **$\Delta G$ (kcal/mol)** | 1 | −4.3 ± 0.05 | −3.1 ± 0.1 |
| | 2 | −5.2 ± 0.07 | −4.2 ± 0.1 |
| | 3 | −6.3 ± 0.11 | −4.9 ± 0.1 |
| **$\Delta G$ ($k_BT$)** | 1 | −7.4 ± 0.1 | −5.3 ± 0.2 |
| | 2 | −8.8 ± 0.1 | −7.2 ± 0.1 |
| | 3 | −11 ± 1 | −8.4 ± 0.1 |

Values were provided as mean ± s.d. The other experimental conditions were the same as those stated in Methods. Source data are provided as a Source Data file.

**Supplementary Table 17. Change in Gibbs free energy for WDR5 and D92N$_{WDR5}$.** $\Delta\Delta G$ was calculated using the equation $\Delta\Delta G = \Delta G$ (WDR5) – $\Delta G$ (D92N$_{WDR5}$). $\Delta G$ (WDR5) and $\Delta G$ (D92N$_{WDR5}$) can be found in Supplementary Table 16.

| Event type | $\Delta\Delta G^o$ (kcal/mol) | $\Delta\Delta G^o$ ($k_BT$) |
|---|---|---|
| 1 | −1.2 | −2.1 |
| 2 | −1.0 | −1.6 |
| 3 | −1.4 | −2.4 |

**Supplementary Table 18. Transition rate constants obtained for the interconversion-dependent kinetic model.** Raw single-channel event data from 10 µM WDR5 and 10 µM $D92N_{WDR5}$ were analyzed using MATLAB (MathWorks, Natick, MA). Values were provided as mean ± s.d. ($n$ = 3), where $n$ is the number of independently reconstituted nanopores. The association rate constants $k_{on\text{-}1}$, $k_{on\text{-}2}$ and $k_{on\text{-}3}$, obtained by this model were the same as those determined by the interconversion-independent kinetic model. The interconversion-independent and interconversion-dependent kinetic models are illustrated in Supplementary Fig. 15 and Fig. 16, respectively.

| Protein | $k_{off-1}$ ($s^{-1}$) | $k_{12}$ ($s^{-1}$) | $k_{13}$ ($s^{-1}$) | $k_{21}$ ($s^{-1}$) | $k_{off-2}$ ($s^{-1}$) | $k_{23}$ ($s^{-1}$) | $k_{31}$ ($s^{-1}$) | $k_{32}$ ($s^{-1}$) | $k_{off-3}$ ($s^{-1}$) |
|---|---|---|---|---|---|---|---|---|---|
| WDR5 | 73 ± 4 | 0.10 ± 0.01 | 0.27 ± 0.30 | 2.7 ± 1.7 | 5.3 ± 2.6 | 0.05 ± 0.05 | 0.09 ± 0.03 | 0.13 ± 0.17 | 0.36 ± 0.22 |
| $D92N_{WDR5}$ | 98 ± 9 | 5.1 ± 3.1 | 5.1 ± 3.1 | 2.4 ± 1.6 | 3.9 ± 2.2 | 0.80 ± 0.24 | 0.17 ± 0.03 | 0.21 ± 0.06 | 0.16 ± 0.03 |

**Supplementary Table 19. Event probabilities determined by the interconversion-dependent kinetic model.** This table shows event probabilities for the three distinct events. Raw single-channel event data from 10 µM WDR5 and 10 µM D92N$_{WDR5}$ were analyzed using MATLAB. Values were provided as mean ± s.d. ($n$ = 3), where $n$ is the number of independently reconstituted nanopores. $P_1$, $P_2$, and $P_3$ are the event probabilities of the short-, medium-, and long-lived capture events, respectively. The interconversion-independent and interconversion-dependent kinetic models are illustrated in Supplementary Fig. 15 and Fig. 16, respectively. Source data are provided as a Source Data file.

| **Protein** | $P_1$ | $P_2$ | $P_3$ |
|---|---|---|---|
| WDR5 | 0.55 ± 0.03 | 0.29 ± 0.03 | 0.17 ± 0.03 |
| D92N$_{WDR5}$ | 0.52 ± 0.06 | 0.41 ± 0.05 | 0.07 ± 0.05 |

**Supplementary Table 20. Values of the D172A$_{WDR5}$-released durations ($\tau_{on}$) and D172A$_{WDR5}$-captured durations ($\tau_{off}$) for three binding events and for all [D172A$_{WDR5}$] values.** Subscript "i" is 1, 2, and 3 for short-, medium-, and long-lived D172A$_{WDR5}$ capture events, respectively. $\tau_{on}$ are mean values of the single-exponential distributions of D172A$_{WDR5}$-released duration histograms. $\tau_{on-1} = \tau_{on} / P_1$, where $P_1$ is the event probability of the short-lived events for each experiment. $\tau_{on-2}$ and $\tau_{on-3}$ were calculated using the same equation and respective probabilities. The mean values of those probabilities are listed in Supplementary Table 21. $\tau_{off}$ values are the mean values of the three-exponential distributions of D172A$_{WDR5}$-captured duration histograms. All histogram fittings were conducted using a semilogarithmic representation. The maximum likelihood method[5, 9] and logarithm likelihood ratio (LLR)[10-12] tests were used for all fittings to determine the best multi-exponential distribution model (Methods).

| *n* | [D172A$_{WDR5}$] (µM) | $\tau_{on}$ (s) | $\tau_{on-1}$ (s) | $\tau_{on-2}$ (s) | $\tau_{on-3}$ (s) | $\tau_{off-1}$ (s) | $\tau_{off-2}$ (s) | $\tau_{off-3}$ (s) |
|---|---|---|---|---|---|---|---|---|
| 3 | 2 | 2.2 ± 0.3 | 4.0 ± 0.7 | 7.2 ± 1.2 | 18 ± 3 | 0.010 ± 0.002 | 0.14 ± 0.03 | 1.7 ± 0.4 |
| 3 | 4 | 1.2 ± 0.1 | 2.3 ± 0.2 | 4.1 ± 0.5 | 13 ± 4 | 0.011 ± 0.003 | 0.10 ± 0.01 | 1.6 ± 0.5 |
| 3 | 8 | 0.46 ± 0.1 | 0.82 ± 0.2 | 1.5 ± 0.3 | 4.2 ± 1.3 | 0.011 ± 0.002 | 0.13 ± 0.03 | 1.2 ± 0.1 |

Values were provided as mean ± s.d. *n* is the number of independently reconstituted nanopores. The other experimental conditions were the same as those stated in Methods. Source data are provided as a Source Data file.

**Supplementary Table 21. The probability distribution of the three different events noted with D172A$_{WDR5}$-MLL4$_{Win}$ interactions.** These events were differentiated by the D172A$_{WDR5}$-captured duration. Individual experimental values were derived using event-list histograms in ClampFit (Axon) and fittings of event histograms in a semi-logarithmic representation. The maximum likelihood method[5, 9] and logarithm likelihood ratio (LLR)[10-12] tests were used for all fittings to determine the best multi-exponential distribution model (Methods).

| ***n*** | **[D172A$_{WDR5}$] (µM)** | $P_1$ | $P_2$ | $P_3$ |
|---|---|---|---|---|
| 3 | 2 | 0.57 ± 0.05 | 0.31 ± 0.03 | 0.12 ± 0.03 |
| 3 | 4 | 0.54 ± 0.04 | 0.32 ± 0.05 | 0.11 ± 0.02 |
| 3 | 8 | 0.56 ± 0.02 | 0.32 ± 0.06 | 0.12 ± 0.04 |

Values were provided as mean ± s.d. *n* is the number of independently reconstituted nanopores. $P_1$, $P_2$, and $P_3$ are the event probabilities of the short-, medium-, and long-lived events, respectively. The other experimental conditions were the same as those stated in Methods. Source data are provided as a Source Data file.

**Supplementary Table 22. The values of the mean normalized amplitude of $D172A_{WDR5}$-produced current blockades, $I/I_0$, and at various [$D172A_{WDR5}$] values.** Here, $I_0$ and $I$ denote the single-channel current of the $D172A_{WDR5}$-released substate of $MLL4_{Win}$tFhuA and the amplitude of $D172A_{WDR5}$-produced current blockades, respectively. The $I/I_0$ ratio was then converted into percentage.

| n | [$D172A_{WDR5}$] (μM) | Probability of smaller $I/I_0$ | Smaller $I/I_0$ (%) | Probability of larger $I/I_0$ | Larger $I/I_0$ (%) |
|---|---|---|---|---|---|
| 4 | 2 | 0.53 ± 0.03 | 61 ± 3 | 0.47 ± 0.03 | 71 ± 3 |
| 3 | 4 | 0.55 ± 0.04 | 61 ± 2 | 0.45 ± 0.04 | 73 ± 2 |
| 3 | 8 | 0.54 ± 0.03 | 60 ± 2 | 0.46 ± 0.02 | 73 ± 3 |

Values were provided as mean ± s.d. *n* was the number of independently reconstituted nanopores. The other experimental conditions were the same as those stated in Methods. Source data are provided as a Source Data file.

**Supplementary Table 23. The association and dissociation rate constants for three binding events and for all D172A$_{WDR5}$ concentrations.** The association rate constants of the short-lived events, $k_{on-1}$, were determined using the equation: $k_{on-1} = 1/(\tau_{on-1}[D172A_{WDR5}])$. The association rate constants of the medium-lived events, $k_{on-2}$, and long-lived events, $k_{on-3}$, were determined in the same way. $\tau_{on-i}$ values are provided in Supplementary Table 20. The dissociation rate constants of the short-lived events were determined using the equation: $k_{off-1} = 1/\tau_{off-1}$. We used the same approach for determining the dissociation rate constants of the medium-lived events, $k_{off-2}$, and long-lived events, $k_{off-3}$. $\tau_{off-i}$ values are listed in Supplementary Table 20.

| ***n*** | **[D172A$_{WDR5}$] (μM)** | **$k_{on-1}$× $10^{-5}$ ($M^{-1}s^{-1}$)** | **$k_{on-2}$× $10^{-4}$ ($M^{-1}s^{-1}$)** | **$k_{on-3}$× $10^{-4}$ ($M^{-1}s^{-1}$)** | **$k_{off-1}$ ($s^{-1}$)** | **$k_{off-2}$ ($s^{-1}$)** | **$k_{off-3}$ ($s^{-1}$)** |
|---|---|---|---|---|---|---|---|
| 3 | 2 | 1.3 ± 0.2 | 7.1 ± 1.1 | 2.8 ± 0.3 | 94 ± 16 | 7.4 ± 1.9 | 0.58 ± 0.12 |
| 3 | 4 | 1.1 ± 0.1 | 6.2 ± 0.7 | 3.5 ± 0.6 | 100 ± 29 | 9.9 ± 2.2 | 0.66 ± 0.22 |
| 3 | 8 | 1.6 ± 0.3 | 6.9 ± 1.9 | 3.2 ± 1.1 | 87 ± 24 | 8.2 ± 1.9 | 0.93 ± 0.20 |

Values were provided as mean ± s.d. *n* is number of independently reconstituted nanopores. The other experimental conditions were the same as those stated in Methods. Source data are provided as a Source Data file.

**Supplementary Table 24. The average association and dissociation rate constants for the three unique events noted with D172A$_{WDR5}$.** $k_{on}$ values is the slope of the linear line fits in Supplementary Fig. 19c. $k_{off}$ values are the axis intercepts of the horizontal line fits in Supplementary Fig. 19d.

| $k_{on-1} \times 10^{-5}$ ($M^{-1}s^{-1}$) | $k_{on-2} \times 10^{-4}$ ($M^{-1}s^{-1}$) | $k_{on-3} \times 10^{-4}$ ($M^{-1}s^{-1}$) | $k_{off-1}$ ($s^{-1}$) | $k_{off-2}$ ($s^{-1}$) | $k_{off-3}$ ($s^{-1}$) |
|---|---|---|---|---|---|
| 1.3 ± 0.03 | 6.7 ± 0.9 | 3.0 ± 1.6 | 91 ± 6 | 9.3 ± 0.6 | 0.79 ± 0.09 |

Values were provided as mean ± s.e.m. Details about the number, *n*, of independently reconstituted nanopores at each [D172A$_{WDR5}$] value are provided in Supplementary Table 23. The other experimental conditions were the same as those stated in Methods.

**Supplementary Table 25**. **The equilibrium dissociation constants for the three binding events of $MLL4_{Win}$–$D172A_{WDR5}$ interactions.** $K_D$ values were determined using the equation: $K_D = k_{off}/k_{on}$. $K_{D-1}$, $K_{D-2}$, and $K_{D-3}$ are the equilibrium dissociation constants for the short-, medium-, and long-lived $D172A_{WDR5}$-captured events, respectively. The respective $k_{on}$ and $k_{off}$ values can be found in Supplementary Table 23.

| ***n*** | **$K_{D-1}$ (µM)** | **$K_{D-2}$ (µM)** | **$K_{D-3}$ (µM)** |
|---|---|---|---|
| 9 | 655 ± 47 | 125 ± 24 | 24 ± 5 |

Values were provided as mean ± s.d. The other experimental conditions were the same as those stated in Methods. Source data are provided as a Source Data file.

**Supplementary Table 26. WDR5-released durations ($\tau_{on}$) and WDR5-captured durations ($\tau_{off}$) for three binding events and for all [WDR5] values at a transmembrane potential of -40 mV.** $\tau_{on}$ are mean values for the single-exponential distribution of the WDR5-release duration histogram. $\tau_{on-i}$ values were calculated with the equation $\tau_{on-1} = \tau_{on}/P_1$, where $P_1$ is the event probability of the short-lived events for each experiment. $\tau_{on-2}$ and $\tau_{on-3}$ were calculated using the same equation and respective probabilities. The mean probabilities were listed in Supplementary Table 27. $\tau_{off}$ values are the mean values for the three-exponential distribution of the WDR5-captured duration histograms acquired at different [WDR5] values. All histogram fittings were conducted using a semilogarithmic representation. The maximum likelihood method[5, 9] and logarithm likelihood ratio (LLR)[10-12] tests were used for all fittings to determine the best multi-exponential distribution model (Methods).

| *n* | [WDR5] (µM) | $\tau_{on}$ (s) | $\tau_{on-1}$ (s) | $\tau_{on-2}$ (s) | $\tau_{on-3}$ (s) | $\tau_{off-1}$ (s) | $\tau_{off-2}$ (s) | $\tau_{off-3}$ (s) |
|---|---|---|---|---|---|---|---|---|
| 3 | 0.09 | 21 ± 1 | 38 ± 3 | 74 ± 5 | 165 ± 15 | 0.011 ± 0.002 | 0.13 ± 0.06 | 1.4 ± 0.5 |
| 3 | 0.5 | 4.1 ± 0.7 | 7.4 ± 1.3 | 15 ± 3 | 27 ± 2 | 0.010 ± 0.002 | 0.12 ± 0.03 | 1.5 ± 0.6 |
| 3 | 0.8 | 2.6 ± 0.3 | 4.6 ± 0.7 | 8 ± 1 | 18 ± 2 | 0.012 ± 0.003 | 0.12 ± 0.04 | 2.1 ± 0.7 |
| 3 | 4 | 0.62 ± 0.05 | 1.1 ± 0.1 | 1.9 ± 0.2 | 4.0 ± 0.9 | 0.014 ± 0.002 | 0.13 ± 0.08 | 2.0 ± 0.5 |
| 3 | 8 | 0.28 ± 0.03 | 0.49 ± 0.05 | 1.0 ± 0.1 | 1.9 ± 0.2 | 0.011 ± 0.005 | 0.09 ± 0.03 | 1.6 ± 0.7 |

Values were provided as mean ± s.d. *n* is the number of independently reconstituted nanopores. The other experimental conditions were the same as those stated in Methods. Source data are provided as a Source Data file.

**Supplementary Table 27. The probability distribution of the three binding events noted with WDR5-MLL4$_{Win}$ interactions at a transmembrane potential of -40 mV.** Individual experimental values were derived using event-list histograms in ClampFit (Axon) and fittings of event histograms in a semilogarithmic representation. The maximum likelihood method[5, 9] and logarithm likelihood ratio (LLR)[10-12] tests were used for all fittings to determine the best multi-exponential distribution model (Methods).

| ***n*** | **[WDR5] (µM)** | $P_1$ | $P_2$ | $P_3$ |
|---|---|---|---|---|
| 3 | 0.09 | 0.57 ± 0.10 | 0.29 ± 0.08 | 0.11 ± 0.04 |
| 3 | 0.5 | 0.56 ± 0.08 | 0.27 ± 0.06 | 0.18 ± 0.09 |
| 3 | 0.8 | 0.55 ± 0.07 | 0.31 ± 0.03 | 0.14 ± 0.05 |
| 3 | 4 | 0.55 ± 0.05 | 0.32 ± 0.08 | 0.12 ± 0.05 |
| 3 | 8 | 0.59 ± 0.11 | 0.28 ± 0.07 | 0.15 ± 0.07 |

Values were provided as mean ± s.d. from *n*, the number of independently reconstituted nanopores. $P_1$, $P_2$, and $P_3$ are the event probabilities of the short-, medium-, and long-lived events, respectively. The other experimental conditions were the same as those stated in Methods. Source data are provided as a Source Data file.

**Supplementary Table 28. The association and dissociation rate constants of the three binding events at a transmembrane potential of -40 mV.** The association rate constants of the short-lived events, $k_{on-1}$, were determined using the equation: $k_{on-1} = 1/(\tau_{on-1}[WDR5])$. The association rate constants of the medium-lived events, $k_{on-2}$, and long-lived events, $k_{on-3}$, were determined in the same way. $\tau_{on-i}$ values are provided in Table 26. The dissociation rate constants of the short-lived events were determined using the equation: $k_{off-1} = 1/\tau_{off-1}$. We used the same approach for determining the dissociation rate constants of the medium-lived events, $k_{off-2}$, and long-lived events, $k_{off-3}$. $\tau_{off-i}$ values are given in Table 26. Values were provided as mean ± s.d. *n* is number of independently reconstituted nanopores. The other experimental conditions were the same as those stated in Methods. Source data are provided as a Source Data file.

| *n* | [WDR5] (μM) | $k_{on-1} \times 10^{-5}$ ($M^{-1}s^{-1}$) | $k_{on-2} \times 10^{-5}$ ($M^{-1}s^{-1}$) | $k_{on-3} \times 10^{-4}$ ($M^{-1}s^{-1}$) | $k_{off-1}$ ($s^{-1}$) | $k_{off-2}$ ($s^{-1}$) | $k_{off-3}$ ($s^{-1}$) |
|---|---|---|---|---|---|---|---|
| 3 | 0.09 | 2.6 ± 1.1 | 1.5 ± 0.9 | 6.8 ± 0.6 | 98 ± 21 | 8.4 ± 3.0 | 0.76 ± 0.26 |
| 3 | 0.5 | 2.8 ± 0.5 | 1.3 ± 0.2 | 6.5 ± 0.4 | 98 ± 19 | 8.8 ± 2.1 | 0.64 ± 0.22 |
| 3 | 0.8 | 2.7 ± 0.6 | 1.2 ± 0.2 | 6.9 ± 0.9 | 86 ± 18 | 9.4 ± 3.5 | 0.50 ± 0.15 |
| 3 | 4 | 2.3 ± 0.8 | 1.3 ± 0.1 | 6.2 ± 0.3 | 72 ± 8 | 10 ± 5 | 0.53 ± 0.14 |
| 3 | 8 | 3.0 ± 0.4 | 1.5 ± 0.1 | 7.5 ± 0.8 | 75 ± 18 | 12 ± 4 | 0.76 ± 0.21 |

**Supplementary Table 29. The association and dissociation rate constants of the three distinctive binding events of $MLL4_{Win}$-WDR5 interactions at a transmembrane potential of -40 mV.** $k_{on}$ values were derived as the slope of the linear fits in Supplementary Fig. 22a. $k_{off}$ values are the axis intercepts of the horizontal line fits in Supplementary Fig. 22b.

| $k_{on-1} \times 10^{-5}$ ($M^{-1}s^{-1}$) | $k_{on-2} \times 10^{-5}$ ($M^{-1}s^{-1}$) | $k_{on-3} \times 10^{-4}$ ($M^{-1}s^{-1}$) | $k_{off-1}$ ($s^{-1}$) | $k_{off-2}$ ($s^{-1}$) | $k_{off-3}$ ($s^{-1}$) |
|---|---|---|---|---|---|
| 2.7 ± 0.1 | 1.4 ± 0.6 | 6.7 ± 0.2 | 89 ± 3 | 9.6 ± 0.5 | 0.65 ± 0.12 |

Values were provided as mean ± s.e.m. The other experimental conditions were the same as those stated in Methods. Source data are provided as a Source Data file.

**Supplementary Table 30**. **The equilibrium dissociation constants for the three events of MLL4$_{Win}$ -WDR5 interactions at a transmembrane potential of -40 mV.** $K_D$ values were determined using the equation: $K_D = k_{off}/k_{on}$. $K_{D\text{-}1}$, $K_{D\text{-}2}$, and $K_{D\text{-}3}$, are the equilibrium dissociation constants of the short-, medium-, and long-lived WDR5-captured events, respectively. The respective $k_{on}$ and $k_{off}$ values can be found in Supplementary Table 28. Source data are provided as a Source Data file.

| n | $K_{D\text{-}1}$ (µM) | $K_{D\text{-}2}$ (µM) | $K_{D\text{-}3}$ (µM) |
|---|---|---|---|
| 15 | 320 ± 24 | 70 ± 16 | 9.4 ± 1.5 |

Values were provided as mean ± s.d. *n* is number of independently reconstituted nanopores. The other experimental conditions were the same as those stated in Methods. Source data are provided as a Source Data file.

**Supplementary Table 31. WDR5-released durations ($\tau_{on}$) and association rate constants of the three binding events at various transmembrane potentials, $\Delta U$.** All values were calculated at 8 µM WDR5. $\tau_{on}$ values were calculated as in Supplementary Table 11. The mean probabilities were listed in Supplementary Table 33. The association rate constants of the short-lived events, $k_{on-1}$, were determined using the equation $k_{on-1} = 1/(\tau_{on-1}[WDR5])$. The association rate constants of the medium-lived events, $k_{on-2}$, and long-lived events, $k_{on-3}$, were determined in the same way. Values were provided as mean ± s.d. *n* is the number of independently reconstituted nanopores. The other experimental conditions were the same as those stated in Methods. Source data are provided as a Source Data file.

| *n* | $\Delta U$ (mV) | $\tau_{on}$ (s) | $\tau_{on-1}$ (s) | $\tau_{on-2}$ (s) | $\tau_{on-3}$ (s) | $k_{on-1} \times 10^{-5}$ ($M^{-1}s^{-1}$) | $k_{on-2} \times 10^{-5}$ ($M^{-1}s^{-1}$) | $k_{on-3} \times 10^{-4}$ ($M^{-1}s^{-1}$) |
|---|---|---|---|---|---|---|---|---|
| 3 | -10 | 0.74 ± 0.10 | 1.3 ± 0.1 | 2.5 ± 0.2 | 6.2 ± 0.5 | 0.87 ± 0.07 | 0.50 ± 0.04 | 2.0 ± 0.2 |
| 3 | -20 | 0.55 ± 0.10 | 0.96 ± 0.17 | 1.9 ± 0.3 | 3.7 ± 0.7 | 1.3 ± 0.3 | 0.68 ± 0.13 | 3.5 ± 0.7 |
| 3 | -40 | 0.28 ± 0.03 | 0.49 ± 0.05 | 1.0 ± 0.1 | 1.9 ± 0.2 | 3.0 ± 0.4 | 1.5 ± 0.1 | 7.5 ± 0.8 |

**Supplementary Table 32. WDR5-captured durations ($\tau_{off}$) and dissociation rate constants of the three binding events at varying transmembrane potentials, $\Delta U$.** All values were calculated at 8 µM WDR5. The fittings were conducted using a semilogarithmic representation. The dissociation rate constants of the short-lived events were determined using the equation: $k_{off-1} = 1/\tau_{off-1}$. We used the same equation for determining the dissociation rate constants of the medium-lived events, $k_{off-2}$, and long-lived events, $k_{off-3}$.

| n | $\Delta U$ (mV) | $\tau_{off-1}$ (s) | $\tau_{off-2}$ (s) | $\tau_{off-3}$ (s) | $k_{off-1}$ ($s^{-1}$) | $k_{off-2}$ ($s^{-1}$) | $k_{off-3}$ ($s^{-1}$) |
|---|---|---|---|---|---|---|---|
| 3 | -10 | 0.009 ± 0.002 | 0.10 ± 0.02 | 1.3 ± 0.7 | 84 ± 20 | 10 ± 2 | 0.81 ± 0.23 |
| 3 | -20 | 0.013 ± 0.004 | 0.071 ± 0.040 | 1.1 ± 0.2 | 82 ± 21 | 9.0 ± 1.1 | 0.89 ± 0.21 |
| 3 | -40 | 0.011 ± 0.005 | 0.092 ± 0.030 | 1.6 ± 0.7 | 75 ± 18 | 12 ± 4 | 0.76 ± 0.21 |

Values were provided as mean ± s.d. *n* is the number of independently reconstituted nanopores. The other experimental conditions were the same as those stated in Methods. Source data are provided as a Source Data file.

**Supplementary Table 33. The distribution probabilities of the three binding events at varying transmembrane potentials, Δ*U*.** All values were calculated at 8 μM WDR5. These events were differentiated by their WDR5-captured durations. Individual experimental values were derived using event-list histograms in ClampFit (Axon) and fittings of event histograms in a semi-logarithmic representation. The maximum likelihood method[5, 9] and logarithm likelihood ratio (LLR)[10-12] tests were used for all fittings to determine the best multi-exponential distribution model (Methods).

| n | Δ*U* (mV) | $P_1$ | $P_2$ | $P_3$ |
|---|---|---|---|---|
| 3 | -10 | 0.58 ± 0.07 | 0.30 ± 0.05 | 0.12 ± 0.04 |
| 3 | -20 | 0.57 ± 0.04 | 0.31 ± 0.03 | 0.18 ± 0.07 |
| 3 | -40 | 0.59 ± 0.11 | 0.28 ± 0.07 | 0.15 ± 0.07 |

Values were provided as mean ± s.d. *n* is the number of independently reconstituted nanopores. $P_1$, $P_2$, and $P_3$ are the event probabilities of the short-, medium-, and long-lived events, respectively. The other experimental conditions were the same as those stated in Methods. Source data are provided as a Source Data file.

**Supplementary Table 34. The association rate constants at a zero transmembrane potential.** The $k_{on\text{-}i}$ (0) values were determined as the axis intercept at $\Delta U = 0$ of the plot in the main text, Fig. 5d. All values were acquired at 8 μM WDR5 added to the *cis* compartment.

| n | $k_{on-1}(0) \times 10^{-5}$ ($M^{-1}s^{-1}$) | $k_{on-2}(0) \times 10^{-5}$ ($M^{-1}s^{-1}$) | $k_{on-3}(0) \times 10^{-4}$ ($M^{-1}s^{-1}$) |
|---|---|---|---|
| 7 | 0.69 ± 0.02 | 0.36 ± 0.07 | 1.9 ± 0.2 |

Values were provided as mean ± s.e.m. *n* is the number of independently reconstituted nanopores. The other experimental conditions were the same as those stated in Methods. Source data are provided as a Source Data file.

**Supplementary Table 35. The reduction in activation free energies of WDR5-released events, $\Delta\Delta G_{\text{on-i}}$, at different transmembrane potentials, $\Delta U$, with respect to the value determined at a zero transmembrane potential.** All values were calculated at 8 μM WDR5. The differential activation free energies, $\Delta\Delta G_{\text{on-i}}$, were calculated using the following equation:

$$\ln(k_{on-i}) = \ln(k_{on-i}(0)) - \frac{\Delta\Delta G_{on}}{RT} \quad (1)$$

Here, i=1, 2, and 3 are subscripts denoting those parameters corresponding to short-, medium-, and long-lived binding events, respectively. $R$ is the general gas constant. $T$ denotes the absolute temperature. $k_{\text{on-i}}$ ($\Delta U$) and $k_{\text{on-i}}$ (0) are provided in Supplementary Table 31 and Table 34.

| $n$ | $\Delta U$ (mV) | $\Delta\Delta G_{\text{on-1}}$ (kcal/mol) | $\Delta\Delta G_{\text{on-2}}$ (kcal/mol) | $\Delta\Delta G_{\text{on-3}}$ (kcal/mol) |
|---|---|---|---|---|
| 3 | −10 | −0.20 ± 0.05 | −0.19 ± 0.04 | −0.21 ± 0.04 |
| 3 | −20 | −0.39 ± 0.07 | −0.39 ± 0.11 | −0.31 ± 0.09 |
| 3 | −40 | −0.77 ± 0.06 | −0.71 ± 0.05 | −0.71 ± 0.07 |

Values were provided as mean ± s.d. $n$ is the number of independently reconstituted nanopores. The other experimental conditions were the same as those stated in Methods. Source data are provided as a Source Data file.

**Supplementary Table 36. The relative charge of WDR5 determined by voltage dependence experiments.** All values were calculated at 8 μM WDR5. The association rate constant at a certain transmembrane potential, $\Delta U$, depends on an exponential function:

$$k_{on-i}(\Delta U) = k_{on-i}(0)e^{-\frac{q\Delta U}{k_B T}} \quad (2)$$

Here, $k_{on-i}(\Delta U)$ and $k_{on-i}(0)$ are the association rate constants at transmembrane potentials $\Delta U$ and 0 mV, respectively. Here, $k_B$ is the Boltzmann's constant and $T$ is the absolute temperature. $q$ is the effective charge. The charge values were extracted using slopes in Fig. 5d. The slope equals to $-\frac{q}{k_B T}$. In this case, $q = z$e, where $z$ and $e$ are the relative charge and elementary charge, respectively. $k_{on\text{-}i}$ ($\Delta U$) and $k_{on\text{-}i}$ (0) are provided in Supplementary Table 31 and Table 34. All values were acquired at 8 μM WDR5.

| $n$ | $z_1$ | $z_2$ | $z_3$ |
|---|---|---|---|
| 9 | 0.81 ± 0.07 | 0.76 ± 0.10 | 0.79 ± 0.12 |

Values were provided as mean ± s.e.m. $n$ is the number of independently reconstituted nanopores. The other experimental conditions were the same as those stated in Methods. Source data are provided as a Source Data file.

**Supplementary Table 37. The values of the mean normalized amplitude of $D92N_{WDR5}$-produced current blockades, $I/I_0$, at various [$D92N_{WDR5}$] values.** Here, $I_0$ and $I$ denote the single-channel current of the $D92N_{WDR5}$-released substate of $MLL4_{Win}$tFhuA and the amplitude of $D92N_{WDR5}$-produced current blockades, respectively. The $I/I_0$ ratio was then converted into percentage.

| *n* | [D92N] (µM) | Probability of smaller $I/I_0$ | Smaller $I/I_0$ (%) | Probability of larger $I/I_0$ | Larger $I/I_0$ (%) |
|---|---|---|---|---|---|
| 3 | 10 | 0.54 ± 0.03 | 60 ± 2 | 0.46 ± 0.02 | 71 ± 3 |

Values were provided as mean ± s.d. *n* is the number of independently reconstituted nanopores. The other experimental conditions were the same as those stated in Methods. Source data are provided as a Source Data file.

**Supplementary References.**


1. Dharmarajan, V., Lee, J.H., Patel, A., Skalnik, D.G. & Cosgrove, M.S. Structural basis for WDR5 interaction (Win) motif recognition in human SET1 family histone methyltransferases. *J. Biol. Chem.* **287**, 27275-27289 (2012).
2. Thakur, A.K. & Movileanu, L. Real-Time Measurement of Protein-Protein Interactions at Single-Molecule Resolution using a Biological Nanopore. *Nat. Biotechnol.* **37**, 96-101 (2019).
3. Larimi, M.G., Ha, J.H., Loh, S.N. & Movileanu, L. Insertion state of modular protein nanopores into a membrane. *Biochim. Biophys. Acta Biomembr.* **1863**, 183570 (2021).
4. Moss, G.W.J. & Moczydlowski, E. in Ion Channels - A Practical Approach, Edn. Second. (ed. R.H. Ashley) 69-112 (Oxford University Press, Oxford; 2002).
5. Colquhoun, D. & Sigworth, F.J. in Single-channel recording, Edn. 2nd. (ed. B. Sackmann, Neher,E.) 483-587 (Plenum Press, New York; 1995).
6. Colquhoun, D. & Hawkes, A.G. in Single-Channel Recording, Edn. 2nd. (eds. B. Sackmann & E. Neher) 397-482 (Plenum Publishers, New York; 1995).
7. Colquhoun, D. & Hawkes, A.G. in Single-Channel Recording, Edn. 2nd. (eds. B. Sackmann & E. Neher) 589-633 (Plenum Publishers, New York; 1995).
8. Zhang, P., Lee, H., Brunzelle, J.S. & Couture, J.F. The plasticity of WDR5 peptide-binding cleft enables the binding of the SET1 family of histone methyltransferases. *Nucleic Acids Res.* **40**, 4237-4246 (2012).
9. Colquhoun, D., Hatton, C.J. & Hawkes, A.G. The quality of maximum likelihood estimates of ion channel rate constants. *J. Physiol.* **547**, 699-728 (2003).
10. McManus, O.B., Blatz, A.L. & Magleby, K.L. Sampling, Log Binning, Fitting, and Plotting Durations of Open and Shut Intervals From Single Channels and the Effects of Noise. *Pflugers Arch.* **410**, 530-553 (1987).
11. McManus, O.B. & Magleby, K.L. Kinetic States and Modes of Single Large-Conductance Calcium-Activated Potassium Channels in Cultured Rat Skeletal-Muscle. *J. Physiol. (Lond.)* **402**, 79-120 (1988).
12. Movileanu, L., Cheley, S. & Bayley, H. Partitioning of Individual Flexible Polymers into a Nanoscopic Protein Pore. *Biophys. J.* **85**, 897-910 (2003).
